# Plutonium(III) versus uranium(III) and samarium(III) in small molecule activation chemistry

**DOI:** 10.1038/s41467-026-72229-7

**Published:** 2026-05-02

**Authors:** Megan Keener, Thayalan Rajeshkumar, Cambell S. Conour, Joshua J. Woods, Laurent Maron, Polly L. Arnold

**Affiliations:** 1 Chemical Sciences Division, Lawrence Berkeley National Laboratory, Berkeley and Department of Chemistry, University of California, Berkeley, CA USA; 2 Laboratoire de Physique et Chimie des Nano-objets, Institut National des Sciences Appliquées, Toulouse, CEDEX 4 France

**Keywords:** Chemical bonding, Chemical bonding, Synthetic chemistry methodology

## Abstract

We report the Pu^III^ complex, [Pu^III^(Cp^Me4^)_3_] (**1-Pu**), and demonstrate its differences in small molecule reactivity compared to the U^III^ and Sm^III^ analogs, [U^III^(Cp^Me4^)_3_] (**1-U**) and [Sm^III^(Cp^Me4^)_3_] (**1-Sm**), respectively. **1-Pu** reductively cleaves the small molecule (PhS)_2_, affording a Pu^III^ complex, [{Pu^III^(Cp^Me4^)_2_}_2_(*μ*-SPh)_2_] (**2-Pu**), while retaining the Pu^III^ center and eliminating (Cp^Me4^)_2_ as a by-product, a fingerprint of a sterically induced reduction (SIR) reaction. Sm is often used as a surrogate for Pu, but the analogous [Sm^III^(Cp^Me4^)_3_], (**1-Sm**), is unreactive. The (PhS)_2_ cleavage by **1-U** proceeds solely via a metal-based oxidation (i.e., U^III^ → U^IV^), to form [U^IV^(Cp^Me4^)_3_(SPh)] (**3-U**). Only **1-U** reacts with (PhHN)_2_, affording the reductive cleavage product, [U^IV^(Cp^Me4^)_3_(NHPh)] (**4-U**). The difference in reactivity of **1-Pu** compared to complexes **1-Sm** and **1-U** was unexpected, and since SIR chemistry can enable complexes to participate in otherwise impossible reductive transformation of substrates, this reinforces the importance of studying small molecule reactivity with the transuranic elements.

## Abstract (web summary)

The activation of small molecules by organometallic complexes, as far as uranium has transformed our understanding of electronic structure and bonding. Here, the authors report a transuranic Pu(III) complex and demonstrate its differences in small molecule reactivity compared to the U(III) and Sm(III) analogs.

## Introduction

The reductive activation of small molecules by complexes across the periodic table has greatly advanced our understanding of molecular electronic structure and bonding^1,2^. In the *f*-block, reactions involving the reducing Ln^II^ (Sm^II^, Eu^II^, Yb^II^) and An^III^ (Th^III^, U^III^) complexes, have generated otherwise inaccessible and interesting new molecules and exposed new types of reaction pathways^3–36^. In these examples, the complexes achieve small molecule activation by using a metal-based electron.

**Fig. 1:**
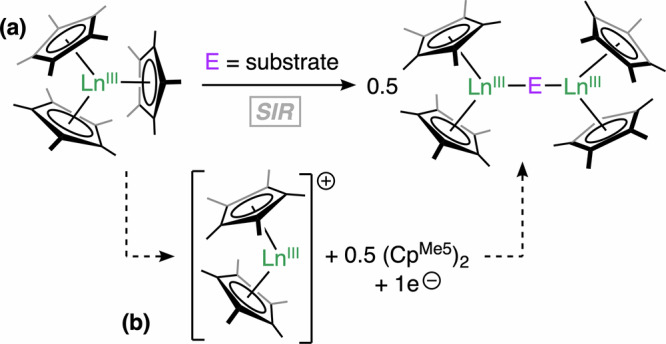
Reductive cleavage of a substrate by a tris-Cp^Me5^ Ln complex. **a** [Ln^III^(Cp^Me5^)_3_] undergoes a reductive cleavage of substrate “E” via a sterically induced reduction (SIR) reaction. **b** The SIR reaction proceeds via elimination of half an equiv. of the Cp^Me5^ dimer, (Cp^Me5^)_2_, which provides a reducing electron to the substrate.

For the non-oxidizable Ln^III^’s (all except Ce^III^, which can achieve the +4 oxidation state), small molecule reduction is achieved by generating sterically crowded complexes from which the reducing electron is provided by homolysis of a Ln^III^–ligand bond. This was first noted for the sterically congested Sm^III^ complex, [Sm^III^(Cp^Me5^)_3_] (Cp^Me5^ = C_5_Me_5_)^37–39^, in which one electron from the normally inert Sm–Cp^Me5^ bond enables the complex to act as a one-electron reductant, eliminating half an equivalent of (Cp^Me5^)_2_ dimer as a by-product (Fig. 1). This type of reactivity was termed as a sterically induced reduction (SIR). It is important to note that the slightly less sterically congested Cp^Me4^ systems, [Ln^III^(Cp^Me4^)_3_], do not exhibit SIR reactivity. Even though the mechanism has yet to be studied, it has since been recognized in other Ln^III^ systems with bulky ligands^40–43^.

In recent years, there has been a renewed interest in transuranium and organometallic 5 *f* element chemistry^44–60^. When traversing the actinide series, the +3 oxidation state becomes less reducing^61^, but the redox potentials are strongly ligand-dependent, and there is still very little known about how to properly tune these systems. For Pu, the 5 *f* valence electrons exist within the boundary between localized and itinerant states, which makes Pu a particularly fascinating metal in terms of redox, as four different oxidation states can exist simultaneously in solution^61^. This unpredictable redox behavior though, can lead to problematic assumptions about actinide reactivity and calls into question the use of Ln congeners as surrogates for these heavy elements.

Despite these advances, the few reactivity studies that have been reported^47,62^ primarily involve strong oxidizing/reducing agents^45,49,50,56,60,63–66^ or PCET reactions^63,67^. The redox reactivity of transuranic complexes with small molecules is notable by its absence. To date, the oxidation of the Np^III^ amide complex, [(TREN)^TIPS^Np^III^] to [(TREN)^TIPS^Np^V^O] by N_2_O is the only reaction reported that could be classified as a small molecule activation reaction, and no mechanism was suggested^46^.

Tris-Cp *f*-block complexes offer a good opportunity to identify the conditions needed to induce SIR chemistry, since small changes in the ligand set are possible across the 4 *f* and 5 *f* series^68^. Suggested key features that distinguish SIR reactivity include a significant displacement of the Cp-methyl groups away from the metal center^69^, or a long metal–ligand bond, either in the reagent or a key intermediate^70–73^.

Here, we report the isolation and characterization of a Pu^III^ cyclopentadienyl complex, [Pu^III^(Cp^Me4^)_3_] (**1-Pu**), and investigate its ability to effect reductive cleavage of the small molecules diphenyldisulfide, (PhS)_2_, and 1,2-diphenylhydrazine, (PhHN)_2_. We show that **1-Pu** reacts only with (PhS)_2_, yielding a new Pu^III^ bridging bis-thiolato complex, [{Pu^III^(Cp^Me4^)_2_}_2_(*μ*-SPh)_2_] (**2-Pu**). This contrasts both the type of reactivity observed with the U^III^ congener, [U^III^(Cp^Me4^)_3_] (**1-U**)^74^, which results in oxidation of the metal, and the lack of reactivity observed with the Sm^III^ congener, [Sm^III^(Cp^Me4^)_3_] (**1-Sm**)^75^. The observed reactivity for **1-Pu** is attributed to SIR reactivity, overall retaining the Pu^III^ oxidation state, but exploiting additional electrostatic interactions with the substrate that are not available to **1-Sm**, and without which, **1-Pu** would be predicted to be even less likely to react than **1-Sm**. Complex **1-U**, containing the more strongly reducing U^III^ cation, reduces both (PhS)_2_ and (PhHN)_2_ through a metal-promoted reductive cleavage pathway, yielding unique U^IV^ terminal thiolato and amido complexes, [U^IV^(Cp^Me4^)_3_(SPh)] (**3-U**) and [U^IV^(Cp^Me4^)_3_(NHPh)] (**4-U**), respectively. Complex **1-Sm**, which cannot access Sm^IV^, but could homolytically cleave one metal–Cp bond to reduce (PhS)_2_ in the same manner as [Sm^III^(Cp^Me5^)_3_]^76^, does not. DFT calculations support the experimental findings in which the reductive transformation for **1-Pu** is enabled through the formation of an unstable, overcrowded Pu^III^ intermediate, which is not accessible for the Sm^III^ congener, which then achieves the reductive activation via a SIR reaction that eliminates half an equivalent of (Cp^Me4^)_2_, forming complex **2-Pu**. These small-molecule activation studies provide key insights into the previously unexpected reactivity differences between the transuranic and lanthanide elements.

## Results

**Fig. 2:**
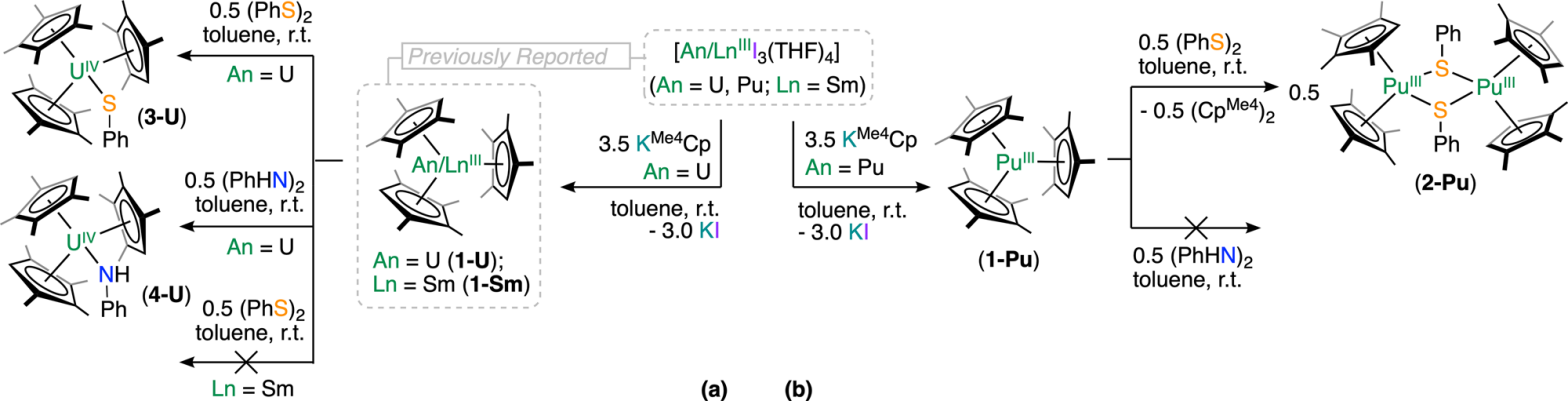
Synthesis of complexes 1-Pu, 1-U^74^, 1-Sm^75^, 2-Pu, 3-U, and 4-U. **a** The compounds [U^III^I_3_(THF)_4_] and [Sm^III^I_3_(THF)_4_] react via salt metathesis reactions with KCp^Me4^, affording complexes **1-U** and **1-Sm. 1-U** reacts with (PhS)_2_ or (PhHN)_2_ to afford U^IV^ complexes **3-U** and **4-U**, respectively. Complex **1-Sm** does not react with (PhS)_2_. **b** Compound [Pu^III^I_3_(THF)_4_] reacts analogously to the U and Sm analogs via salt metathesis reactions with KCp^Me4^, affording complex **1-Pu. 1-Pu** reacts with (PhS)_2_, but not (PhHN)_2_, to afford the dimeric Pu^III^ complex **2-Pu**.

**Fig. 3:**
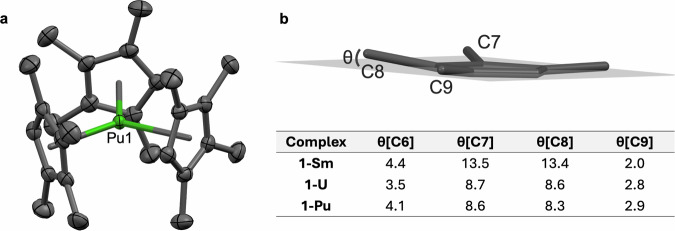
Solid-state molecular structure of 1-Pu and out-of-plane methyl displacements for the Cp^Me4^ ligands of 1-Sm, 1-U, and 1-Pu. **a** Complex [Pu^III^(Cp^Me4^)_3_] **1-Pu**, with thermal ellipsoids drawn at the 50% probability level. Plutonium (Pu) is shown in green and carbon (C) in dark grey. Hydrogen atoms are omitted for clarity. **b** top: the average ring carbon plane (grey) defined by the five C_Cp_ carbon atoms in each ligand in **1-Pu**; bottom: The out-of-plane methyl-displacement (θ) for the complexes **1**.

First, we synthesized the tris-Cp^Me4^ Pu^III^ complex, [Pu^III^(Cp^Me4^)_3_] (Cp^Me4^ = C_5_H(CH_3_)_4_) (**1-Pu**), from a salt-metathesis route (Fig. 2). The synthetic procedure was optimized by first preparing the previously reported U^III^ complex, [U^III^(Cp^Me4^)_3_] (**1-U**), as a surrogate for Pu^III^. Addition of 3.5 equiv. of KCp^Me4^ to 1.0 equiv. of [Pu^III^I_3_(THF)_4_]^53^ in toluene at room temperature results in an immediate color change from pale purple to teal-green, from which [Pu^III^(Cp^Me4^)_3_] (**1-Pu**) was isolated as a microcrystalline solid after work-up. Single crystals suitable for X-ray diffraction studies (Fig. 3a) were obtained from a concentrated Et_2_O solution at −40 °C in an 85 % yield. **1-Pu** is stable in toluene solution and the solid-state at room temperature for at least two months, in which the stability was monitored by ^1^H NMR spectroscopy (Supplementary Fig. 7). On one occasion, we isolated a hydrolysis product, [Pu^III^(Cp^Me4^)_2_}_2_(*μ*-OH)_2_] (**5-Pu**, Supplementary Fig. 22), presumed to arise from protonolysis of **1-Pu** with adventitious H_2_O in the negative pressure glovebox atmosphere. The room temperature ^1^H NMR spectrum of isolated **1-Pu** displays resonances at *δ* 24.1, 0.76, and −0.09 ppm, corresponding to the Cp^Me4^ ligands (Supplementary Fig. 6). The UV-Vis-NIR solid and solution state absorption spectra for **1-Pu** show intense absorption bands with a λ_max_ = 632 nm (solution: ε = ∼500 M^−^^1^ cm^−^^1^), consistent with a Laporte-allowed 5 f → 6 d transition^77^. There are also several weaker absorption bands between 850–1700 nm assigned to 5 f → 5 f Laporte-forbidden transitions (Supplementary Fig. 23-25). Time-dependent DFT (TDDFT) calculations for the optimized structure of **1-Pu** displays various 5 f → 6 d transitions around 575 nm, consistent with the experimentally derived λ_max_ value of 632 nm; the underestimation of the wavelength by TDDFT is well-documented^78,79^. Additionally, 5 f → 5 f Laporte-forbidden transitions were found in the 950–2000 nm region (Supplementary Table 2), consistent with the experimental values. Similar absorptions have been reported for the Pu^III^ complexes, [Pu^III^(Cp’)_3_] (Cp’ = {C_5_H_4_SiMe_3_}^1-^): λ_max_ = 585 nm; ε = 500 M^−1^ cm^-1^)^66^ and [Pu^III^(Cp”)_3_]: λ_max_ = 583 nm; ε = ∼600 M^−1^ cm^−1^)^49^.

In the solid-state molecular structure of **1-Pu** (Fig. 3), the Pu^III^ ion sits on a three-fold symmetry axis and is isostructural with other [M^III^(Cp^Me4^)_3_] complexes (M = Th, U, Am, La, Ce, Pr, Nd, Gd, Sm, Tb, Yb, Lu, Y)^80–82^. Complex **1-Pu** is the third example of a crystallographically characterized tris-Cp complex of Pu, although meaningful comparisons with the previously reported complex, [Pu^III^(Cp”)_3_] (Cp” = {C_5_H_3_(1,3-SiMe_3_)_2_}^1-^), cannot be made due to extreme disorder within the structure^49^. **1-Pu** shows classical *η*^5^-bonding to each planar Cp^Me4^ ligand with Pu1–C_Cp_ [2.851(2), 2.853(2), 2.754(2), 2.755(2), 2.697(2) Å] and a Pu1–centroid distance of 2.5096(11) Å, (Table 1). These metrics for **1-Pu** are similar to those in the parent complex, [Pu^III^(Cp)_3_] (Cp = {C_5_H_5_}^1-^) [C–C_Cp_: 2.644–2.720, 2.769–2.915; Pu1–centroid: 2.392, 2.565, 2.574 Å]^48^.

**Table 1:** Selected distances (Å) for complexes 1-Pu, 1-U, 2-Pu, 3-U, and 4-U

Complex	1-Pu	1-U^82^	2-Pu	3-U	4-U
An–C_Cp_	2.697(2)–2.853(2)	2.723–2.860	2.665(5)–2.806(5); 2.683(5)–2.757(6)	2.690(3)–2.934(2)	2.705(5)–2.962(4)
An–centroid	2.5096(11)	2.523	2.459(2); 2.449(2)	2.5333(15); 2.5198(15); 2.5535(15)	2.5460(19); 2.5734(19); 2.535(2)
An–SPh	--	--	2.8428(14)	2.7241(9)	--
U–NHPh	--	--	--	--	2.275(4)

Reductive cleavage studies: Due to Pu^III^ being redox active, albeit a weaker reductant than U^III^^61^, it was not immediately clear if **1-Pu** and **1-U** would have the capacity for small molecule reduction by a metal-promoted reduction or ligand-based SIR reactivity. Consequently, we first sought indicators for SIR reactivity from structural parameters; both longer than average M–Cp centroid distances, and large displacement of the M–Cp groups on the Cp^Me5^ ligands away from the metal (Fig. 3b), which have been observed in complexes that exhibit SIR reactivity^83^. The ionic radius of the An^III^ decreases from U^III^ [1.025 Å] to Pu^III^ [1.00 Å]^84^; therefore, the An–centroid distance should also decrease by the same amount. However, in complexes **1-U** and **1-Pu**, the decrease in the An–centroid distance is smaller (from **1-U** [2.523 Å] to **1-Pu** [2.5096(11) Å], Table 1), suggesting there may be a small, steric-congestion related lengthening of the Pu-Cp^Me4^ bond. The displacements of the M–Cp^Me4^ groups in **1-U** and **1-Pu** are small and comparable (**1-U**: θ = 2.8, 3.5, 8.8, 8.6°; **1-Pu**: θ = 2.9, 4.1, 8.6, and 8.3°, Fig. 3b; Supplementary Eqn. 1), and are significantly less than those in the Ln^III^ tris-Cp^Me5^ complexes, [Ln^III^(Cp^Me5^)_3_] (Ln = La, Nd, Sm) [θ_max_ = 19.4–20.3°]^83^, which do exhibit SIR. Together, these data suggest that metal-centered reductions, not SIR, would be more likely for both **1-U** and **1-Pu** based on metrical parameters alone.

We chose the small molecules diphenyldisulfide (PhS)_2_ and 1,2-diphenylhydrazine (PhHN)_2_ to provide insight into the differences in reductive cleavage mechanisms for **1-Pu,** **1-U**^74^, and **1-Sm**^75^ as they are safe to handle in a transuranic chemistry facility and provide contrast in ionic size and reduction potential^85,86^.

First, the addition of 0.5 equiv. of (PhS)_2_ to a solution of **1-Pu** results in a dramatic color change from teal green to dark purple over 7 days. The reaction mixture was monitored by ^1^H NMR spectroscopy, which showed the consumption of **1-Pu** and the formation of new resonances, including the product, [{Pu^III^(Cp^Me4^)_2_}_2_(*μ*-SPh)_2_] (**2-Pu**) and the signature SIR by-product, (Cp^Me4^)_2_^74^ (Supplementary Fig. 11). We also looked for, but did not observe, PhS-Cp^Me4^ which would have been a by-product if a σ-bond metathesis reaction pathway had occurred instead^39,68,76^. We note that the control experiment, where (PhS)_2_ and KCp^Me4^ react over five days, as monitored by ^1^H NMR spectroscopy (Supplementary Fig. S17), yields a mixture of PhS-Cp^Me4^, unidentifiable material, and no (Cp^Me4^)_2_, indicating that complex **1-Pu** is required for reductive cleavage reactivity.

Dark purple single crystals suitable for X-Ray diffraction studies of **2-Pu** were obtained from a concentrated *n*-hexanes solution at −40 °C in 70 % yield. The solid-state molecular structure of **2-Pu** shows a dimeric Pu^III^ compound with two [Pu^III^(Cp^Me4^)_2_]^+1^ units bridged symmetrically by two (*μ*-SPh) ligands.^−1^ The Pu1–centroid distances [2.459(2), 2.449(2) Å] are shorter than in the Pu^III^ starting material, **1-Pu** [2.5096(11) Å]. Comparisons of the Pu1–S1 bond distance [2.8428(14) Å] cannot be made at this time due to the absence of reported terminal or bridging Pu–chalcogenolates. Similar dimeric U^III^ complexes have also not been crystallographically characterized, precluding further comparisons. The only other crystallographically identified Pu–S interactions are observed in the imidodiphosphinochalcogenide, Pu[N(SPR_2_)_2_]^87^, 2,2′-biphenylenedithiophosphinic acid, Pu[S_2_P(C_6_H_5_)_2_]_3_(pyr)_2_^88^ complexes, and plutonium sulfide^89^.

The UV-Vis-NIR solid and solution state absorption spectra for complex **2-Pu** displays strong absorption bands with a λ_max_ = 566 nm (solution: ε = ∼900 M^−1^ cm^−1^), most likely consistent with a Laporte-allowed 5 f → 6 d transition. There are also several weaker absorption bands between 700–1700 nm assigned to 5 f → 5 f Laporte-forbidden transitions (Supplementary Fig. 26-28). The observed absorptions present in **2-Pu** are characteristic of Pu^III^ ions that contain Pu–organometallic bonding^49,53,57,66,90^. The assignments were confirmed by TDDFT calculations, displaying two 5 f → 6 d Laporte-allowed transitions with λ_max_ values at 513 and 558 nm, and Laporte-forbidden 5 f → 5 f transitions in the 1000–2000 nm range (Table S2). Other comparisons to previously reported complexes are difficult due to the lack of similarly characterized systems.

Addition of 0.5 equiv. of (PhS)_2_ to a solution of **1-Sm** in *d*_*8*_-toluene shows no evidence of a reaction, according to ^1^H NMR spectroscopy (Supplementary Fig. 14), which contrasts the reactivity previously reported for [Sm^III^(Cp^Me5^)_3_]^37–39,76^.

**Fig. 4:**
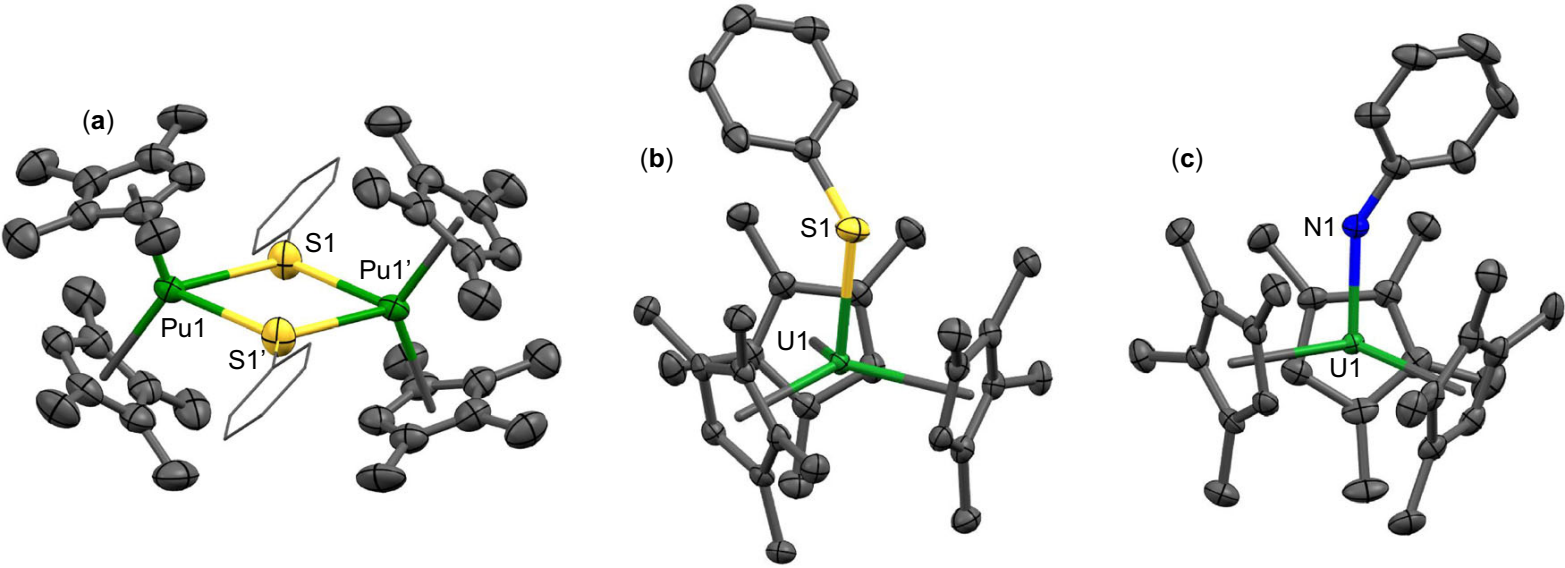
Solid-state molecular structures of 2-Pu, 3-U, and 4-U. **a** Complex [{Pu^III^(Cp^Me4^)_2_}_2_(*μ*-SPh)_2_], (**2-Pu**). **b** Complex [U^IV^(Cp^Me4^)_3_(SPh)], (**3-U**). **c** Complex [U^IV^(Cp^Me4^)_3_(NHPh)], (**4-U**). Thermal ellipsoids drawn at the 50% probability level. Hydrogen atoms are omitted for clarity. The phenyl groups in **a** are drawn as a wireframe with a minor component of disorder omitted for clarity. Plutonium (Pu) and uranium (U) are shown in green, sulfur (S) in yellow, nitrogen (N) in blue, and carbon (C) in dark grey.

Addition of 0.5 equiv. of (PhS)_2_ to a solution of **1-U** in *d*_*8*_-toluene results in the immediate consumption of the starting materials as evidenced by ^1^H NMR spectroscopy (Supplementary Fig. 15). Single crystals suitable for X-ray diffraction studies were obtained from a concentrated Et_2_O solution at −40 °C and identified as the U^IV^ terminal-thiolato complex, [U^IV^(Cp^Me4^)_3_(SPh)] (**3-U**) (Fig. 4b) in 81% yield. The solid-state molecular structure of **3-U** shows a monometallic U^IV^ complex with three Cp^Me4^ ancillary ligands and a terminal –SPh ligand. The U1–centroid [2.5333(15), 2.5198(15), 2.5535(15) Å] and unique U1–C_Cp_ distances [2.690(3)–2.934(2) Å] in **3-U** are similar to those in the **1-U** starting material [U1–centroid: 2.523 Å; U1–C_Cp_: 2.723–2.860 Å]^82^ and the U^IV^ complexes, [U^IV^(Cp^Me4^)_3_Cl] (U1–centroid: 2.520 Å; U1–C_Cp_: 2.658(11)–2.9111(10) Å)^91^ and [U^IV^(Cp^Me4^)_3_I] (U1–centroid: 2.52 Å; U1–C_Cp_: 2.669(4)–2.901(4) Å)^74^. The U1–SPh bond distance (2.7241(9) Å) is consistent with previously reported monometallic U^IV^–(SPh)_m_ (m = 1, 2, 4) complexes, [U^IV^(^Me5^Cp)_2_Me(SPh)] (U1–SPh: 2.7060(14) Å)^92^, [U^IV^(Cp^Me4^)_2_(SPh)_2_] (U1–SPh: 2.6845(7) and 2.6967(7) Å)^93^, and [U^IV^(SPh)_4_(pyr)_3_] (U1–SPh: 2.7169(17)–2.7639(17) Å)^94^.

Next, we compared the reactivity of complexes **1-Pu** and **1-U** with (PhHN)_2_. Addition of 0.5 equiv. of (PhHN)_2_ to complex **1-Pu** in *d*_*8*_-toluene at room temperature resulted in no reactivity of **1-Pu** over 14 days, as evidenced by ^1^H NMR spectroscopy (Supplementary Fig. 13). However, we did observe slight decomposition of (PhHN)_2_ to azobenzene (PhN)_2_ (31% yield) and other unidentifiable species. This decomposition of the substrate most likely stems from radiolysis reactions initiated by the Pu center^95^, as the control experiment without **1-Pu** resulted in no appreciable decomposition over the reaction timeframe.

Addition of 0.5 equiv. of (PhHN)_2_ to a solution of **1-U** in *d*_*8*_-toluene at room temperature results in the consumption of **1-U** and the formation of new resonances in the ^1^H NMR spectrum over the course of four days (Supplementary Fig. 16). Single crystals suitable for X-ray diffraction studies were identified as the U^IV^ terminal-amido complex, [U^IV^(Cp^Me4^)_3_(NHPh)] (**4-U**), and were obtained from a concentrated *n*-hexanes solution at −40 °C (Fig. 4c) in 88 % yield. The solid-state molecular structure of **4-U**, (Fig. 4c), displays a monometallic U^IV^ ion with a terminal –NHPh and three Cp^Me4^ ligands. The U1–NHPh bond distance (2.275(4) Å) is consistent with previously reported U^IV^–(NHPh)_m_ (m = 1, 2) complexes, [U^IV^(^Me5^Cp)_2_(hpp)(NHPh)] (U1–NHPh: 2.291 Å)^96^ and [U^IV^{1,3-(Me_3_C)_2_Cp}_2_(NHPh)_2_] (U-NHPh: N(1) 2.232(6) and N(2) 2.235(7) Å)^97^. The U1–centroid and U1–C_Cp_ distances found in **4-U** [2.5460(19), 2.5734(19), 2.535(2) Å; 2.705(5)–2.962(4)] are similar to those in **3-U** [2.5333(15), 2.5198(15), 2.5535(15) Å; 2.690(3)–2.934(2) Å].

The UV-Vis-NIR solid and solution state absorption spectra for complexes **3-U** and **4-U** display strong absorption bands with λ_max_ values of 422 nm (solution: ε = ∼3400 M^−1^ cm^−1^; Supplementary Fig. 29-31) and 398 nm (solution: ε = ∼3350 M^−1^ cm^−1^; Supplementary Fig. 32-34), respectively, consistent with Laporte-allowed 6 d → 5 f transitions. TDDFT calculations find λ_max_ values of 366 and 333 nm for complexes **3-U** and **4-U**, respectively, and are consistent with the experimentally determined values due to the well-known underestimation of the transition by TDDFT. There are also several weaker absorption bands between 700–1700 nm assigned to 5 f → 5 f Laporte-forbidden transitions (Table S3 and S4). The absorption data for **4-U** and **3-U** complexes are consistent with other U^IV^ systems^98–100^.

## Discussion

All the experimental spectroscopic data suggests that complex **1-Pu** reduces and cleaves (PhS)_2_ via a ligand-based SIR pathway, forming the bimetallic Pu^III^ complex, **2-Pu**, with elimination of half an equivalent of (Cp^Me4^)_2_, whereas complex **1-U** reduces and cleaves both (PhS)_2_ and (PhNH)_2_ via a metal-based reduction pathway, forming U^IV^ complexes, **3-U** and **4-U**, respectively. This is consistent with previous reports of U^III^-centered reductive cleavage by complexes, **1-U** and [U^III^(Cp^Me5^)_3_], with PhCl^74,101^. Furthermore, we found that the addition of excess (PhS)_2_ and (PhHN)_2_ to **3-U** and **4-U**, respectively, did not result in secondary reactions via an SIR pathway.

When comparing the metrical parameters (*vide supra*) to determine the predominant mechanistic pathway for complexes, **1-Pu,** **1-Sm**, and **1-U**, it is not obvious why **1-Pu** would react by SIR instead of the predicted metal-based reduction, and in particular why **1-Pu** reacts when **1-Sm** does not. The formation of the Pu^III^ complex, **2-Pu**, and (Cp^Me4^)_2_ dimer^74^ – rather than formation of a Pu^IV^ product – all point towards SIR reactivity. Yet, the slight relative lengthening of the An–Cp^Me4^ bond in **1-Pu** compared to **1-U**, and the insignificant differences in the displacement of the Cp^Me4^ groups away from the metal center (**1-Pu**: θ = 2.9, 4.1, 8.6, and 8.3° versus **1-U**: θ = 2.8, 3.5, 8.8, 8.6°), do not provide a clear indication as to whether **1-Pu** should reduce the (PhS)_2_ substrate by a metal-based reduction or ligand-based SIR reactivity. Additionally, the smaller ionic radius of Sm^III^ compared to Pu^III^ [0.958 Å vs 1.00 Å, respectively]^84^, and the significantly greater displacement of the Cp-methyl substituents away from the C_Cp_ plane in **1-Sm** [θ_max_ = 13.5°] suggest sterically congested **1-Sm** should be much more reactive than **1-Pu** with (PhS)_2_. We note that the sterically congested [M(Cp^Me5^)_3_] (M = La, Nd, Sm) complexes that undergo SIR reactivity have larger θ_max_ = 19.4–20.3° values. All these suggest that **1-Pu** is significantly less sterically encumbered, and one would expect **1-Sm** to be more reactive than **1-Pu**, in which a classical SIR reactivity by **1-Pu** would be unexpected. Therefore, the isolation of the Pu^III^ product led us to DFT computational analysis to help provide insights into these mechanistic pathways.

**Fig. 5:**
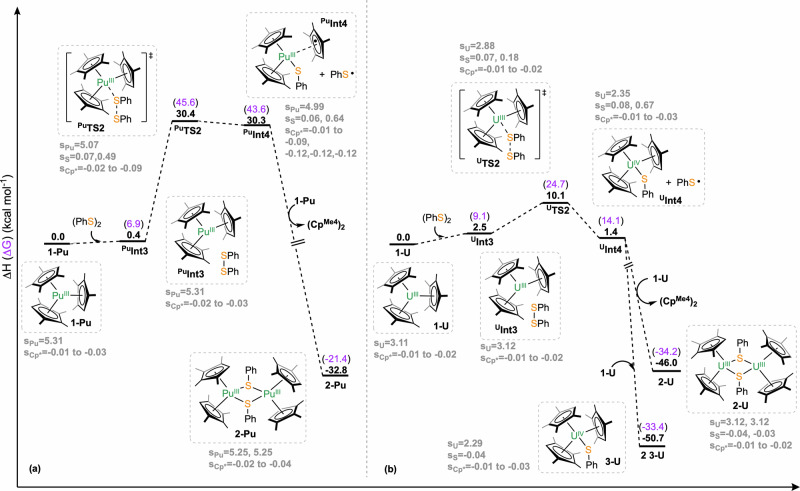
DFT computed pathways for the reaction of complexes 1-Pu and 1-U with (PhS)_2_. Computed (B3PW91 functional) enthalpy (ΔH; black) and Gibbs free energy (ΔG; purple) in kcal mol^−1^ at room temperature for the reactions of **a. 1-Pu** and **b. 1-U** with (PhS)_2_ is shown. The pair of lines indicates a spin-crossover. Spin densities are given in grey for important atoms and fragments.

DFT calculations (B3PW91 functional)^102^ were performed on the reactions of **1-Pu** and **1-U** with small molecule substrates, (PhS)_2_ and (PhHN)_2_, and of **1-Sm** with (PhS)_2_. The reaction profiles for complexes **1-Pu** and **1-U** with (PhS)_2_ (Fig. 5) demonstrate that the S–S bond activation reactions occur at the monomeric actinide complex. The energy barriers in the S–S bond activation for **1-Pu** and **1-U** are both favorable; however, they are stronger for **1-U** compared to **1-Pu** (i.e., both are kinetically accessible). For complex **1-U**, the reactivity with (PhS)_2_ and formation of complex **3-U** is thermodynamically favorable. For **1-Pu**, the most difficult step is coordination of the disulfide to the sterically congested Pu^III^ center, ^**Pu**^**TS2** (Fig. 5a). The steric congestion is still measurable in ^**Pu**^**Int4**, in both the methyl displacement [θ_max_ = 22°] and elongation of the Pu–centroid bond distances [2.55 Å] compared to those in ^**U**^**Int4** [θ_max_ = 14° and 2.54 Å]. Interestingly, the Cp^Me4^ ligand appears to retain the η^5^-coordination as it moves away from the Pu center, rather than undergoing a reduction in hapticity. From this, steric congestion is further relieved as the Pu-S covalent bond forms in ^**Pu**^**Int4** and the long-bonded Cp^Me4^ ligand dissociates via homolytic fission, releasing the Cp^Me4^ radical. After this, dimerization generates the thermodynamically favorable complex, **2-Pu**, and the dimerized (Cp^Me4^)_2_ by-product. We also sought a comparison with **1-Sm**, but it was not possible to locate any form of a stable [Sm^III^(Cp^Me4^)_3_(PhSSPh)] adduct (^**Sm**^**TS2**), or locate a transition state along the pathway, excluding a chance to computationally observe SIR reactivity of **1-Sm**. This surprising difference in reactivity of **1-Pu** and **1-Sm** is because the Pu(III) center is more polarizable, and can make use of d-orbitals, unlike **1-Sm**, in both forming an electrostatic interaction with the small molecule substrate and the dissociating Cp^Me4^ ligand.

**Fig. 6:**
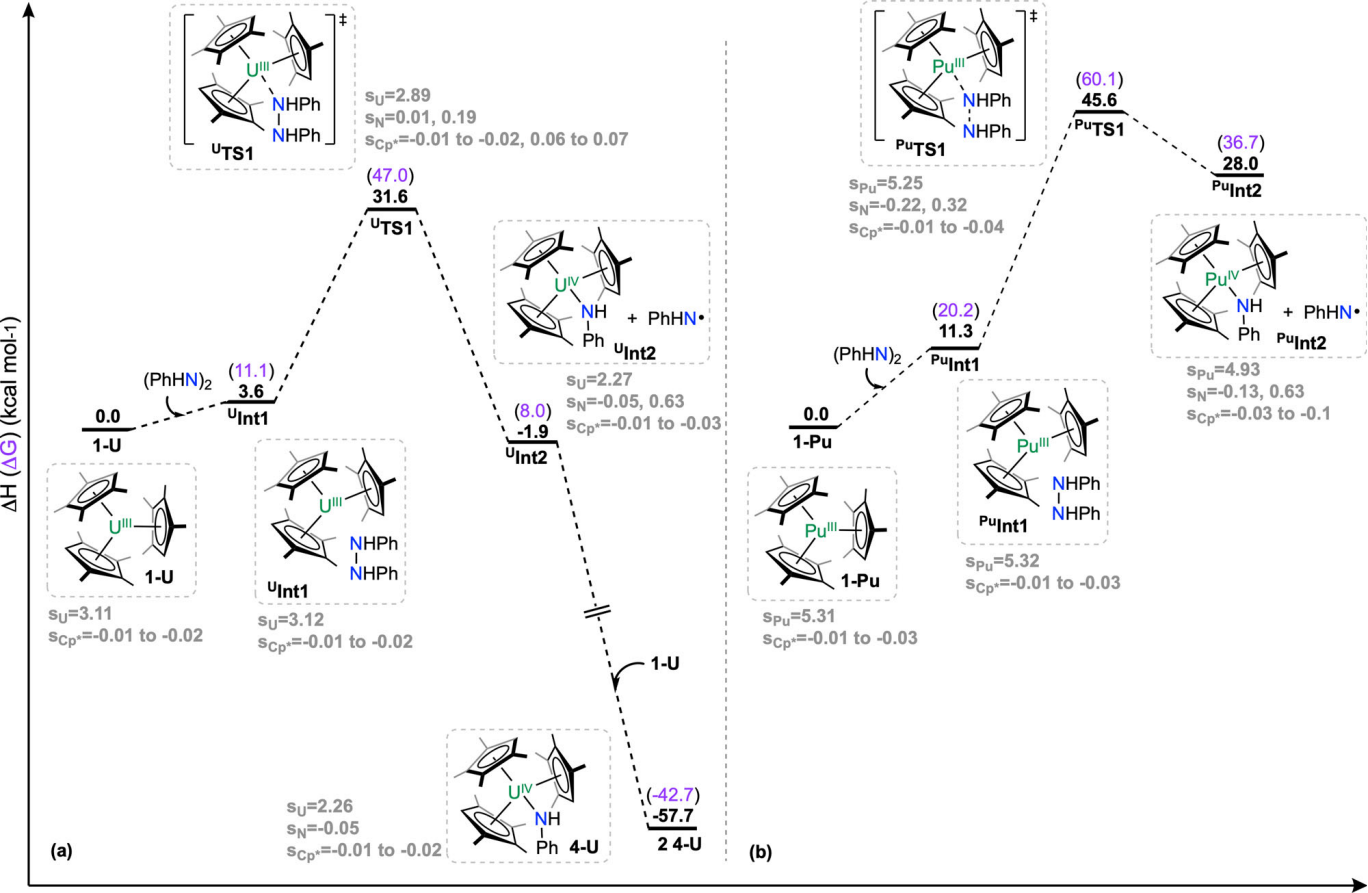
DFT computed pathways for the reaction of complexes 1-Pu and 1-U with (PhHN)_2_. Computed (B3PW91 functional) enthalpy (ΔH; black) and Gibbs free energy (ΔG; purple) in kcal mol^−1^ at room temperature for the reactions of **a. 1-Pu** and **b. 1-U** with (PhNH)_2_ is shown. The reaction profile for **1-Pu** is thermodynamically and kinetically inaccessible. The pair of lines indicates a spin-crossover. Spin densities are given in grey for important atoms and fragments.

The reaction profiles for the reactivity with (PhHN)_2_ (Fig. 6) are similar. As one might expect, the reaction of **1-Pu** with (PhHN)_2_ is both thermodynamically and kinetically not possible due to an inaccessibly high barrier for the N–N bond activation (^**Pu**^**TS1**; 45.6 kcal mol^−1^) and formation of the proposed product [Pu^IV^(Cp^Me4^)_3_(NHPh)] (^**Pu**^**Int2**; 28.0 kcal.mol^−^^1^; Fig. 4b). The approach of the substrate is also easier for the less-congested U^III^ complex; an intermediate (^**U**^**Int1**) with the N–N bond aligned directly above the U^III^ center is found with an energy of 3.6 kcal.mol^−1^, whereas only the phenyl ring of the reagent can approach the Pu^III^ center in the analogous ^**Pu**^**Int1** (11.3 kcal.mol^−1^) (Supplementary Fig. 36). It may be possible that the PhHN-analog of **2-Pu** (i.e., [{Pu^III^(Cp^Me4^)_2_}_2_(*μ*-NHPh)_2_]) is a thermodynamically stable compound; however, given that it is kinetically inaccessible in this chemistry, we did not calculate it.

In summary, we have reported the synthesis, structure, and small molecule cleavage chemistry of the new Pu^III^ complex, [Pu^III^(Cp^Me4^)_3_] (**1-Pu**). Interestingly, in transactinide chemistry, Sm^III^ is often used as a surrogate to predict Pu^III^ chemistry. However, based on the results for **1-Sm**, we would not have predicted **1-Pu** to react with (PhS)_2_, and if it did, then we would have predicted a metal-based reduction, like **1-U**. Instead, even though Pu^III^ is weakly reducing, **1-Pu** dissociates one Cp^Me4^ ligand to bind and then reductively cleave (PhS)_2_ by a SIR mechanism, leading to the isolation of a new bimetallic Pu^III^ reductive cleavage product, [{Pu^III^(Cp^Me4^)_2_}_2_(*μ*-SPh)_2_] (**2-Pu**), and formation of half an equivalent of (Cp^Me4^)_2_ dimer as a by-product. This suggests that the Pu-Cp^Me4^ bonding is more flexible than the Sm-Cp^Me4^ bonding. And yet, we do not observe evidence of ring-slippage to a lower hapticity Pu-Cp^Me4^ containing complex as has been observed in sterically crowded *f*-block chemistry. Comparative reductive cleavage studies with the previously reported U^III^ congener, [U^III^(Cp^Me4^)_3_] (**1-U**), show that, in contrast to **1-Pu**,it undergoes reductive cleavage of both (PhS)_2_ and (PhNH)_2_, resulting in products promoted by metal-oxidation of U^III^ to U^IV^, yielding complexes [U^IV^(Cp^Me4^)_3_(SPh)] (**3-U**) and [U^IV^(Cp^Me4^)_3_(NHPh)] (**4-U**), respectively.

The higher reactivity of Pu than Sm for SIR chemistry is a surprising and useful result, as it opens up new opportunities for the development of transuranic chemistry that avoids the use of undesirable strong reductants such as pyrophoric group 1 metals. It also opens up questions of whether transuranic compounds that were previously considered inert could exhibit unexpected or unwanted reactivity in the presence of sterically demanding donor molecules. Further electrochemical and reactivity studies are underway to search for additional factors, outside unusual structural characteristics, that can help determine transuranic small molecule reactivity and how they can be harnessed to target new transuranic electronic structures.

## Methods

### Materials and general considerations

Anhydrous solvents, including *n*-hexanes, Et_2_O, and toluene, were collected from an MBraun Solvent Purification System packed with columns of activated alumina, degassed, and stored over activated 4 Å molecular sieves prior to use. Deuterated solvents were purchased from Cambridge Isotope Laboratories: *d*_*8*_-toluene was distilled over potassium/benzophenone and freeze-degassed. All solvents were tested with a dilute THF solution of a ketyl radical (150 mg benzophenone in 20 mL of THF with an excess of K metal). Unless otherwise noted, all reagents were purchased from commercial suppliers and used as received.

### Note

Prior to the synthesis of all Pu-242 complexes, we performed numerous microscale syntheses with the U-238 surrogate, **1-U**, to ensure that we had experimental conditions that would reliably deliver single crystals suitable for single X-ray diffraction studies. The U-238 syntheses were performed in a positive-pressure non-transuranium UHP N_2_ atmosphere glovebox to optimize the synthetic methodology utilizing conventional non-aqueous starting materials. Additionally, it is crucial that, before carrying out the synthesis for all the Pu-242 complexes, the atmosphere of the transuranium glovebox is thoroughly purged with UHP N_2_ and tested with ketyl reagent before use to prevent unwanted decomposition products. All U-238 and Pu-242 complex yields were determined by weighing the mass of the isolated crystals in the inert transuranium-glovebox, in which all yields were finalized in triplicate.

### Caution

^242^Pu decays principally through α-emission (Qa = 4.984 MeV, 3.94 × 10^−3 ^Ci/g) with a half-life of 3.73 × 105 years. It is important to note that the ^242^Pu stock is 99.98% ^242^Pu by mass and is responsible for 84% of the α-particles emitted from the sample. A small amount of ^240^Pu (0.02% mass fraction, t_1/2_ = 6.561 × 103 years) and ^238^Pu (<0.01% mass fraction, t_1/2_ = 87.71 years) are responsible for 16% of the α-particles emitted from the sample. A much smaller amount of ^241^Pu (<<0.001% mass fraction, t_1/2_ = 14.329 years, β – 99.998%) does not contribute significantly to mass fraction or α-particle emission but instead results in a measurable amount of ^241^Am (t_1/2_ = 432.6 years), which must be separated before proceeding with synthetic efforts. All manipulations that involved ^242^Pu material were conducted in a specialized transuranium radiological designated area equipped with high-efficiency particulate air (HEPA) filtered hoods and negative-pressure gloveboxes. Multiple levels of containment were utilized when appropriate for safety reasons. Additional safety controls included continuous air monitoring for airborne α-emitting particles and the use of hand-held radiation monitoring equipment. Entrance to the laboratory space was controlled with a hand and foot radiation monitoring instrument, and the radiation dose exposure was further monitored by wearing whole-body and extremity dosimetry. Due to these radiological hazards, elemental analyses and other external analytical user services commonly obtained on uranium complexes were not possible on complexes containing ^242^Pu radioisotopes. Unless otherwise noted, all manipulations were performed under Ultra High Purity (UHP) N_2_ (AirGas) with rigorous exclusion of oxygen and moisture in a negative-pressure MBraun glovebox (with a −40 °C freezer) configured for the safe containment of transuranic isotopes. The glovebox atmosphere was maintained by passing the UHP N_2_ gas through a Cu/molecular sieve drying column and standalone Vacuum Atmosphere Genesis^TM^ oxygen and moisture removal systems. The suitability of the atmosphere was verified before use by placing a small drop of ketyl reagent onto a glass slide and monitoring the deep purple color.

Depleted uranium (primary isotope ^238^U) is a weak α-emitter (4.197 MeV) with a half-life of 4.47 × 109 years. Manipulations and reactions should be carried out in monitored fume hoods or in an inert glovebox in a radiation laboratory equipped with α- and β-counting equipment. Unless otherwise noted, all manipulations were performed under Ultra High Purity (UHP) N_2_ with rigorous exclusion of oxygen and water by Schlenk line and glovebox techniques.

### NMR

Spectra were obtained on a Bruker AVANCE NEO 300 MHz spectrometer and referenced to residual solvent resonances of toluene (*d*_*8*_-toluene). ^238^U NMR samples were prepared in Pyrex NMR tubes adapted with J-Young valves. ^242^Pu NMR samples were prepared by adding the appropriate solution into a FEP (fluorinated ethylene propylene) liner and sealing with a PTFE plug. The capped liner was decontaminated inside the transuranium glovebox, then loaded into a Pyrex NMR tube adapted with a J-Young valve, which was then removed from the glovebox, thoroughly decontaminated, and verified to be free of any surface contamination prior to obtaining all spectra.

### Single crystal X-ray diffraction

Data for the analyzed crystal structures were collected using Cu *K*_*α*_ radiation (λ = 1.54184 Å) on a Rigaku XtaLAB Synergy-S diffractometer fitted with a HyPix-6000HE detector, both operating at T = 240.01(10) K. ^242^Pu single crystals suitable for X-Ray diffraction analysis were mounted in Paratone-N oil and affixed with epoxy on MiTeGen MicroLoops inserted into MiTeGen Goniometer bases. The crystals were contained by covering the loop with a MiTeGen clear polyester MicroRT™ Capillary, in which the end was sealed with epoxy. The bases were capped to prevent bumping and removed from the glovebox for thorough decontamination, which were verified to be rid of surface contaminants prior to collecting data.

Single dark teal plate crystals of complex **1-Pu**, single dark purple plate crystals of complex **2-Pu**, single dark red plate crystals of complex **3-U**, single dark orange plate crystals of complex **4-U**, and single dark brown plate crystals of complex **5-Pu** were used. The following data reduction and correction were carried out by *CrysAlis*^*Pro*^^103^. The solutions and refinements were performed by *SHELXT* and *SHELXL*^104^, respectively, in Olex2^105^. The crystal structures were refined using full-matrix least-squares based on ***F***^**2**^ with all non-H atoms defined in an anisotropic manner. Hydrogen atoms were placed in calculated positions by means of the “riding” model. The refinement of the crystal structures needed some restraints dealing with atomic distances (SADI. DFIX, DANG cards) or anisotropic refinement (RIGU, SIMU instructions). This was due to the disorder displayed, mostly, by some ligands or solvent molecules. **CCDC numbers** are 2446212 (complex **1-Pu**), 2446213 (complex **2-Pu**), 2446214 (complex **3-U**), 2446215 (complex **4-U**), and 2446216 (complex **5-Pu**).

### Solid-state absorption spectra

Were collected at room temperature from 350 nm to 1700 nm on a CRAIC Technologies Microspectrophotometer with a 75 W xenon lamp. ^242^Pu and ^238^U crystals were mounted on a glass slide with a well in Paratone-N oil and covered with a thin glass cover slip and sealed with epoxy. The slides were then removed from the glovebox, thoroughly decontaminated, and verified to be free of radioactive contaminants prior to obtaining all spectra.

### Solution-state absorption spectra

Were collected on an Agilent Cary 5000 UV-Vis-NIR spectrometer. The solutions were contained in a low-volume (1 mL) screw-capped quartz cuvette (1 cm path length), and the data were collected from 350 to 1700 nm at room temperature.

### Elemental analyses

Were carried out on complexes **3-U** and **4-U** by Dr. Ashleigh Lightfoot at the UC Berkeley Microanalytical facility with a Perkin Elmer 2400 Series II combustion analyzer.

### Synthesis of KCp^Me4^^74^

A solution of HCp^Me4^ (0.98 g, 0.008 mmol) in 10 mL of toluene was added to a solution of KN(SiMe_3_)_2_ (1.955 g, 0.010 mmol, 1.25 equiv.) in toluene (10 mL). After stirring for 12 hours, the solution was filtered over a glass filter frit, and the insoluble yellow-white solids were washed with toluene until all the yellow contaminant was removed. The white KCp^Me4^ (1.183 g, 92 % yield) product was obtained after drying under dynamic vacuum for four hours.

### Modified synthesis of [Pu^III^I_3_(THF)_4_]^53^

A 20 mL vial equipped with a Teflon-coated magnetic stir bar was charged with [Pu^IV^CI_4_(DME)_2_] (26.0 mg, 0.0462 mmol, 1.0 equiv.) and suspended in 1.5 mL of Et_2_O. Neat Me_3_SiI (50.equiv. with respect to Pu) was added to the stirred suspension of [Pu^IV^CI_4_(DME)_2_] at room temperature, resulting in a yellow-brown residue and a pink supernatant. After 30 mins of stirring, 5 mL of *n*-hexanes was added, and the mixture was placed in the glovebox freezer and allowed to settle at −40 °C for 1 hour. The red supernatant was removed. Fresh Et_2_O (0.15 mL) was added to the yellow-brown solids and stirred for 2 minutes. Then, 2.0 mL of n-hexane was added to the suspension and stirred again for another 2 minutes. The solids were allowed to settle at −40 °C, and the supernatant was removed again. The Et_2_O/*n*-hexanes washes were repeated until the supernatant became colorless. The yellow-brown solids were dried under vacuum (2.3 mbar, diaphragm pump) for 1 hour to remove any traces of I_2_. Minimal THF (0.5 mL) was added to the dried pale green powder, which caused an instantaneous color change to pale grey-purple, and some microcrystalline purple material rapidly precipitated from the THF. The vial was capped and carefully heated (90 °C) to ensure all material fully contacted with the THF. The mixture was then allowed to cool to room temperature, and the mixture was placed at −40 °C overnight to precipitate more product. The next day, there was the formation of large pale purple crystals in addition to the microcrystalline solids. The solids were collected by removing the supernatant and drying under vacuum (2.3 mbar, diaphragm pump) for 1 hour, yielding [Pu^III^I_3_(THF)_4_] (36.0 mg, 74 % yield).

### Synthesis of [Pu^III^(Cp^Me4^)_3_] (1-Pu)

A 6 mL vial equipped with a Teflon-coated magnetic stirbar was charged with [Pu^III^I_3_(THF)_4_] (11.0 mg, 0.0121 mmol, 1.0 equiv.). A separate 6 mL vial was charged with KCp^Me4^ (6.8 mg, 0.0424 mmol, 3.5 equiv.) and suspended in 0.6 mL of toluene. The suspension of KCp^Me4^ was added to the vial containing complex [Pu^III^I_3_(THF)_4_].; Upon addition, the solution turned bright teal-green in color with concomitant formation of an off-white solid (mixture of KI and excess KCp^Me4^) (Supplementary Fig. 1b). After 16 hours of stirring at room temperature, the resulting teal-green mixture was transferred to a 2 mL plastic centrifuge tube, the reaction mixture vial was washed with toluene and transferred (0.1 mL x 2), and the off-white solids were separated by centrifugation from the teal-green supernatant. The teal-green supernatant was transferred to a new vial, in which the centrifuge tube was washed with toluene (0.1 mL x 2) (Supplementary Fig. 1c). All the volatiles were removed under vacuum (2.3 mbar, diaphragm pump) with moderate heating (50 °C), resulting in a dark teal microcrystalline solid (Supplementary Fig. 1d). The solids were suspended in 0.4 mL of Et_2_O and brought into solution by carefully heating the closed vial on a hotplate set at 70 °C. Once mostly dissolved, the mixture was removed from heat and allowed to cool to room temperature, at which point teal-green crystals began to immediately crash out. Once cooled, the mixture was placed at −40 °C overnight, yielding teal-green single crystals of complex **1-Pu** suitable for XRD analysis (6.3 mg, 85% yield; Supplementary Fig. 1e, f). Complex **1-Pu** is stable in toluene solutions and in the solid state at -40 °C and room temperature for up to 2 months, in which the stability was monitored by ^1^H NMR spectroscopy (Supplementary Fig. 7). ^**1**^**H NMR** (400 MHz, *d*_*8*_-toluene, 298 K): δ 24.1 (3H, broad s), 0.76 (18H, s), -0.09 (18H, s) ppm (Supplementary Fig. 6).

### Note

On one occasion, after the teal-green solution was obtained after centrifugation for the synthesis of **1-Pu**, the volatiles were removed by blowing down the solution under a stream of the glovebox atmosphere (*ca*. 1.0 ppm of H_2_O). This led to the isolation of a hydrolysis product, [{Pu^III^(Cp^Me4^)_2_}_2_(*μ*-OH)_2_] (**5-Pu**; Supplementary Fig. 22), presumably due to adventitious H_2_O in the glovebox atmosphere. Once **5-Pu** product is isolated as crystals from a concentrated toluene solution at −40 °C (Supplementary Fig. 2), it becomes completely insoluble in toluene. Attempts to redissolve **5-Pu** in toluene and Et_2_O with slight heating proved unsuccessful due to the extreme insolubility. Due to this insolubility and the small amount of material, we were unable to collect solution-state ^1^H NMR and UV-Vis-NIR spectroscopy data for this sample. However, we were successful in collecting solid-state absorption UV-Vis-NIR data from the crystals obtained (Supplementary Fig. 35).

### Synthesis of [U^III^(Cp^Me4^)_3_] (1-U)^74,82^

A 20 mL vial equipped with a Teflon-coated magnetic stir bar was charged with [U^III^I_3_(THF)_4_] (135.7 mg, 0.15 mmol, 1.0 equiv.). A separate 6 mL vial was charged with KCp^Me4^ (84.2 mg, 0.525 mmol, 3.5 equiv.) and suspended in 3.0 mL of toluene. The suspension of KCp^Me4^ was added to the vial containing complex [U^III^I_3_(THF)_4_], whereupon addition, the solution turned green in color with concomitant formation of an off-white solid (mixture of KI and excess KCp^Me4^). After 16 hours of stirring at room temperature, the resulting white solids were separated from the green-brown supernatant by centrifugation. All the volatiles were removed under high vacuum, resulting in a dark green-brown microcrystalline solid (82.3 mg, 90 % yield).

### Synthesis of [Sm^III^(Cp^Me4^)_3_] (1-Sm)^75^

A 20 mL vial equipped with a Teflon-coated magnetic stir bar was charged with [Sm^III^I_3_(THF)_4_] (32.8 mg, 0.04 mmol, 1.0 equiv.). A separate 6 mL vial was charged with KCp^Me4^ (22.4 mg, 0.14 mmol, 3.5 equiv.) and suspended in 1.0 mL of toluene. The suspension of KCp^Me4^ was added to the vial containing complex [Sm^III^I_3_(THF)_4_], where upon addition, the solution turned bright orange in color with concomitant formation of an off-white solid (mixture of KI and excess KCp^Me4^). After 16 hours of stirring at room temperature, the resulting white solids were separated from the orange supernatant by centrifugation. All the volatiles were removed under high vacuum, resulting in a bright orange microcrystalline solid (19.5 mg, 95 % yield).

### Synthesis of [{Pu^III^(Cp^Me4^)_2_}_2_(*μ*−SPh)_2_] (2-Pu)

A 6 mL Schlenk tube equipped with a Teflon-coated stir bar was charged with complex **1-Pu** (5.5 mg, 0.009 mmol, 1.0 equiv.) and dissolved in 0.2 mL of toluene. From a separate vial, 50.0 μL of a (PhS)_2_ stock solution (0.5 equiv.) was added into the Schlenk tube containing complex **1-Pu**, resulting in no obvious color change. The flask was then connected to the vacuum line inside the glovebox, and the toluene and N_2_ headspace was removed. This was then connected to an Ar line inside the glovebox, where 0.3 mL of toluene was added under a stream of Ar. The vessel was then sealed with a J-Young valve to minimize exposure to the glovebox atmosphere (***note*:** due to the long reaction time, and dependent on the quality of the glovebox atmosphere (0.5-0.8 ppm of H_2_O and O_2_), stirring the reaction mixture in a vial can lead to higher yields of the H_2_O decomposition by-product (**5-Pu**), therefore; stirring under an Ar atmosphere in a highly-sealed vessel is encouraged). The reaction mixture over the course of 7 days changed color between teal-green, dark blue (1–3 days), blue-purple (3–5 days), and purple (6–7 days) (Supplementary Fig. 3a–d). Once the dark purple color was obtained, all the volatiles were removed under vacuum (2.3 mbar, diaphragm pump), resulting in a dark purple microcrystalline solid. The solids were suspended in 0.2 mL of hexane and warmed into solution by carefully heating the closed vial on a hotplate set at 70 °C. Once mostly soluble, the solution was removed from heat and allowed to cool to room temperature, at which purple crystals began to immediately crash out. Once cooled, the mixture was placed at −40 °C overnight, yielding purple single crystals of complex **2-Pu** suitable for XRD analysis (70% yield) (Supplementary Fig. 3e), after removal of a pale yellow supernatant. The supernatant was confirmed to contain (Cp^Me4^)_2_ and residual (PhS)_2_ by ^1^H NMR spectroscopy (Supplementary Fig. 12c). Complex **2-Pu** is stable in Ar-sparged toluene solutions and the solid state at -40 °C and room temperature for up to two weeks. ^**1**^**H NMR** (400 MHz, *d*_*8*_-toluene, 298 K): δ 7.9 (4H, broad s), 7.3 (2H, m), 5.2 (8H, broad s), 1.4 (48H, s) ppm (Supplementary Fig. 8).

### Synthesis of [U^IV^(Cp^Me4^)_3_(SPh)] (3-U)

A 6 mL vial was charged with complex **1-U** (24.4 mg, 0.040 mmol, 1.0 equiv.) and dissolved in 1.0 mL of toluene. A separate vial was charged with (PhS)_2_ (4.4 mg, 0.020 mmol, 0.5 equiv.) and dissolved in 0.4 mL of toluene, then added to the vial containing complex **1-U**, resulting in an immediate color change from brown-green to dark brown-orange. The mixture was allowed to react for 5 minutes, in which all the volatiles were removed under vacuum, resulting in a dark orange residue. The solids were suspended in 0.5 mL of Et_2_O and warmed into solution by carefully heating the closed vial on a hotplate set at 70 °C, causing dissolution after 5 minutes. Once soluble, the solution was removed from heat and allowed to cool to room temperature, at which point dark brown-orange crystals began to crash out. Once cooled, the mixture was placed at -40 °C overnight, yielding dark brown-orange single crystals of complex **3-U** suitable for XRD analysis (22.7 mg, 81% yield). Complex **3-U** is stable in solution (toluene) and the solid state at −40 °C and room temperature for up to one month. ^**1**^**H NMR** (400 MHz, *d*_*8*_-toluene, 298 K): δ 34.9 (9H, broad s), 13.3 (18H, broad s), −2.4 (2H, m), −2.9 (1H, m), −12.2 (3H, s), −20.4 (2H, s), −33.6 (9H, broad s) ppm (Supplementary Fig. 9). *Anal. Calcd*. for: C_33_H_44_SU, C, 55.76; H, 6.24. *Found:* C, 55.62; H, 6.18.

### Synthesis of [U^IV^(Cp^Me4^)_3_(NHPh)] (4-U)

A 6 mL vial was charged with complex **1-U** (24.4 mg, 0.040 mmol, 1.0 equiv.) and dissolved in 1.0 mL of toluene equipped with a glass-coated stir bar. A separate vial was charged with (PhHN)_2_ (3.6 mg, 0.020 mmol, 0.5 equiv.) and dissolved in 0.4 mL of toluene, then added to the vial containing complex **1-U**, resulting in no obvious color change. The mixture was allowed to stir at room temperature for 4 days, during which the reaction mixture changed color between brown-green, brown-red, and dark red. Once the dark red color was obtained, all the volatiles were removed under vacuum, resulting in a dark red residue. The solids were suspended in 0.5 mL of n-hexanes and warmed into solution by carefully heating the closed vial on a hotplate set at 70 °C, causing dissolution after 5 minutes. Once soluble, the solution was removed from heat and allowed to cool to room temperature, then the mixture was placed at −40 °C overnight, yielding dark red single crystals of complex **4-U** suitable for XRD analysis (24.3 mg, 88% yield). Complex **4-U** is stable in solution (toluene) and the solid state at −40 °C and room temperature for up to one month. ^**1**^**H NMR** (400 MHz, *d*_*8*_-toluene, 298 K): δ 16.2 (24H, broad s), −1.8 (2H, t), −4.3 (1H, t), −8.1 (12H, broad s), −15.5 (3H, s), −30.6 (2H, d), −196.6 (1H, s) ppm (Supplementary Fig. 10). *Anal. Calcd*. for: C_33_H_45_NU, C, 57.13; H, 6.54; N, 2.02. *Found:* C, 57.22; H, 6.48; N, 1.98.

### NMR-scale reaction of 1-Pu with 0.5 equiv. (PhS)_2_

A FEP liner was charged with complex **1-Pu** (3.0 mg, 0.005 mmol) dissolved in 0.2 mL of *d*_*8*_-toluene. From a separate vial, 50.0 μL of a (PhS)_2_ solution (0.5 equiv.) in *d*_*8*_-toluene was added to the FEP liner containing **1-Pu**, resulting in no obvious color change. The FEP liner was capped with a PTFE plug and transferred into an NMR tube equipped with a J-Young valve and analyzed by ^1^H NMR spectroscopy (Supplementary Fig. 11) over 7 days with a color change from teal-green to dark purple, resulting in the formation of complex **2-Pu** and (Cp^Me4^)_2_^1^. We note that the control experiment, where (PhS)_2_ and KCp^Me4^ react over seven days, as monitored by ^1^H NMR spectroscopy, yields a mixture of PhS-Cp^Me4^, unidentifiable material, and no (Cp^Me4^)_2_, indicating that complex **1-Pu** is required for reductive cleavage reactivity (Supplementary Fig. S17).

### NMR-scale reaction of 1-Pu with 0.5 equiv. (PhHN)_2_

A FEP liner was charged with complex **1-Pu** (3.0 mg, 0.005 mmol) dissolved in 0.2 mL of d_8_-toluene. From a separate vial, 40.0 μL of a (PhHN)_2_ solution (0.5 equiv.) in d_8_-toluene was added to the FEP liner containing **1-Pu**, resulting in no obvious color change. The FEP liner was capped with a PTFE plug and transferred into an NMR tube equipped with a J-Young valve and analyzed by ^1^H NMR spectroscopy over 14 days, resulting in no consumption of complex **1-Pu**. The only reactivity observed is the decomposition of the (PhHN)_2_ starting material (Supplementary Fig. 13) to (PhN)_2_ (31% yield) and unidentified species. We propose tthat his decomposition occurs due to the radiolysis of Pu because when the control reaction of (PhHN)_2_ in d_8_-toluene is carried out, there is no appreciable decomposition over 14 days at room temperature.

### NMR-scale reaction of 1-Sm with 0.5 equiv. (PhS)_2_

An NMR tube equipped with a J-Young valve was charged with complex **1-Sm** (0.010 mmol) dissolved in 0.4 mL of d_8_-toluene. From a separate vial, 50.0 μL of a (PhS)_2_ solution (0.5 equiv.) in d_8_-toluene was added to the J-Young tube containing complex **1-Sm**, resulting in no obvious color change. The mixture was analyzed by ^1^H NMR spectroscopy (Supplementary Fig. 14), resulting in no consumption of **1-Sm** or (PhS)_2_. The reaction mixture was monitored over the course of 5 days, during which the spectrum remained unchanged.

### NMR-scale reaction of 1-U with 0.5 equiv. (PhS)_2_

An NMR tube equipped with a J-Young valve was charged with complex **1-U** (6.1 mg, 0.010 mmol) dissolved in 0.4 mL of d_8_-toluene. From a separate vial, 40.0 μL of a (PhS)_2_ solution (0.5 equiv.) in d_8_-toluene was added to the J-Young tube containing complex **1-U**, resulting in an immediate color change from brown-green to dark red. The mixture was analyzed by ^1^H NMR spectroscopy (Supplementary Fig. 15), resulting in the formation of complex **3-U** and unknown resonances. The reaction mixture was monitored over the course of 5 days, during which the spectrum was unchanged, and the unknown signals most likely correspond to an undetermined impurity.

### NMR-scale reaction of 1-U with 0.5 equiv. (PhHN)_2_

An NMR tube equipped with a J-Young valve was charged with complex [U^III^(Cp^Me4^)_3_] (6.1 mg, 0.010 mmol) and dissolved in 0.4 mL of d_8_-toluene. From a separate vial, 40.0 μL of a (PhHN)_2_ solution (0.5 equiv.) in d_8_-toluene was added to the J-Young tube containing complex **1-U**, resulting in no obvious color change. The mixture was analyzed by ^1^H NMR spectroscopy (Supplementary Fig. 16) over 5 days, resulting in the gradual formation of complex **4-U** and color changes from brown-green to dark orange.

### NMR-scale reaction of 3-U with 1.0 equiv. (PhS)_2_

An NMR tube equipped with a J-Young valve was charged with complex **3-U** (4.7 mg, 0.0066 mmol) dissolved in 0.4 mL of d_8_-toluene. From a separate vial, 40.0 μL of a (PhS)_2_ solution (1.0 equiv.) in d_8_-toluene was added to the J-Young tube containing complex **3-U**, resulting in no color change from dark red. The mixture was analyzed by ^1^H NMR spectroscopy over 5 days, resulting in unreacted starting materials.

### NMR-scale reaction of 4-U with 1.0 equiv. (PhHN)_2_

An NMR tube equipped with a J-Young valve was charged with complex **4-U** (4.7 mg, 0.0068 mmol) and dissolved in 0.4 mL of d_8_-toluene. From a separate vial, 40.0 μL of a (PhHN)_2_ solution (1.0 equiv.) in d_8_-toluene was added to the J-Young tube containing complex **4-U**, resulting in no obvious color change. The mixture was analyzed by ^1^H NMR spectroscopy over 5 days, resulting in unreacted starting materials.

## Supplementary information

Supplementary Information

Transparent Peer Review file

## Source data

Source Data

## Data Availability

Crystallographic data for the structures reported in this study have been deposited at the Cambridge Crystallographic Data Centre, under deposition numbers CCDC 2446212 (**1-Pu**), 2446213 (**2-Pu**), 2446214 (**3-U**), 2446215 (**4-U**), and 2446216 (**5-Pu**). These data can be obtained free of charge via https://www.ccdc.cam.ac.uk/structures/. The ^1^H NMR spectra, supplementary XRD data, and computational studies in this study are provided in the Supplementary Information file. Source data are provided with this manuscript. The computational XYZ coordinates and open data are available in the Mendeley database under accession code 29z869rwhb. All data are available from the corresponding author upon request. Source data are provided with this paper.
