# Supplementary material for: Plutonium(III) versus uranium(III) and samarium(III) in small molecule activation chemistry
Source: Nat Commun. 2026 May 2;17:5956. doi: 10.1038/s41467-026-72229-7 (PMC13342291; doi:10.1038/s41467-026-72229-7)
Supplement: Supplementary file 1 — Supplementary Information [file 41467_2026_72229_MOESM1_ESM.pdf]

Plutonium(III) versus uranium(III) and samarium(III) in small molecule activation chemistry

Supplementary Information

Megan Keener,[a] Thayalan Rajeshkumar,[b] Cambell Conour,[a] Joshua Woods,[a] Laurent Maron,[b] and Polly Arnold*[a]

[a]Chemical Sciences Division, Lawrence Berkeley National Laboratory, Berkeley and Department of Chemistry, University of California, Berkeley, CA, 94720, USA
[b]Laboratoire de Physique et Chimie des Nano-objets, Institut National des Sciences Appliquées, 31077 Toulouse, Cedex 4, France.

*Corresponding author: pla@berkeley.edu

Table of Contents

S1. Photographs taken during synthesis ... 2

S2. NMR Spectroscopy Data ... 7

S2.1 NMR spectra for isolated plutonium and uranium complexes ... 7

S2.2 NMR spectra for the reactions of plutonium and uranium complexes ... 12

S3. Supplementary X-Ray Crystallography Data ... 19

S4. Solution and solid-state absorption spectra ... 25

S5. Computational details ... 38

S5.1. Computational TDDFT details for complex, $[Pu^{III}(Cp^{Me4})_3]$ 1-Pu. ... 38

S5.2. Computational TDDFT details for complex, $[Pu^{III}(Cp^{Me4})_2(SPh)_2]$ 2-Pu. ... 40

S5.3. Computational TDDFT details for complex, $[U^{III}(Cp^{Me4})_3(SPh)]$ 3-U. ... 43

S5.4. Computational TDDFT details for complex, $[U^{III}(Cp^{Me4})_3(NHPh)]$ 4-U. ... 49

S5.5. Supplementary computed reaction profiles ... 54

S6. References ... 55

S1. Photographs taken during synthesis

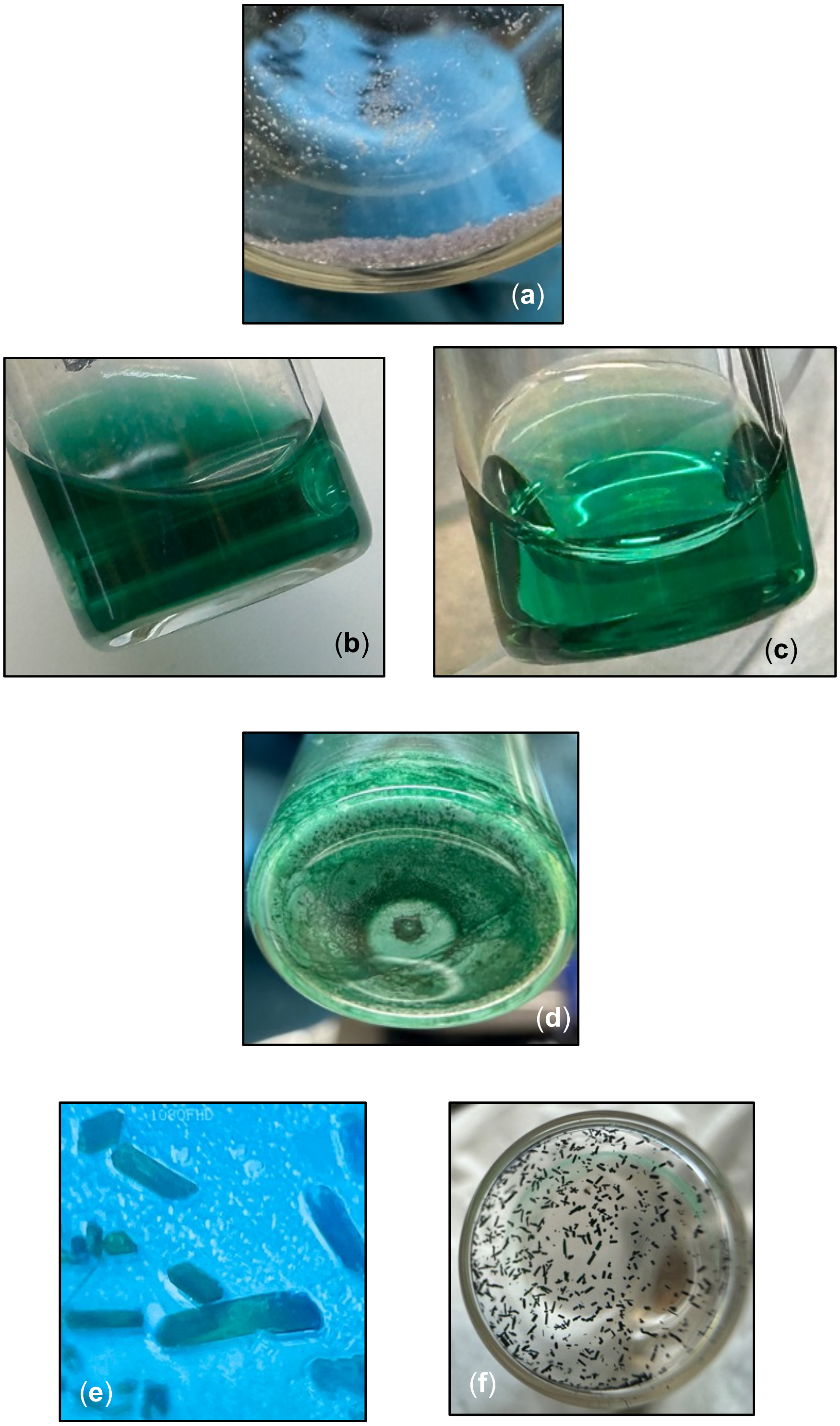

Supplementary Figure 1. Photographs from the synthesis of **1-Pu.** (**a**) Initial [$Pu^{III}I_3(THF)_4$] material before addition of KCp^{Me4}. (**b**) Pu-242 reaction mixture several min after addition of KCp^{Me4}. (**c**) Pu-242 solution after centrifugation and removal of KI and excess KCp^{Me4}. (**d**) Putative **1-Pu**, after removal of the volatiles under vacuum. (**e**) and (**f**) teal-green crystals of **1-Pu**. The vials are standard 4ml size. The size of the crystals (microscope images) are defined in the cif files.

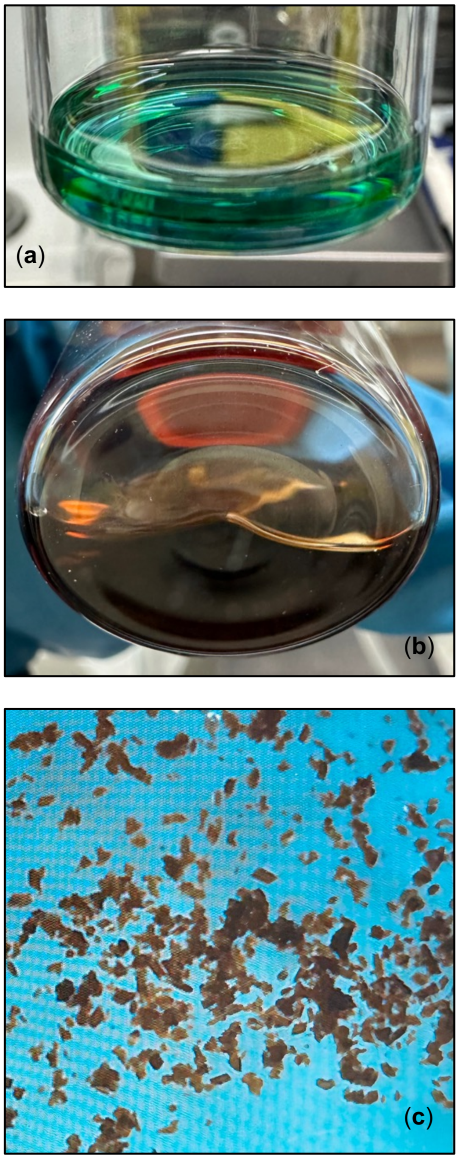

Supplementary Figure 2. Photographs from the synthesis of **5-Pu.** (**a**) Initial solution of **1-Pu** before blowing down the volatiles under a stream of the glovebox atmosphere. (**b**) Reaction mixture after blowing down *ca*. ½ of the solution volume yielding a dark brown solution. (**c**) Brown crystals of **5-Pu**. The vials are standard 4ml size. The size of the crystals (microscope images) are defined in the cif files.

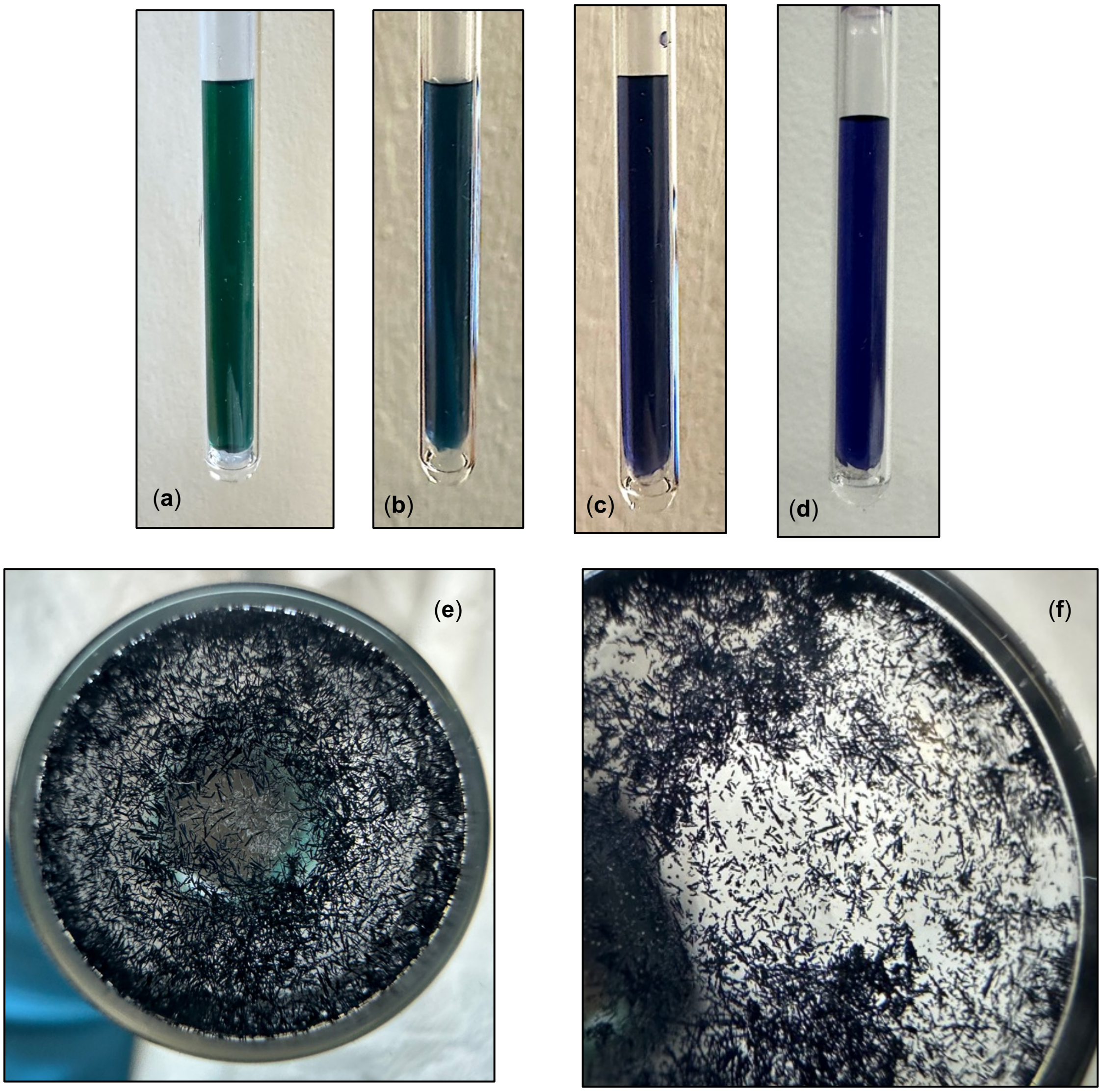

Supplementary Figure 3. Photographs from the synthesis of **2-Pu**. (**a**) Solution of **1-Pu** right after addition of $(PhS)_2$, resulting in no color change. (**b)** to **(d**) Reaction mixture of **1-Pu** and 0.5 equiv. of $(PhS)_2$ over 7 days. (**e**) Dark purple microcrystalline solids and (f) dark purple crystals of **2-Pu**. The vials are standard 4ml size and NMR tubes are 5mm diameter. The size of the crystals (microscope images) are defined in the cif files.

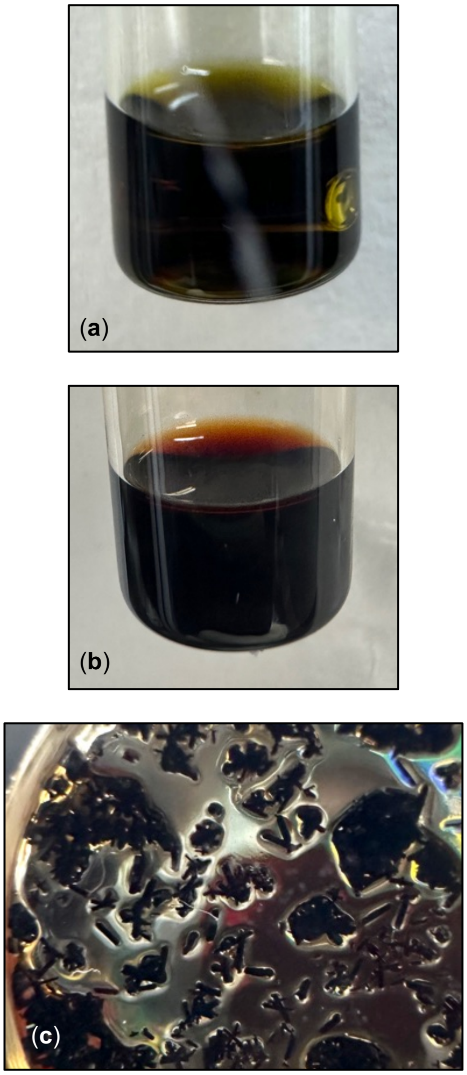

Supplementary Figure 4. Photographs from the synthesis of **3-U**. (**a**) Initial solution of complex **1-U** before addition of $(PhS)_2$. (**b**) Reaction mixture of **1-U** and 0.5 equiv. of $(PhS)_2$ immediately after addition. (**c**) Dark brown-orange crystals of **3-U**. The vials are standard 4ml size. The size of the crystals (microscope images) are defined in the cif files.

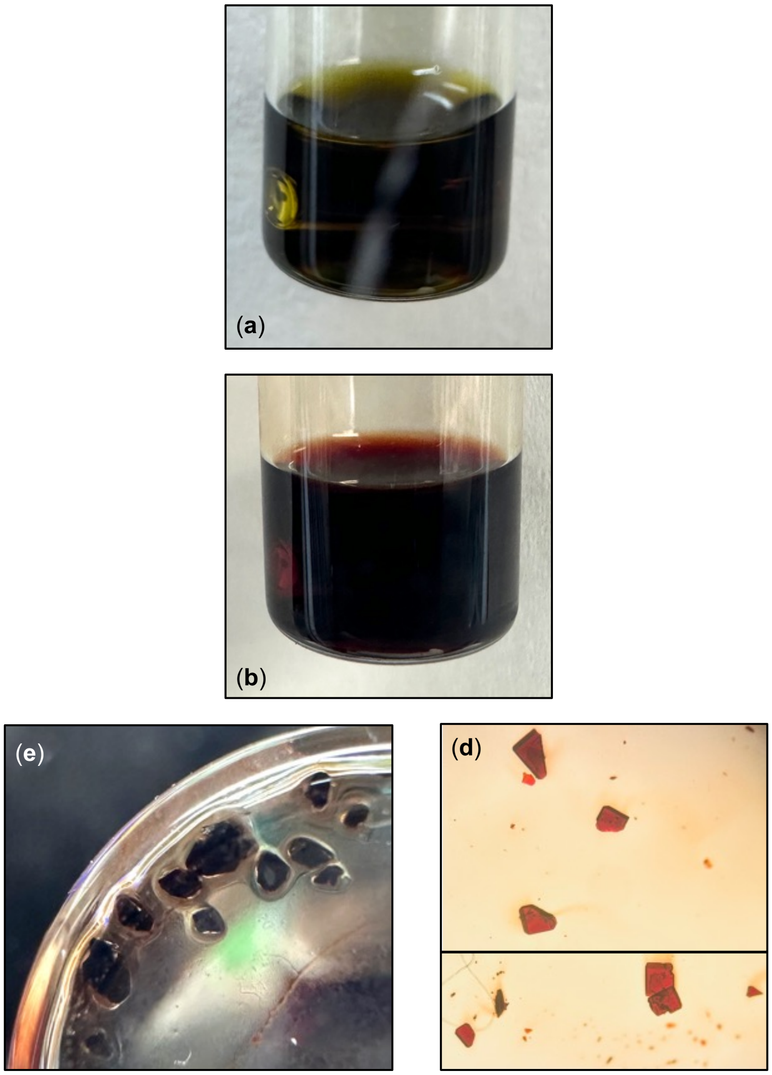

Supplementary Figure 5. Photographs from the synthesis of **4-U.** (**a**) Initial solution of complex **1-U** before addition of $(PhHN)_2$. (**b**) Reaction mixture of **1-U** and 0.5 equiv. of $(PhHN)_2$ after 4 days. (**c**, **d**) Dark red single crystals of **4-U**. The vials are standard 4ml size. The size of the crystals (microscope images) are defined in the cif files.

S2. NMR Spectroscopy Data

S2.1 NMR spectra for isolated plutonium and uranium complexes

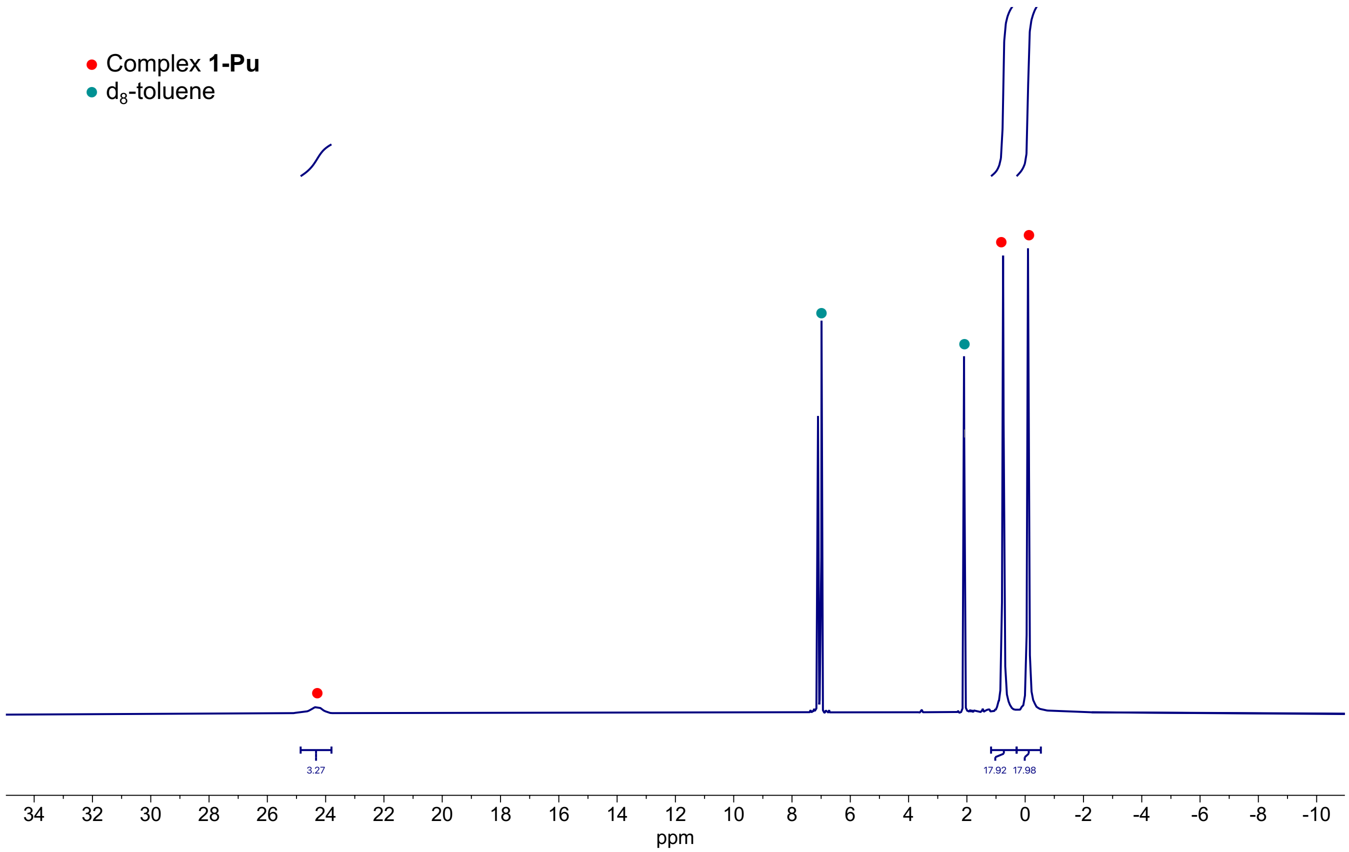

Supplementary Figure 6. ^{1}H NMR (400 MHz, d_8-toluene, 298K) spectrum of $[Pu^{III}(Cp^{Me4})_3]$, **1-Pu**. The red dots correspond to the assignments for **1-Pu** and the teal correspond to d_8-toluene solvent.

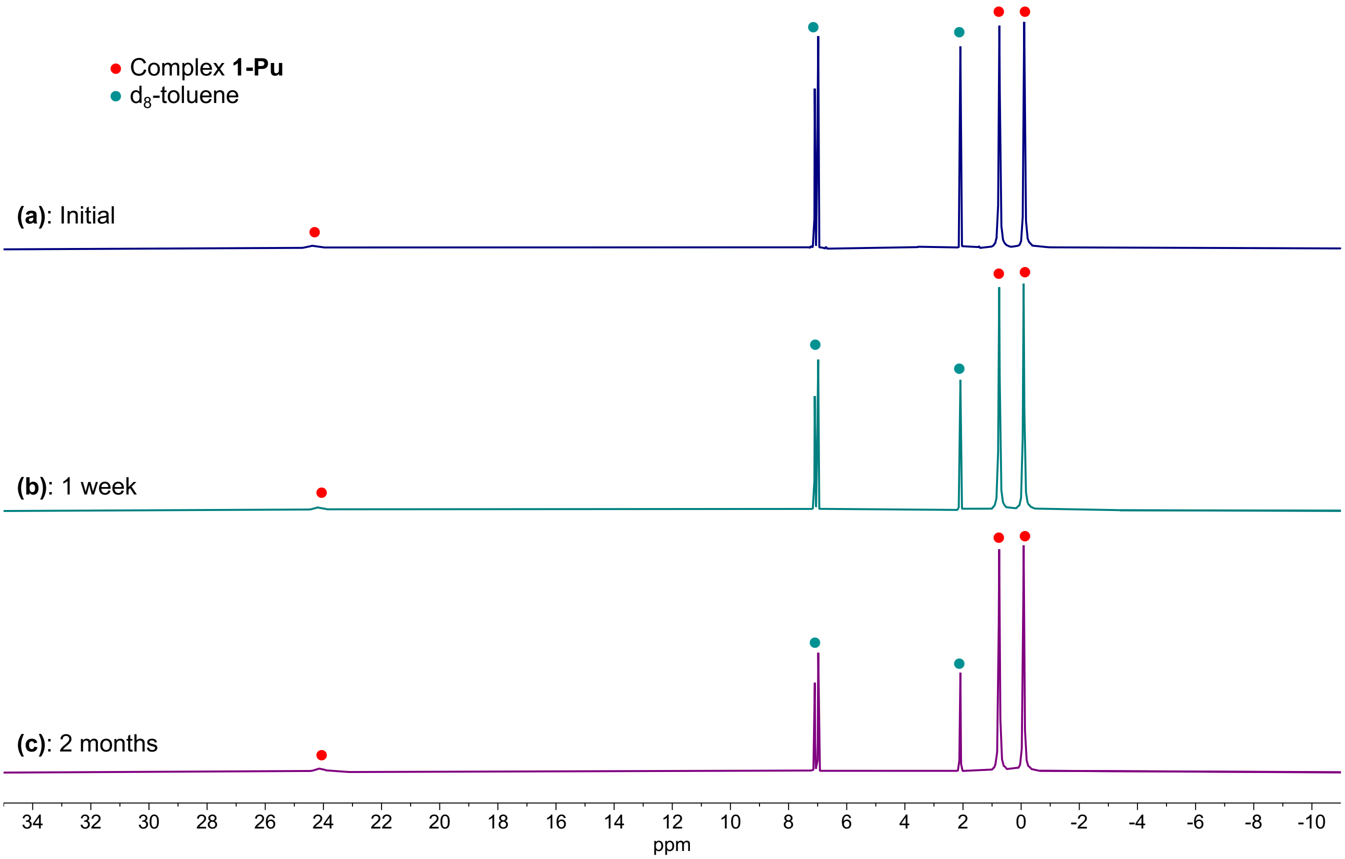

Supplementary Figure 7. ^{1}H NMR (400 MHz, d_8-toluene, 298K) spectrum of [Pu^{III}(Cp^{Me4})$_3$], **1-Pu** over 2 months. (**a**) Initial ^{1}H NMR spectrum of **1-Pu**. (**b**) After 1 week. (**c**) After 2 months.

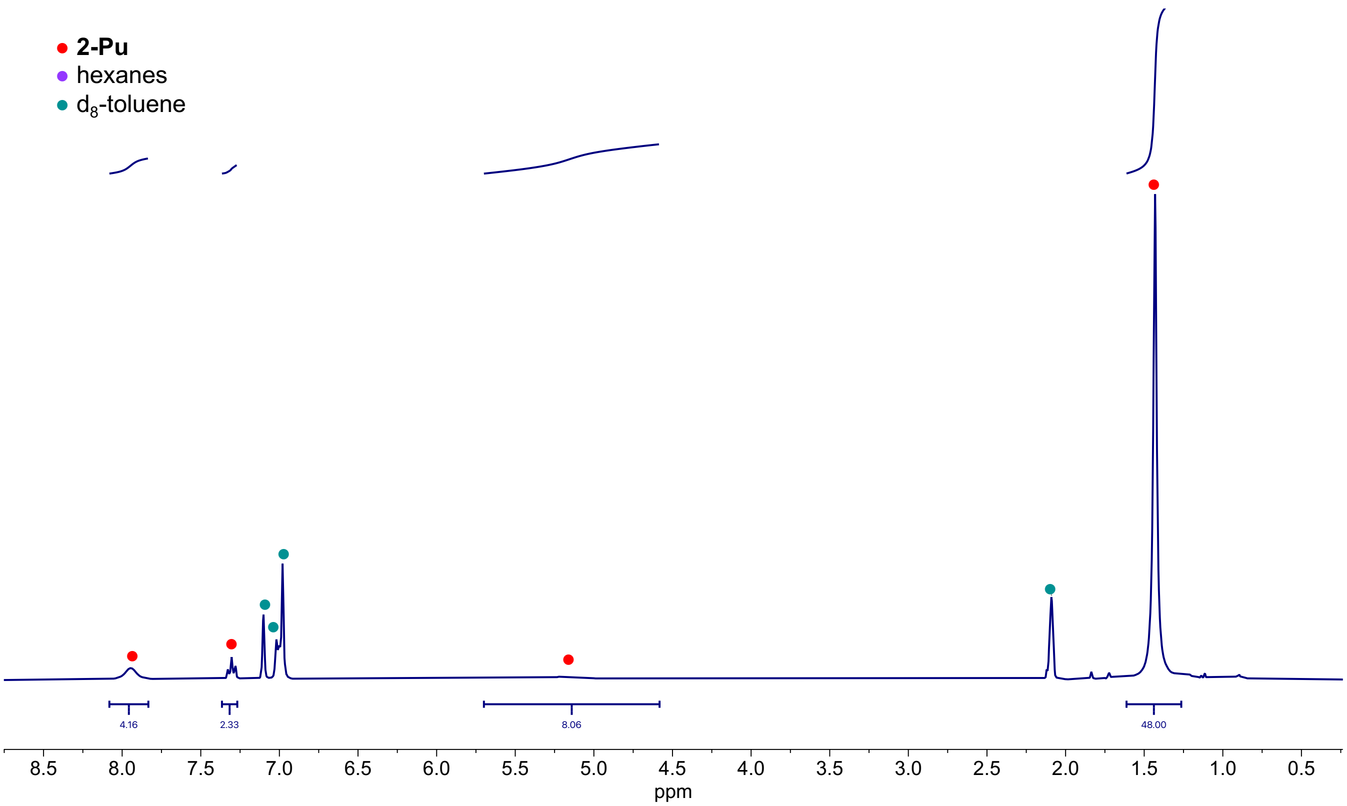

Supplementary Figure 8. ^{1}H NMR (400 MHz, d_8-toluene, 298K) spectrum of [$\{Pu^{III}(Cp^{Me4})_2\}_2(\mu\text{-}SPh)_2$], **2-Pu**. The red dots correspond to the assignments for **2-Pu** and the teal correspond to d_8-toluene solvent.

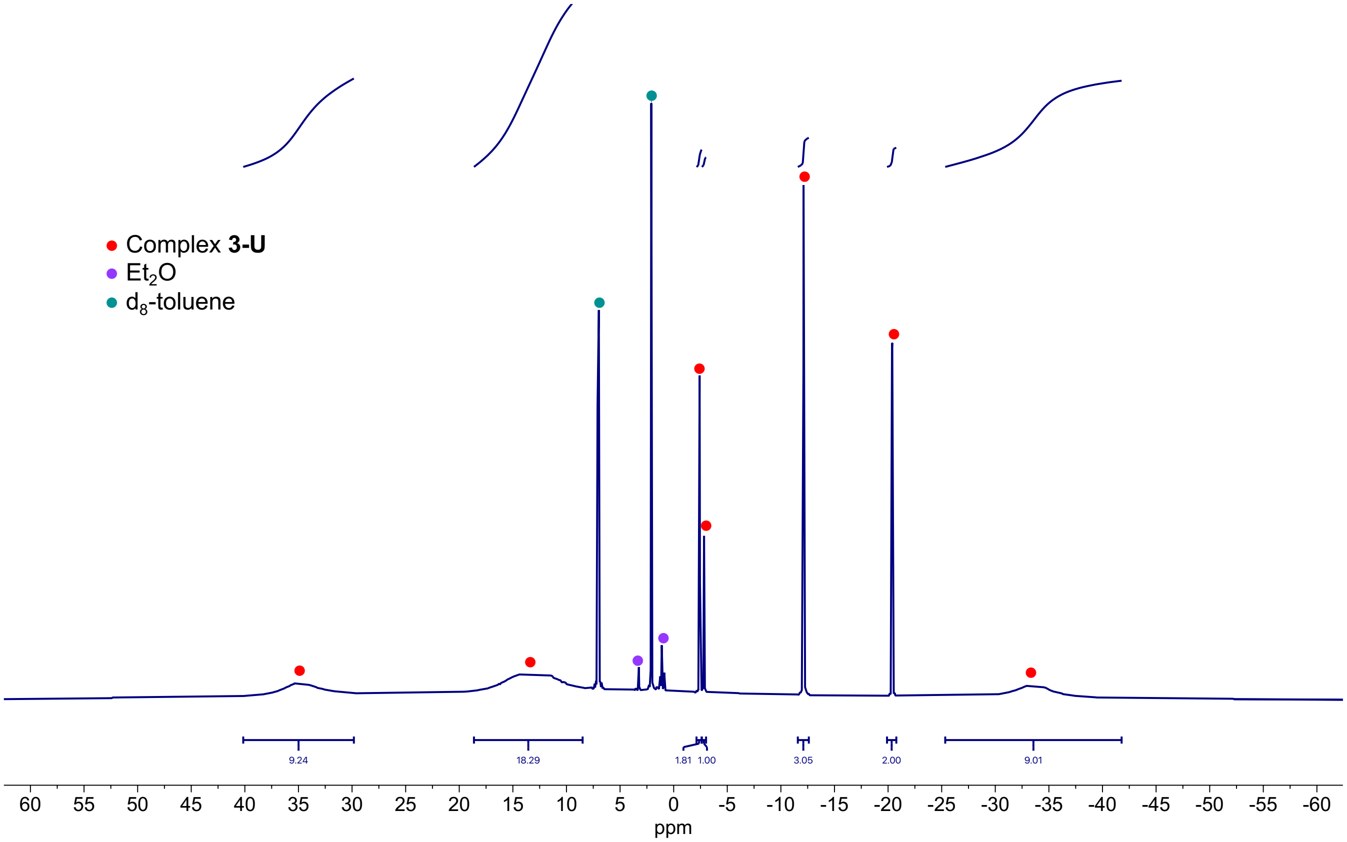

Supplementary Figure 9. ^{1}H NMR (400 MHz, d_8-toluene, 298K) spectrum of [U^{IV}(Cp^{Me4})$_3$(SPh)], **3-U**. The red dots correspond to the assignments for **3-U**, the teal correspond to d_8-toluene solvent, and purple are for residual Et$_2$O.

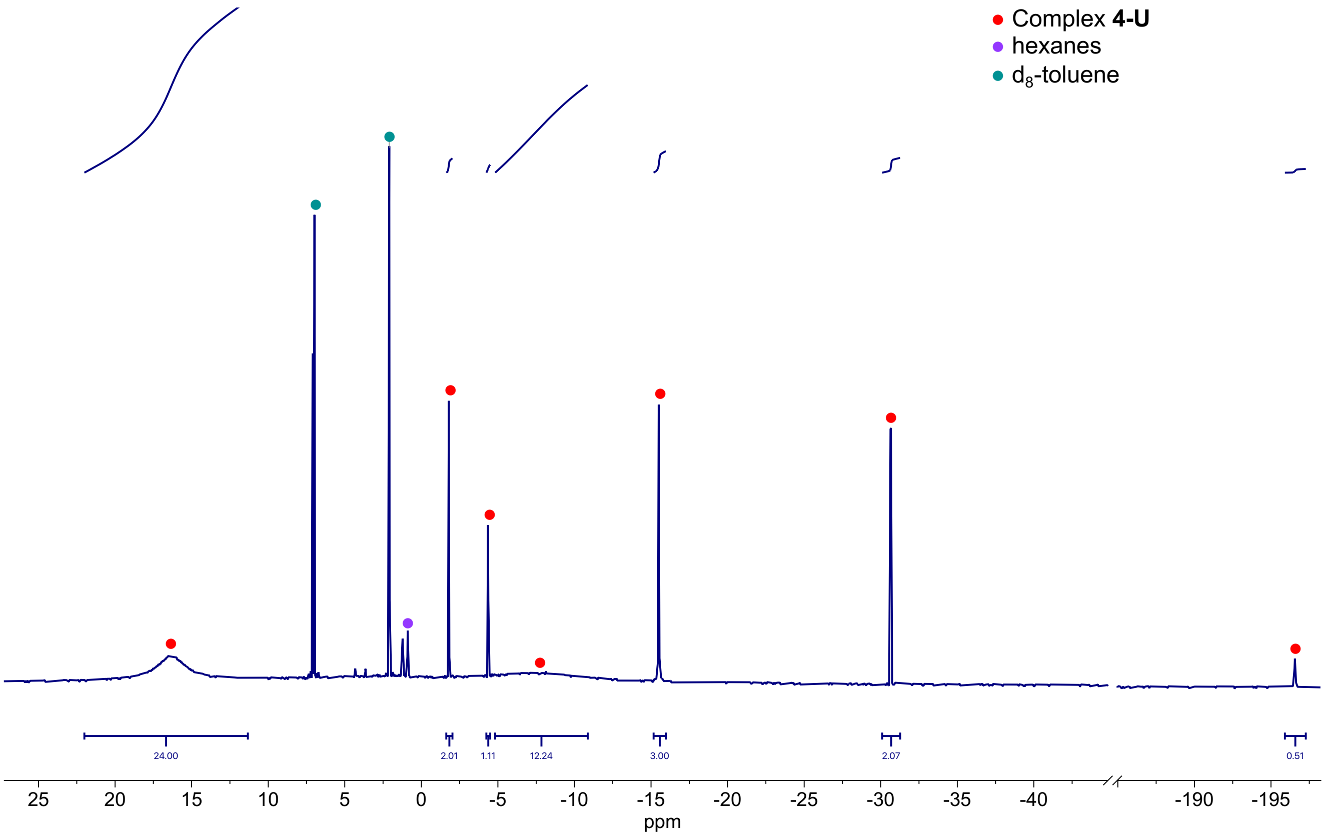

Supplementary Figure 10. ^{1}H NMR (400 MHz, *d$_8$*-toluene, 298K) spectrum of [U^{IV}(Cp^{Me4})$_3$(NHPh)], **4-U**. The red dots correspond to the assignments for **4-U**, the teal correspond to *d$_8$*-toluene solvent, and purple to residual *n*-hexanes.

S2.2 NMR spectra for the reactions of plutonium and uranium complexes

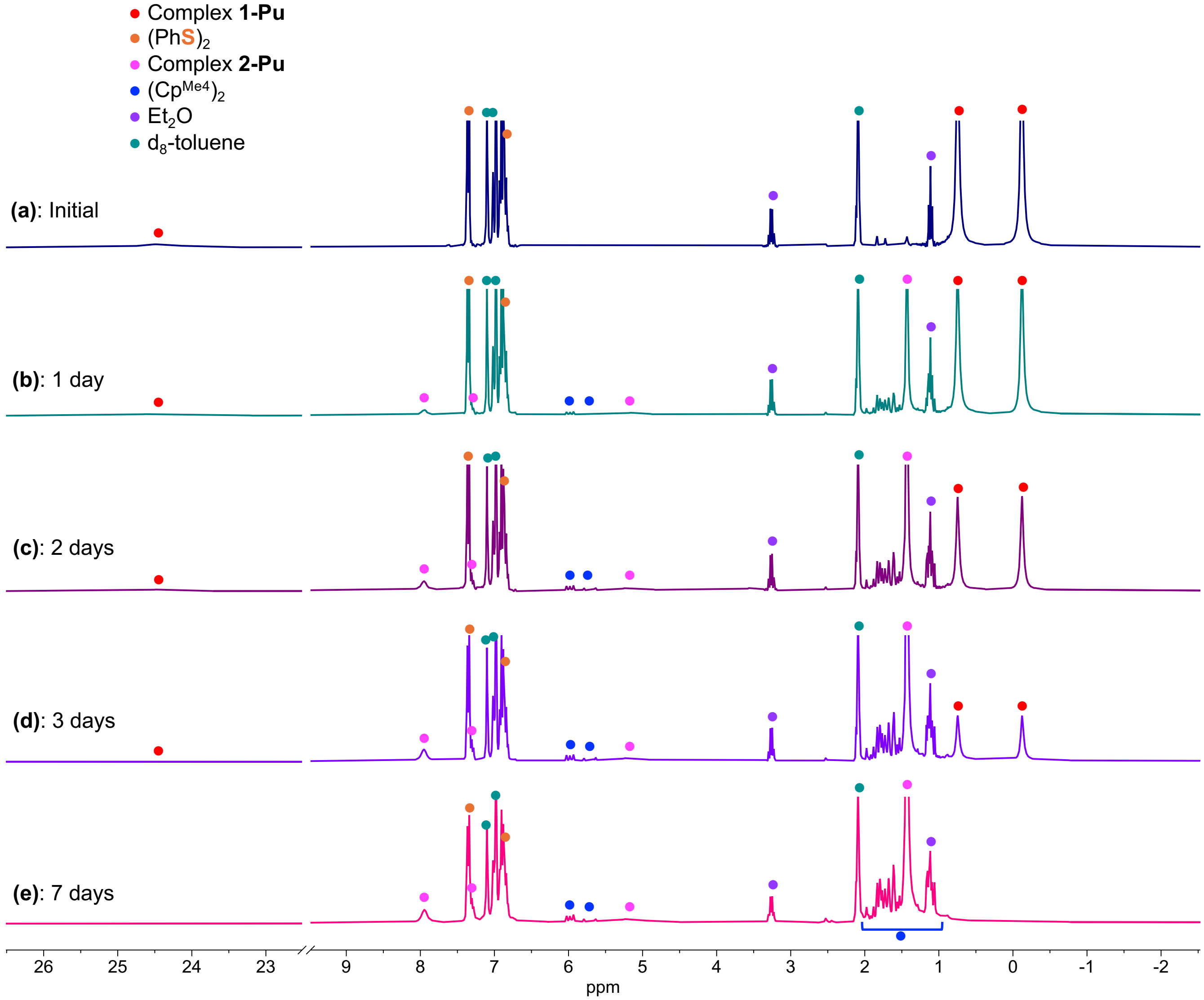

Supplementary Figure 11. ^{1}H NMR (400 MHz, *d₈*-toluene, 298K) spectra from reaction monitoring of complex **1-Pu** and 0.5 equiv. of $(PhS)_2$. (**a**) Initial ^{1}H NMR spectrum. (**b**) ^{1}H NMR spectrum after 1 day. (**c**) ^{1}H NMR spectrum after 2 days. (**d**) ^{1}H NMR spectrum after 3 days. (**e**) ^{1}H NMR spectrum after 7 days showing consumption of complex **1-Pu** and formation of **2-Pu** and $(Cp^{Me4})_2$.[1] A slight excess of $(PhS)_2$ was found in the reaction mixture most likely due to a weighing error in the small quantities used for **1-Pu** and $(PhS)_2$.

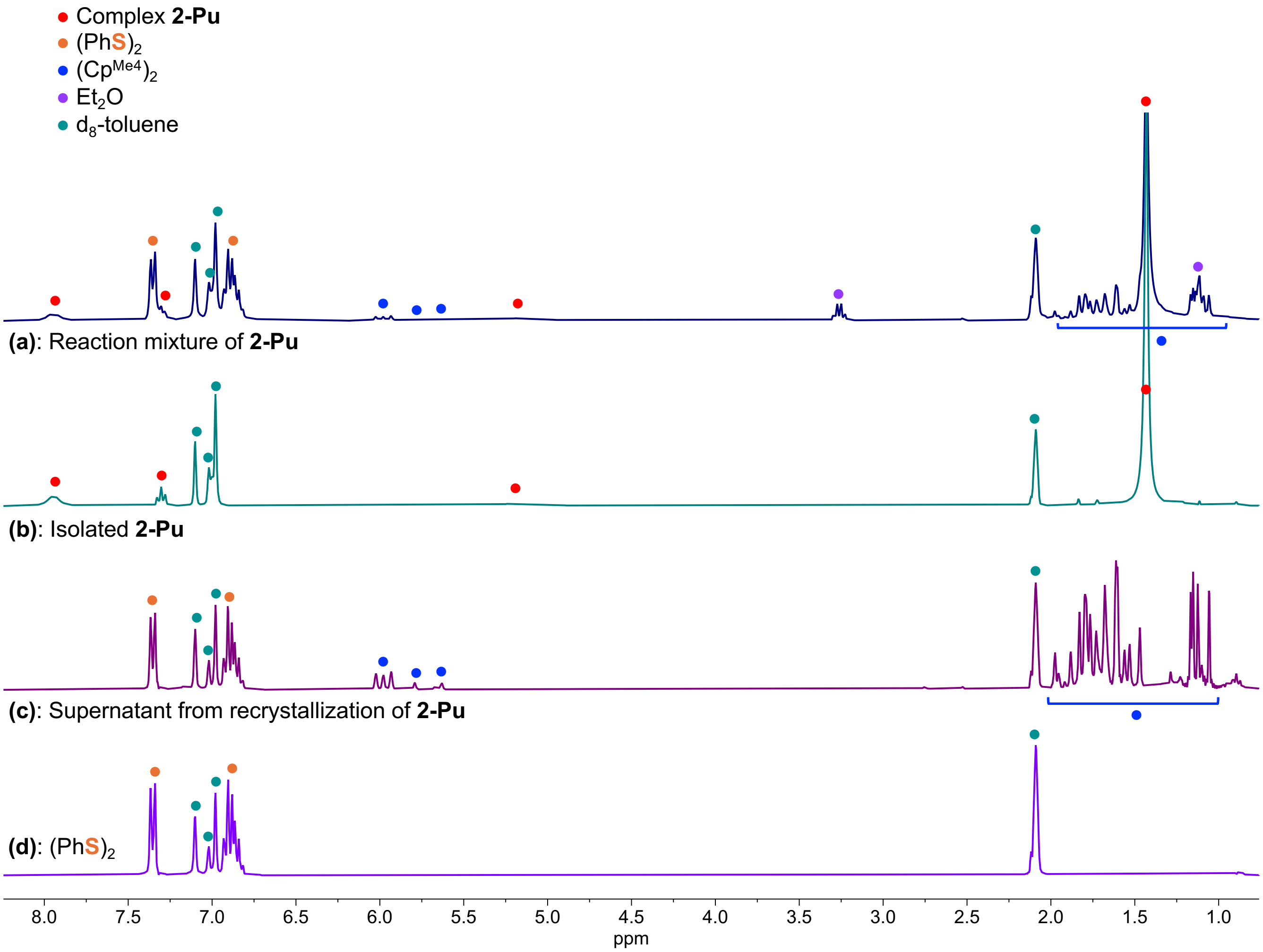

Supplementary Figure 12. ^{1}H NMR spectrum comparisons for the reaction of **1-Pu** and $(PhS)_2$. (**a**) ^{1}H NMR (400 MHz, d_8-toluene, 298K) spectra from reaction monitoring of complex **1-Pu** and 0.5 equiv. of $(PhS)_2$ after7 days. (**b**) ^{1}H NMR spectrum of isolated complex **2-Pu**. (**c**) ^{1}H NMR spectrum of the supernatant obtained from the isolation of **2-Pu**, showing $(Cp^{Me4})_2$[1] and residual $(PhS)_2$. (**d**) ^{1}H NMR spectrum of $(PhS)_2$.

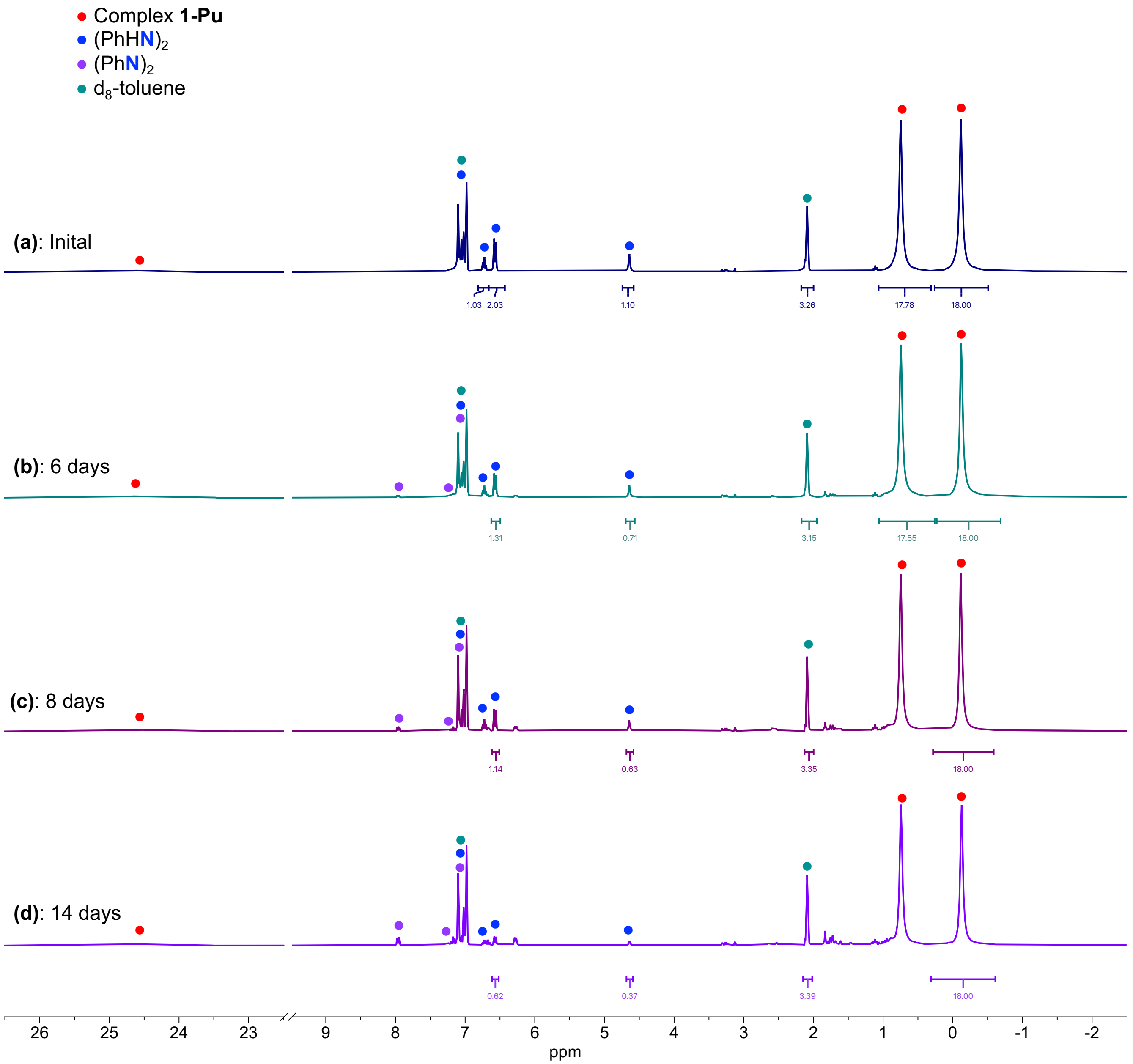

Supplementary Figure 13. ^{1}H NMR (400 MHz, d_8-toluene, 298K) spectra from reaction monitoring of complex **1-Pu** and 0.5 equiv. of $(PhHN)_2$. (**a**) Initial ^{1}H NMR spectrum. (**b**) ^{1}H NMR spectrum after 6 days. (**c**) ^{1}H NMR spectrum after 8 days. (**d**) ^{1}H NMR spectrum after 14 days showing no consumption of complex **1-Pu** integrated relative to the d_8-toluene peak. The only reactivity observed is the decomposition of the $(PhHN)_2$ starting material.

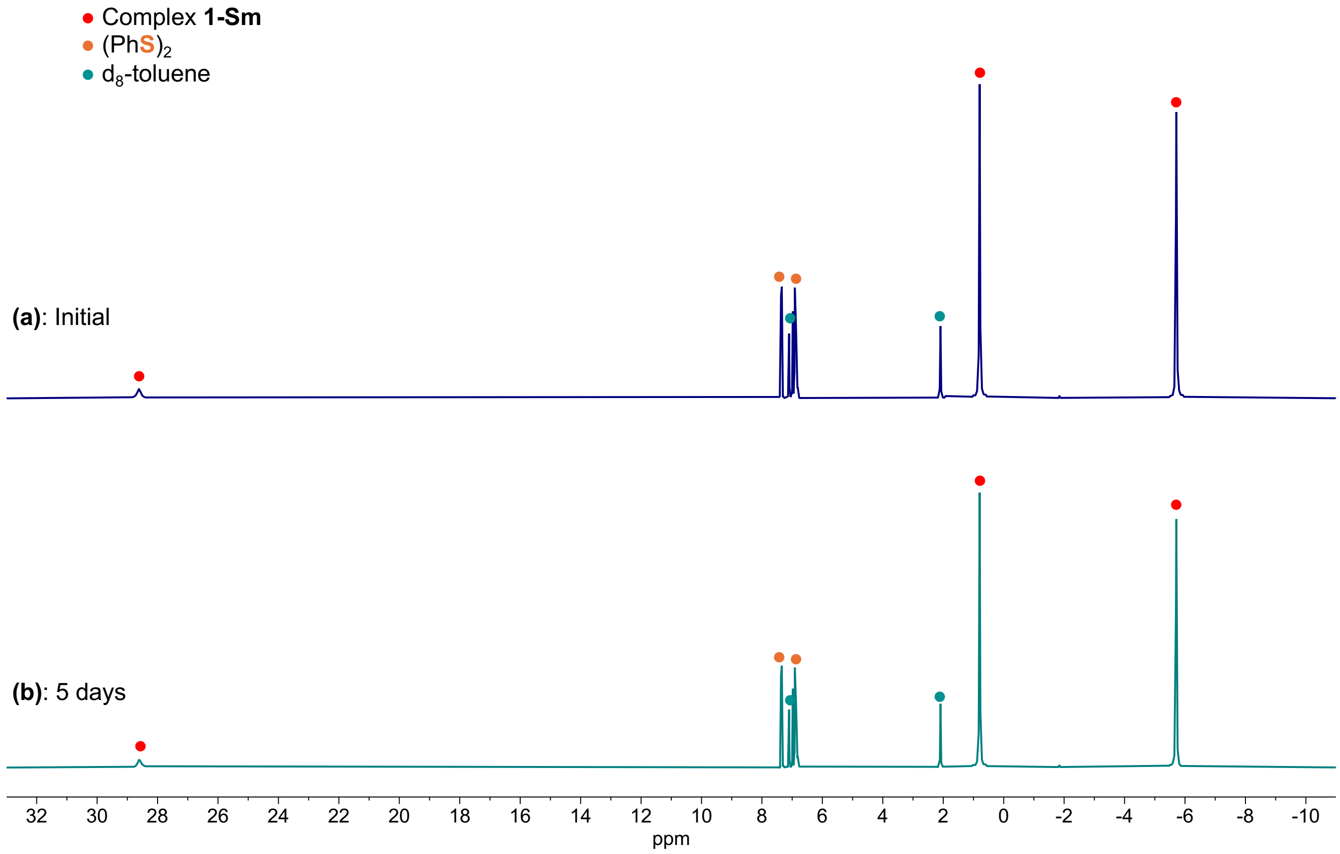

Supplementary Figure 14. ^{1}H NMR (400 MHz, d_8-toluene, 298K) spectra from reaction monitoring of complex **1-Sm** and 0.5 equiv. of $(PhHN)_2$. (**a**) Initial ^{1}H NMR spectrum. (**b**) ^{1}H NMR spectrum after 5 days showing no consumption of complex **1-Sm**.

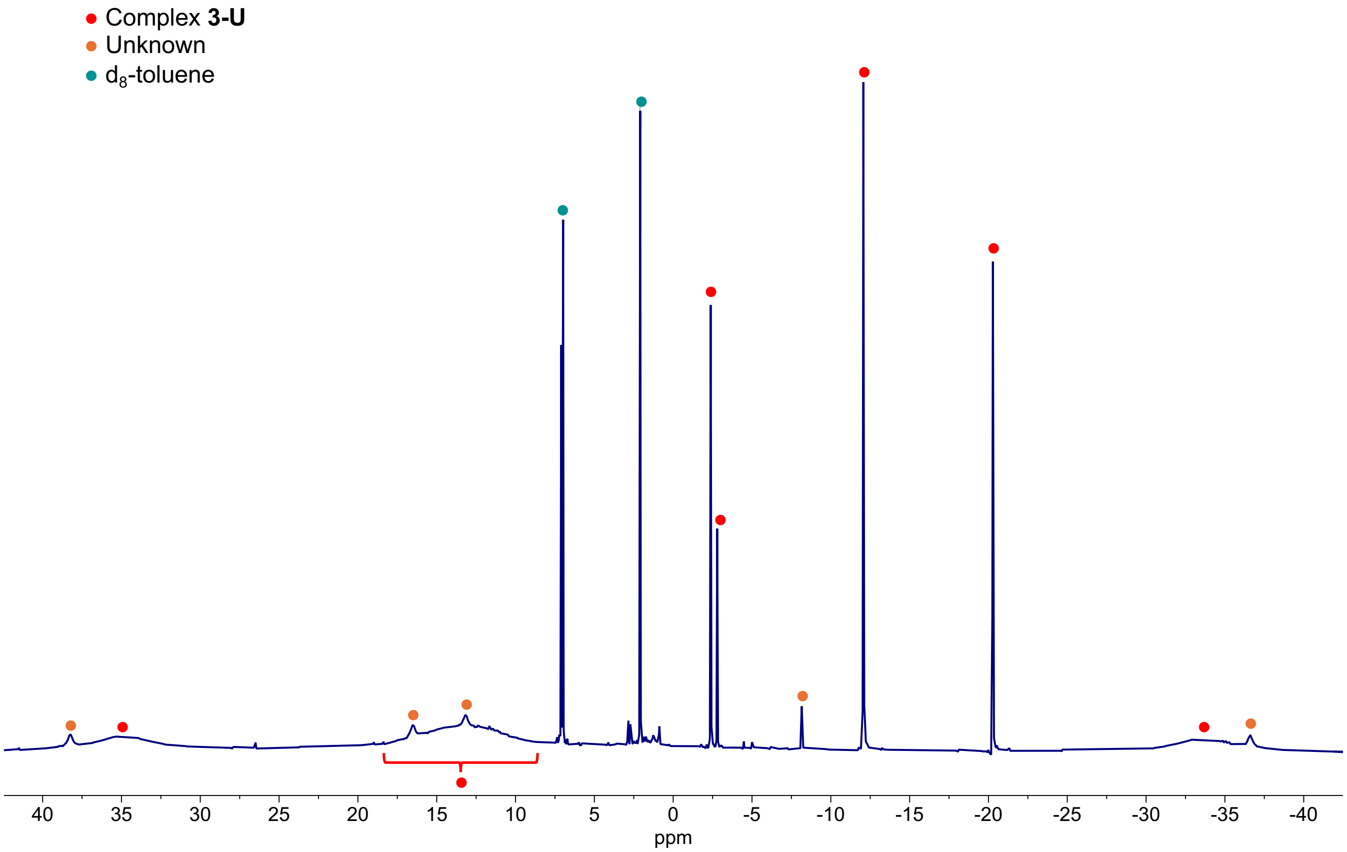

Supplementary Figure 15. ^{1}H NMR (400 MHz, d_8-toluene, 298K) spectra from reaction monitoring of complex **1-U** and 0.5 equiv. of $(PhS)_2$, resulting in immediate consumption of complex **1-U** and the formation of complex **3-U**. The reaction mixture was monitored over the course of 5 days, in which the spectrum was unchanged, and the unknown signals most likely correspond to an undetermined impurity.

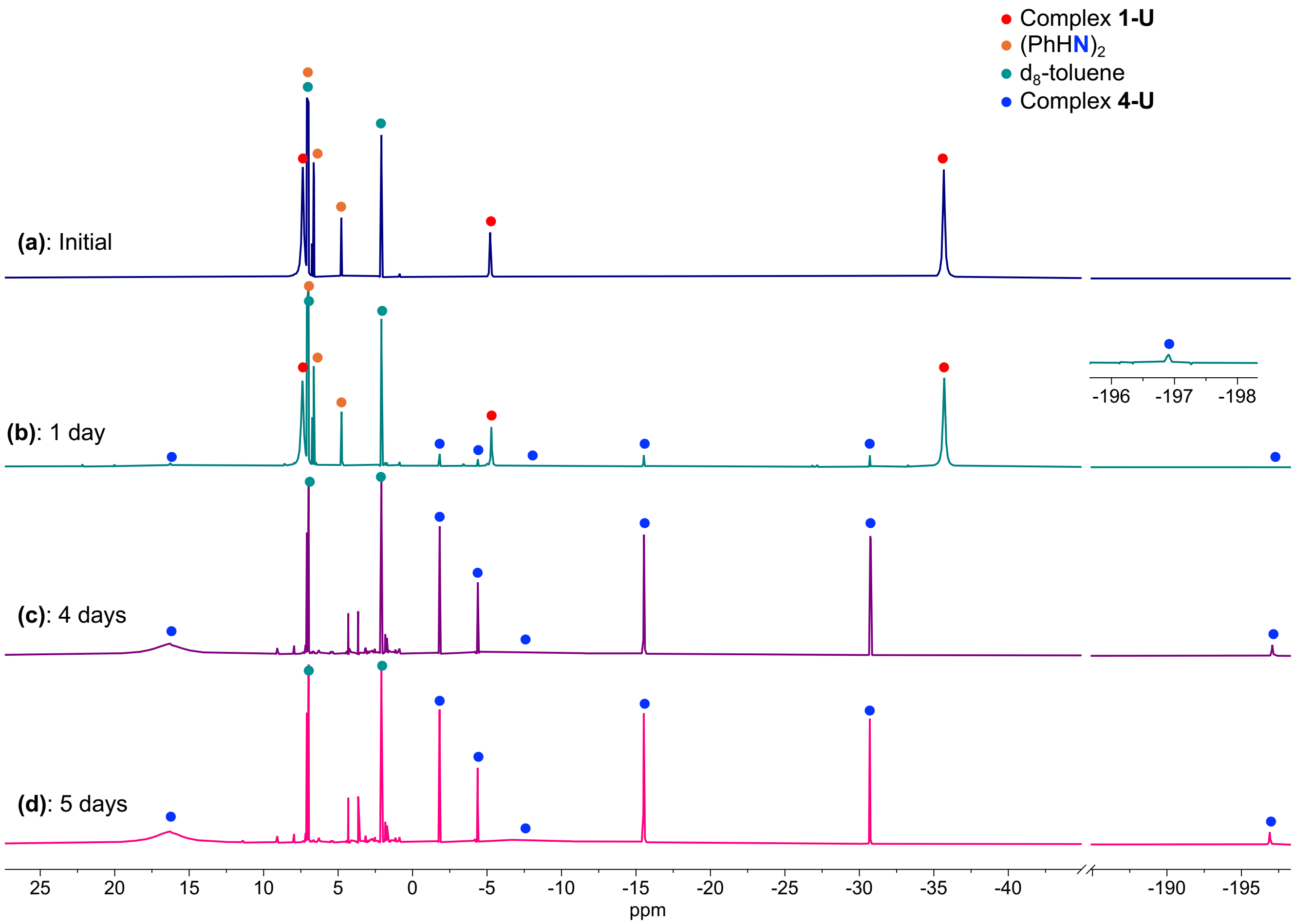

Supplementary Figure 16. ^{1}H NMR (400 MHz, *d8*-toluene, 298K) spectra from reaction monitoring of complex **1-U** and 0.5 equiv. of $(PhHN)_2$. (**a**) Initial ^{1}H NMR spectrum. (**b**) ^{1}H NMR spectrum after 1 day. (**c**) ^{1}H NMR spectrum after 4 days. (**d**) ^{1}H NMR spectrum after 5 days showing consumption of complex **1-U** and formation of **4-U**.

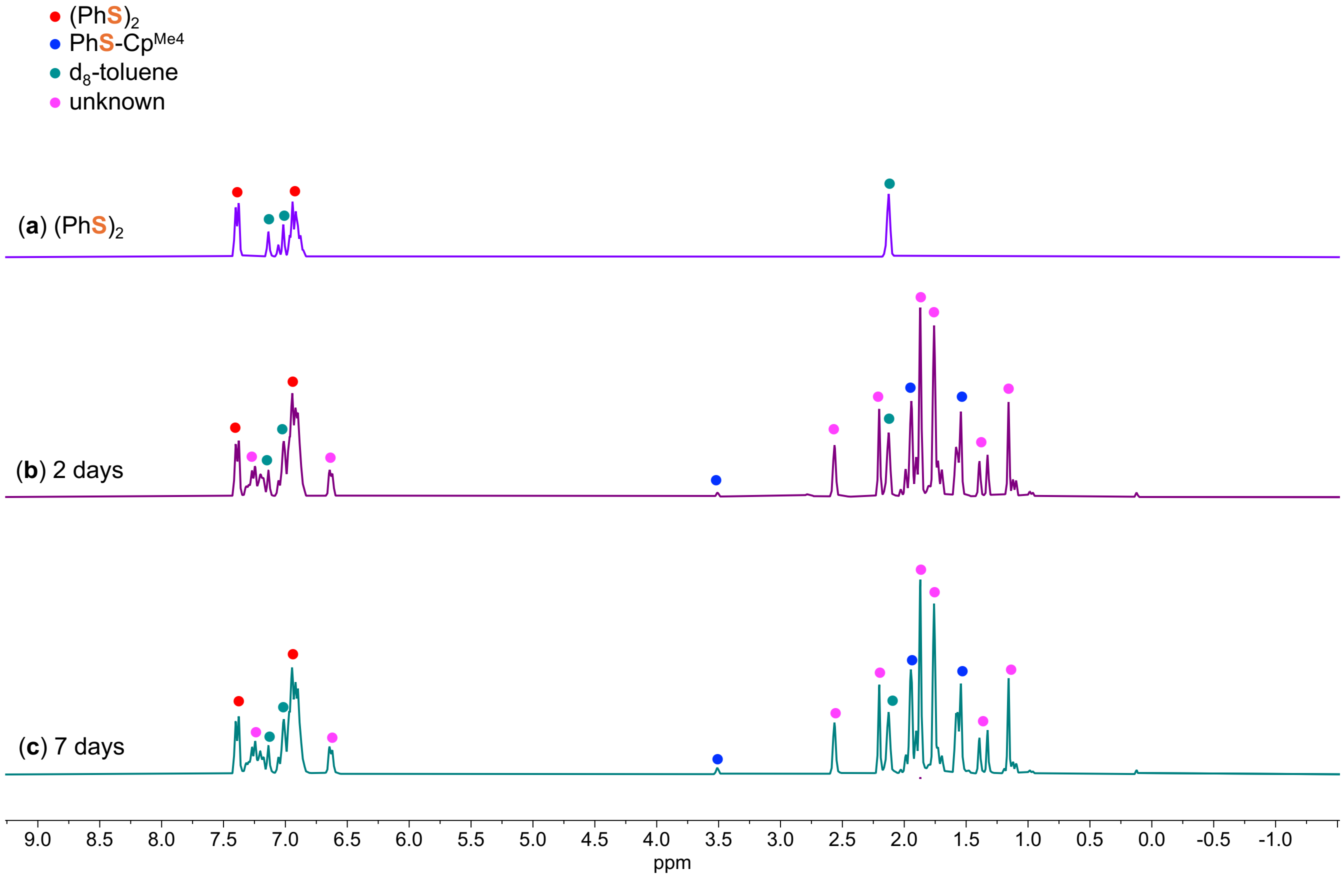

Supplementary Figure 17. ^{1}H NMR (400 MHz, d_8-toluene, 298K) spectra from reaction monitoring of $(PhS)_2$ and KCp^{Me4}. (**a**) ^{1}H NMR spectrum of $(PhS)_2$. (**b**, **c**) ^{1}H NMR spectrum after 2 and 7 days, respectively, showing PhS-Cp^{Me4}, unidentifiable material, and no $(Cp^{Me4})_2$, indicating that complex **1-Pu** is required for reductive cleavage reactivity.

S3. Supplementary X-Ray Crystallography Data

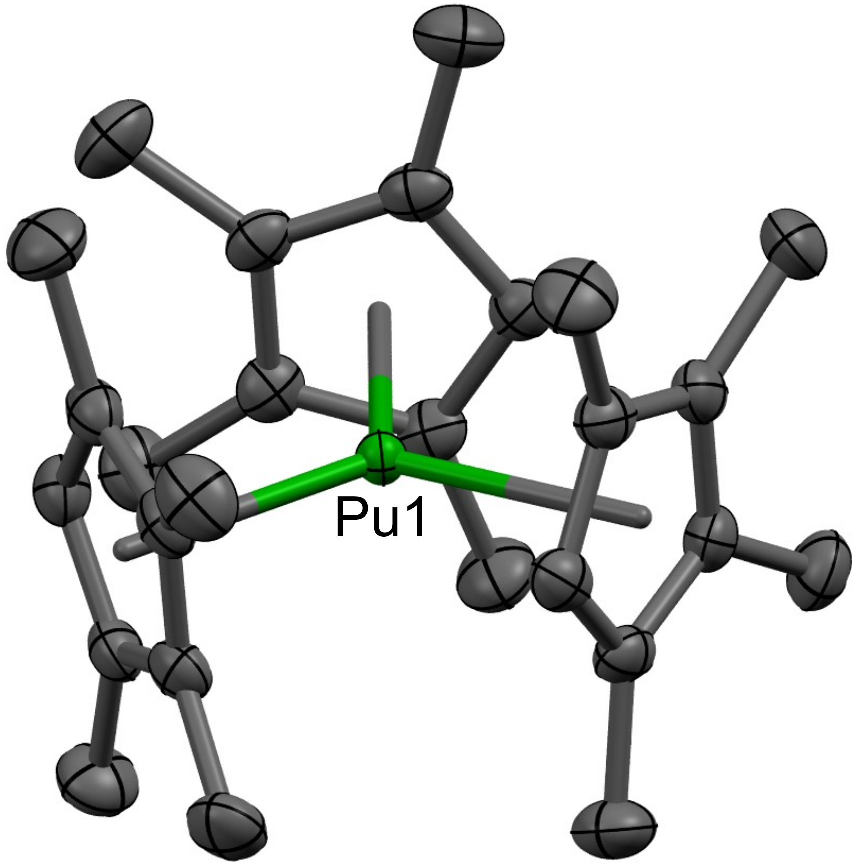

Supplementary Figure 18. Molecular structure of [$Pu^{III}(Cp^{Me4})_3$] **1-Pu**. Thermal ellipsoids are drawn at the 50% probability level. Hydrogen atoms have been omitted for clarity. Pu (green) and C (dark grey). CCDC number 2446212.

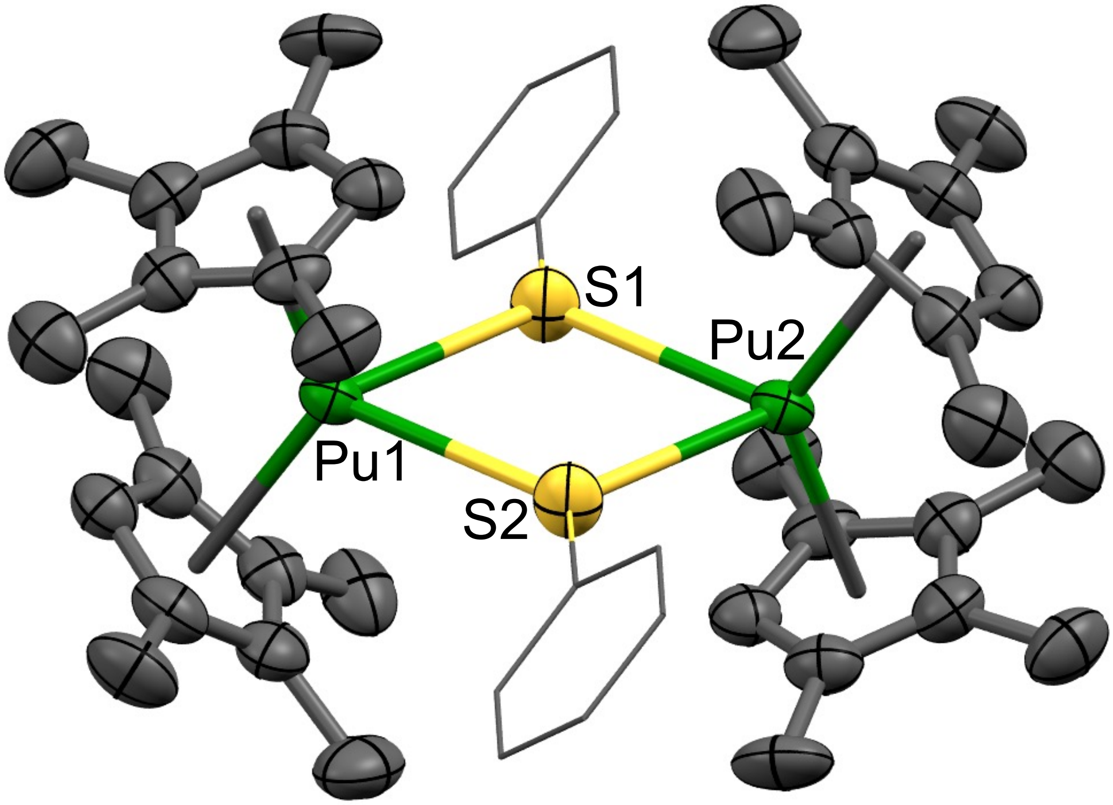

Supplementary Figure 19. Molecular structure of $[\{Pu^{III}(Cp^{Me4})_2\}_2(\mu\text{-}SPh)_2]$, **2-Pu**. Thermal ellipsoids are drawn at the 50% probability level. Hydrogen atoms have been omitted for clarity. Pu (green), S (yellow), and C (dark grey). CCDC number 2446213.

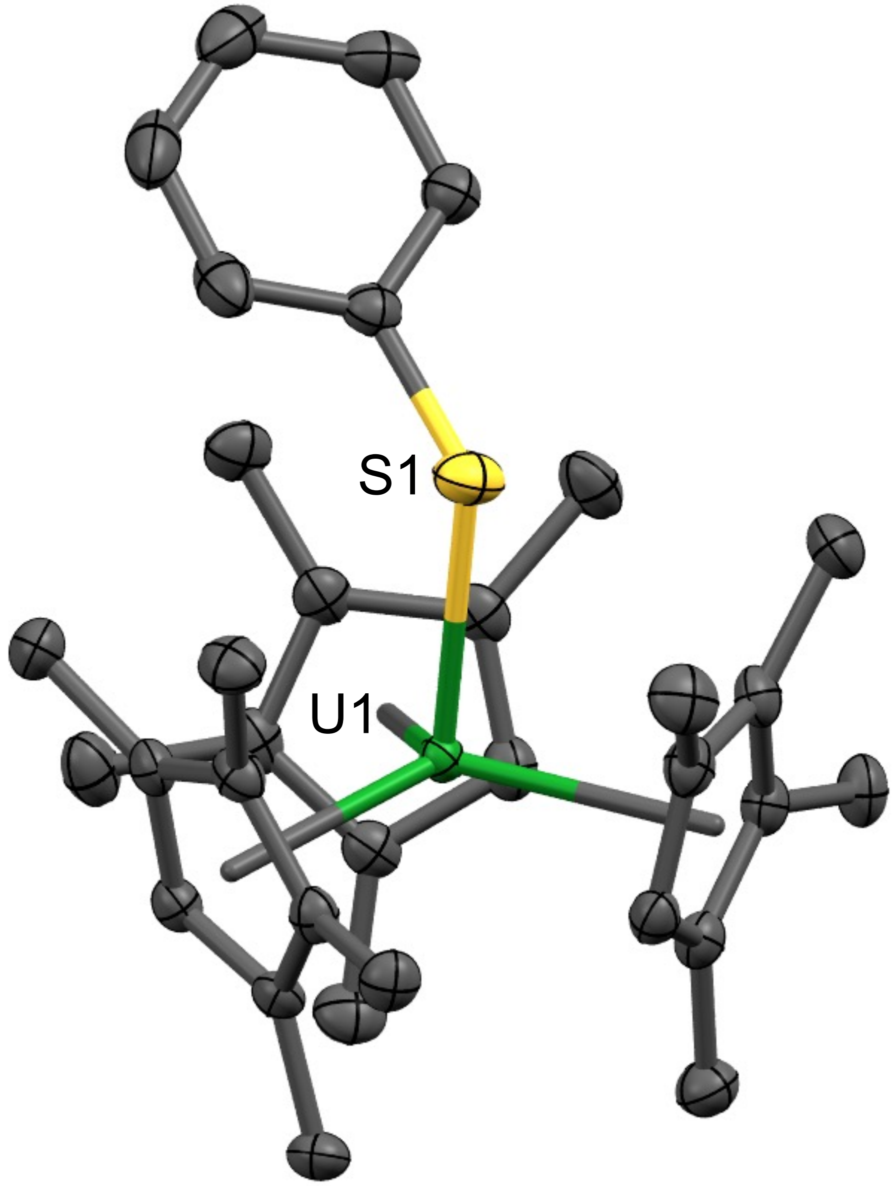

Supplementary Figure 20. Molecular structure of [$U^{IV}(Cp^{Me4})_3(SPh)$], **3-U**. Thermal ellipsoids are drawn at the 50% probability level. Hydrogen atoms have been omitted for clarity. Pu (green), S (yellow), and C (dark grey). CCDC number 2446214.

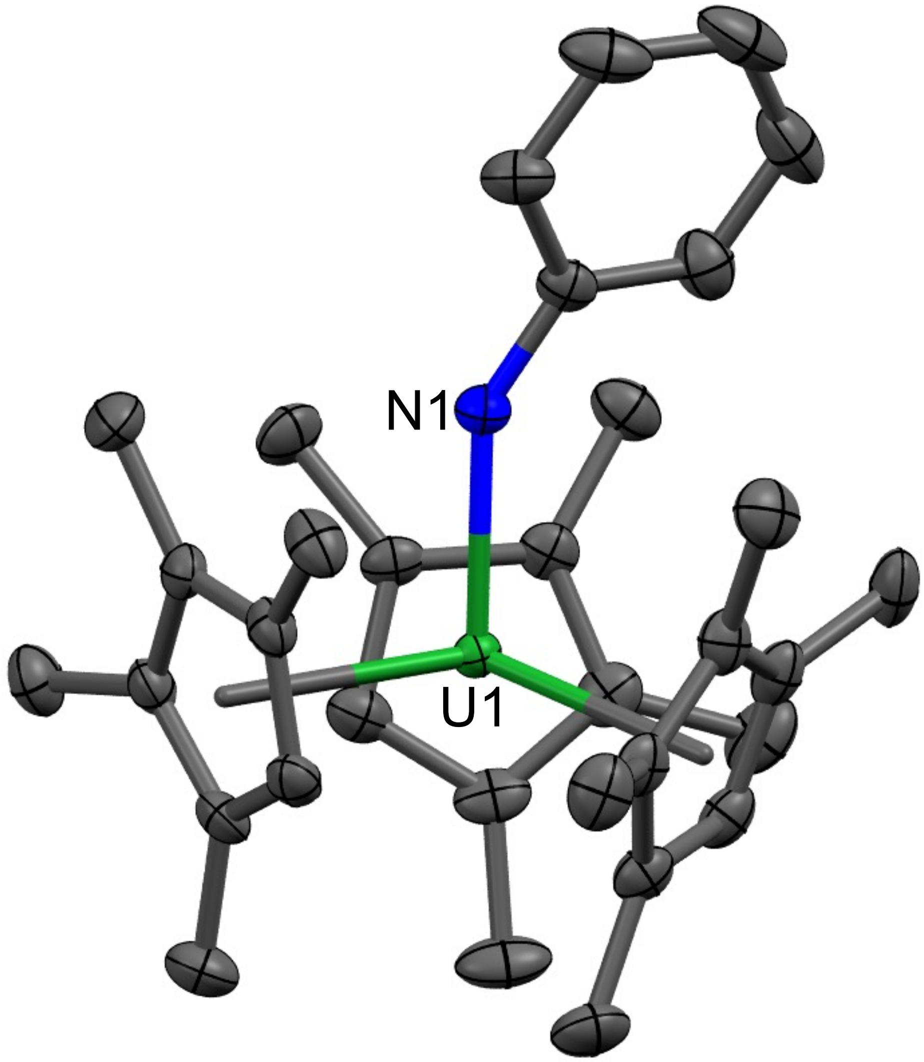

Supplementary Figure 21. Molecular structure of [$U^{IV}(Cp^{Me4})_3$(NHPh)], **4-U**. Thermal ellipsoids are drawn at the 50% probability level. Hydrogen atoms have been omitted for clarity. U (green), N (blue), and C (dark grey). CCDC number 2446215.

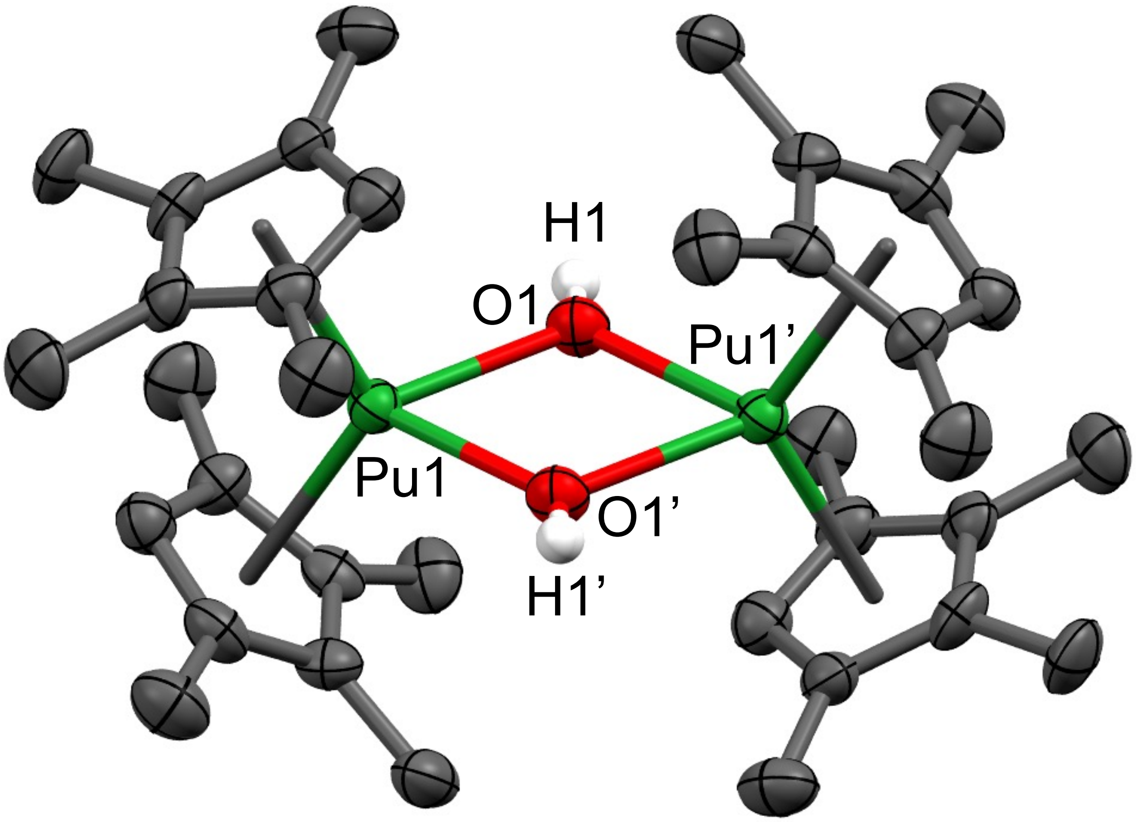

Supplementary Figure 22. Molecular structure of [{$Pu^{III}(Cp^{Me4})_2$}$_2$(μ-OH)$_2$], **5-Pu**. Thermal ellipsoids drawn at the 50% probability level. Hydrogen atoms have been omitted for clarity. Pu1–O1: 2.337(6) Å; U1–centroid: [2.473(4); 2.490 (4) Å]. The bond metrics in **2-Pu** are consistent with the previously reported, [{$U^{III}(Cp'')_2$}$_2$(μ-OH)$_2$] (Cp'' = {C_5H_3(1,3-$SiMe_3$)$_2$}$^{-1}$).[2] Pu (green), O (red), H (white), and C (dark grey). CCDC number 2446216.

Supplementary Table 1. Displacements (Å) for the four unique methyl substituents from the average ring carbon plane of the $(Cp^{Me4})^{1-}$ ligand and the C(Me)–C(ring)–(average ring carbon plane) angles (θ, °).

Complex	C(9)	C(8)	C(7)	C(6)	θ[C5-C9]	θ[C4-C8]	θ[C3-C7]	θ[C2-C6]
**1-Pu**	0.077	0.216	0.225	0.107	2.9	8.3	8.6	4.1

Supplementary Equation 1. Calculation of C(Me)–C(ring)–(average ring carbon plane) angles (θ, °)

$$\theta = \arcsin \frac{\text{C(Me) displacement from mean ring plane}}{\text{C(Me)-C(ring)bond distance}}$$

S4. Solution and solid-state absorption spectra

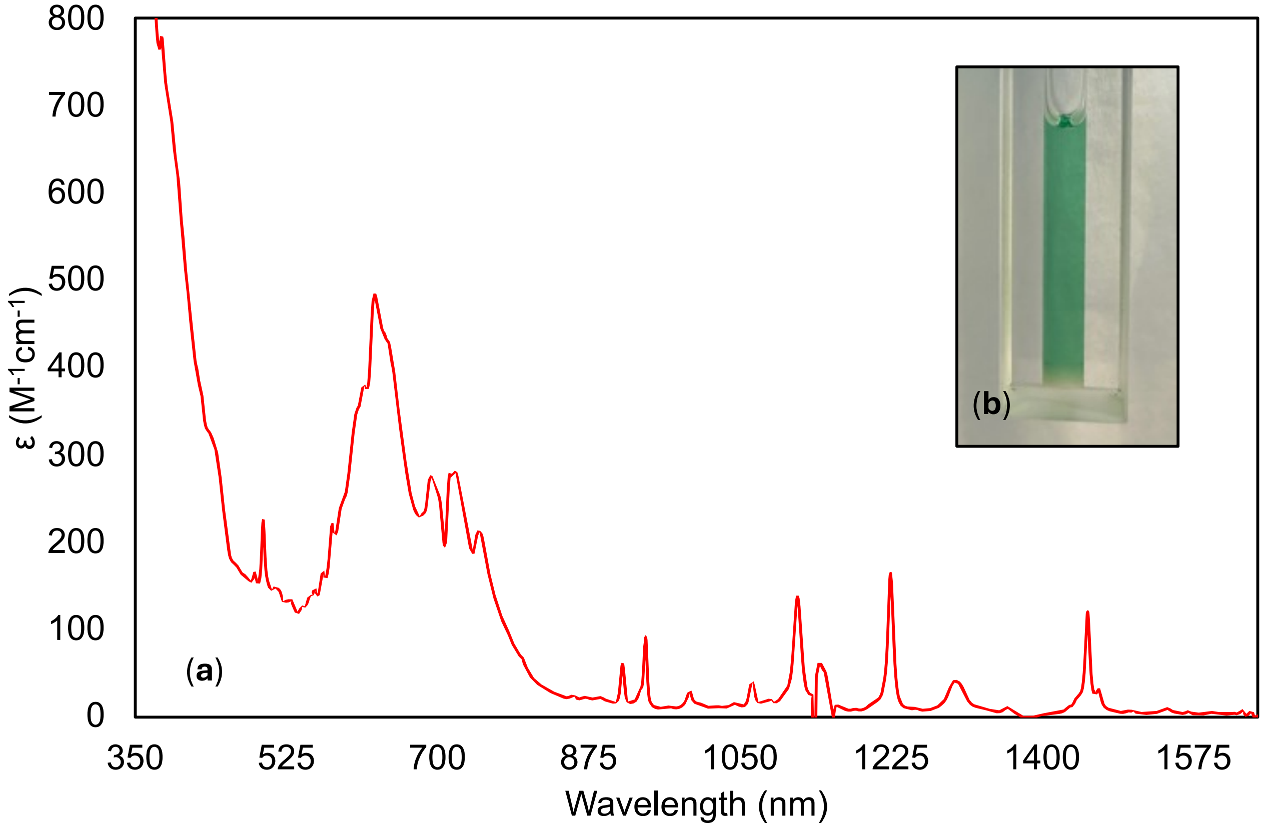

Supplementary Figure 23. Solution-state UV-Vis-NIR spectrum of complex **1-Pu**. (**a**) The spectrum was recorded between 350 to 1700 nm as a 1.3 mM solution in toluene at room temperature. (**b**) UV-Vis-NIR sample of complex **1-Pu**.

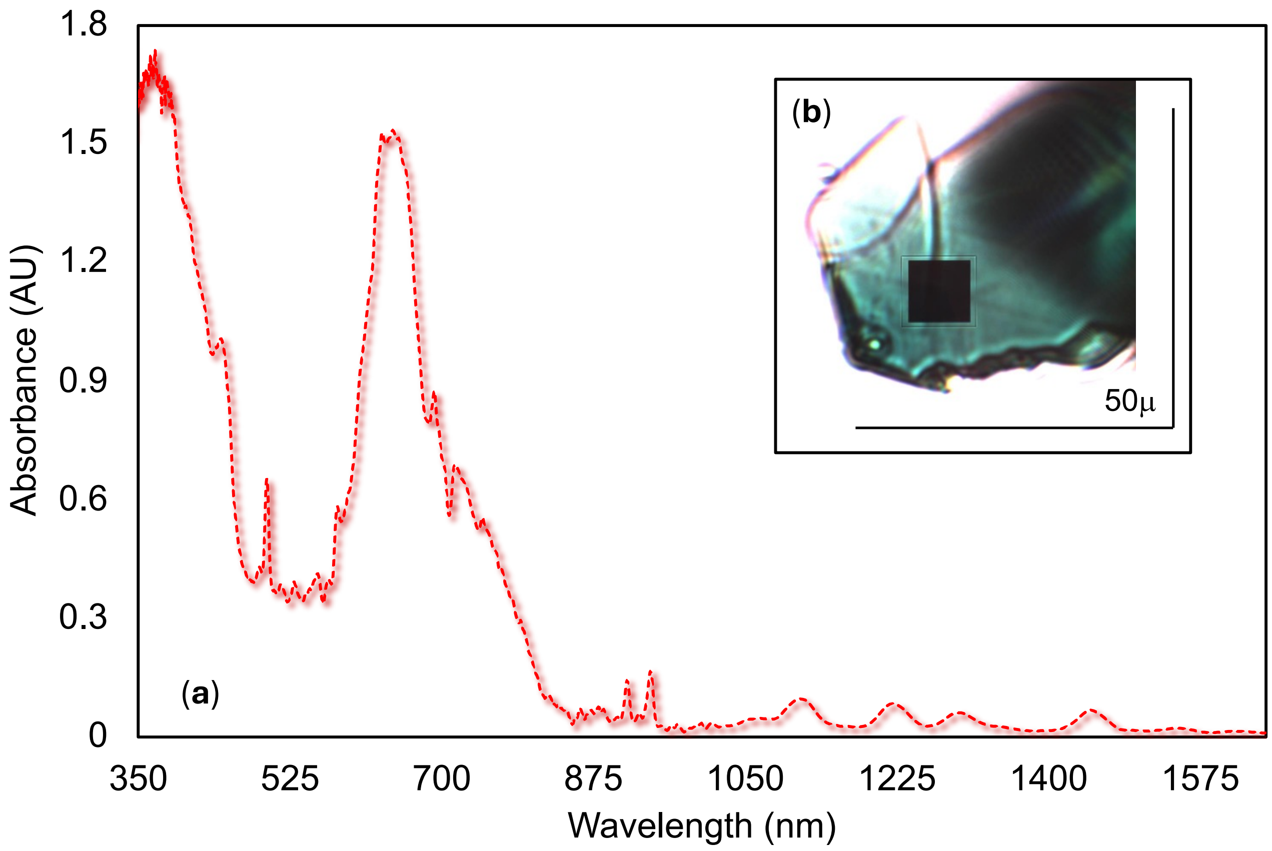

Supplementary Figure 24. Solid-state UV-Vis-NIR spectrum of complex **1-Pu**. (**a**) The spectrum was recorded between 350 to 1700 nm as (**b**) single crystals (Objective = 50X and Aperture = 3).

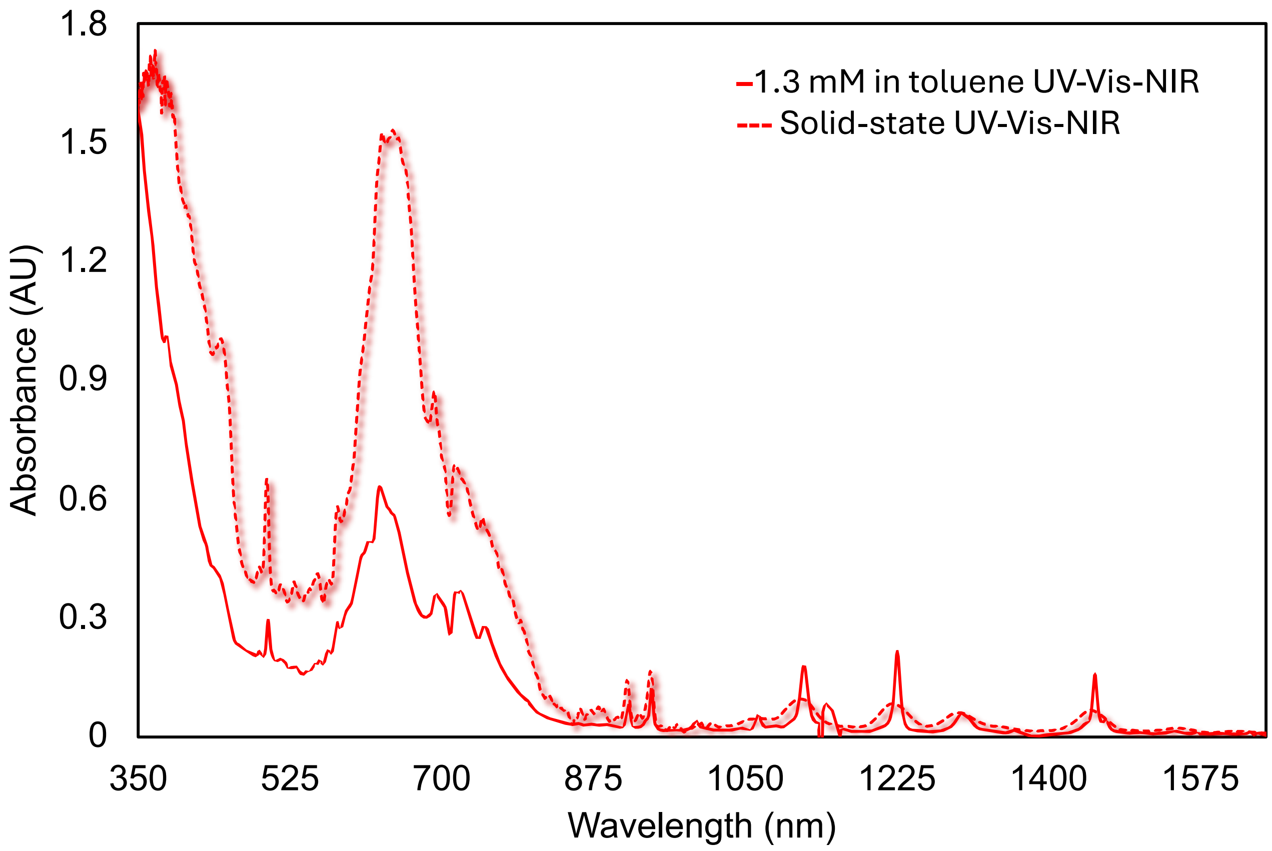

Supplementary Figure 25. Overlayed spectra for complex **1-Pu**. The (solid line) solution- and (dashed line) solid-state UV-Vis-NIR spectra for complex **1-Pu** between 350 to 1700 nm recorded as a 1.3 mM solution in toluene and single crystals, respectively, at room temperature.

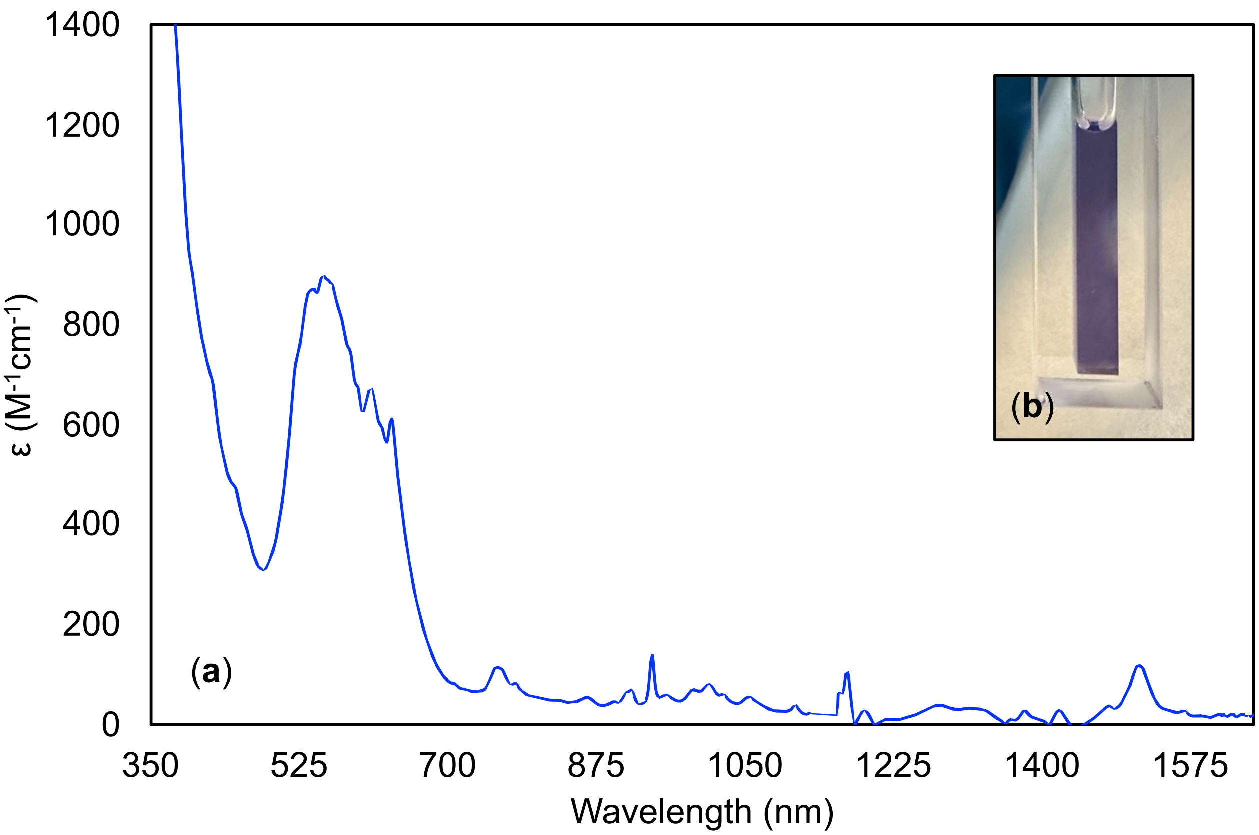

Supplementary Figure 26. Solution-state UV-Vis-NIR spectrum of complex **2-Pu**. (**a**) The spectrum was recorded between 350 to 1700 nm as a 1.0 mM solution in toluene at room temperature. (**b**) UV-Vis-NIR sample of complex **2-Pu**.

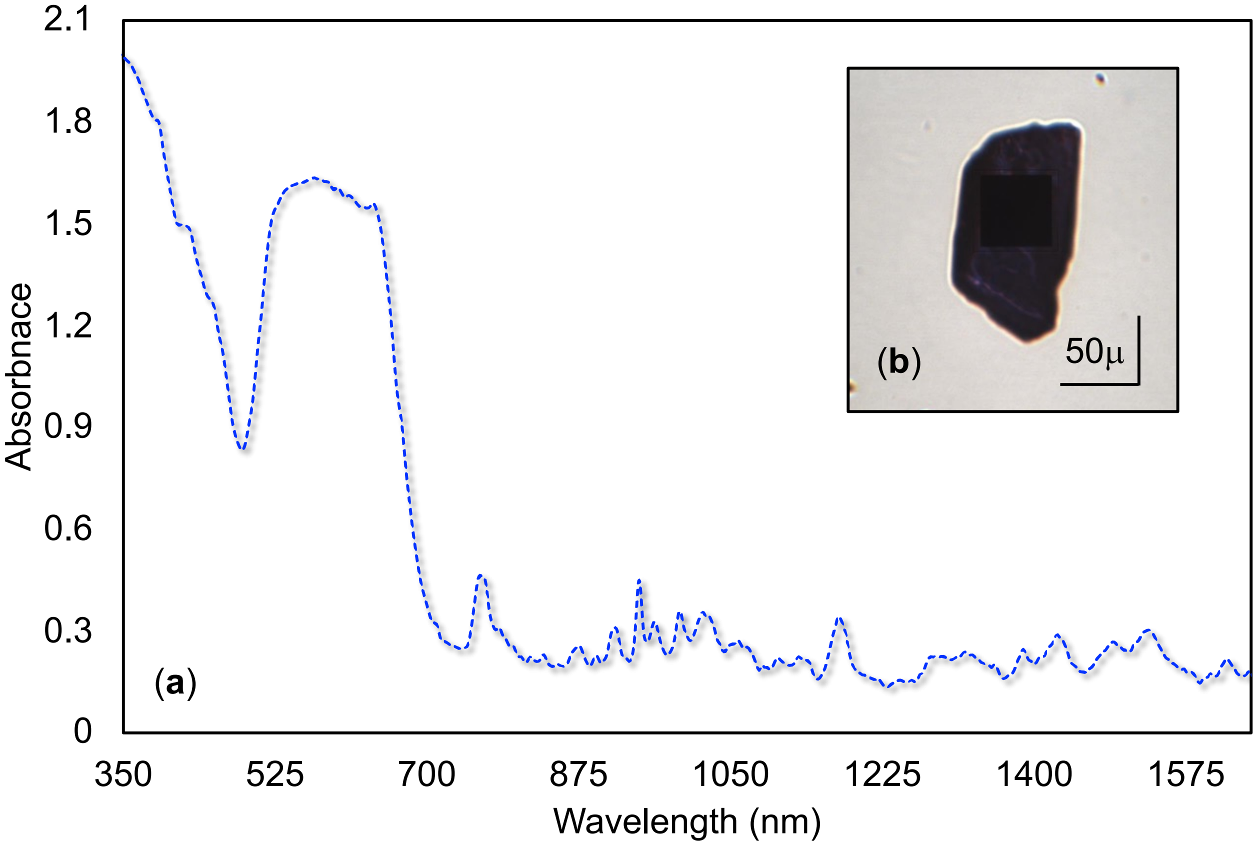

Supplementary Figure 27. Solid-state UV-Vis-NIR spectrum of complex **2-Pu**. The spectrum was recorded between 350 to 1700 nm as (**b**) single crystals (Objective = 10X and Aperture = 3).

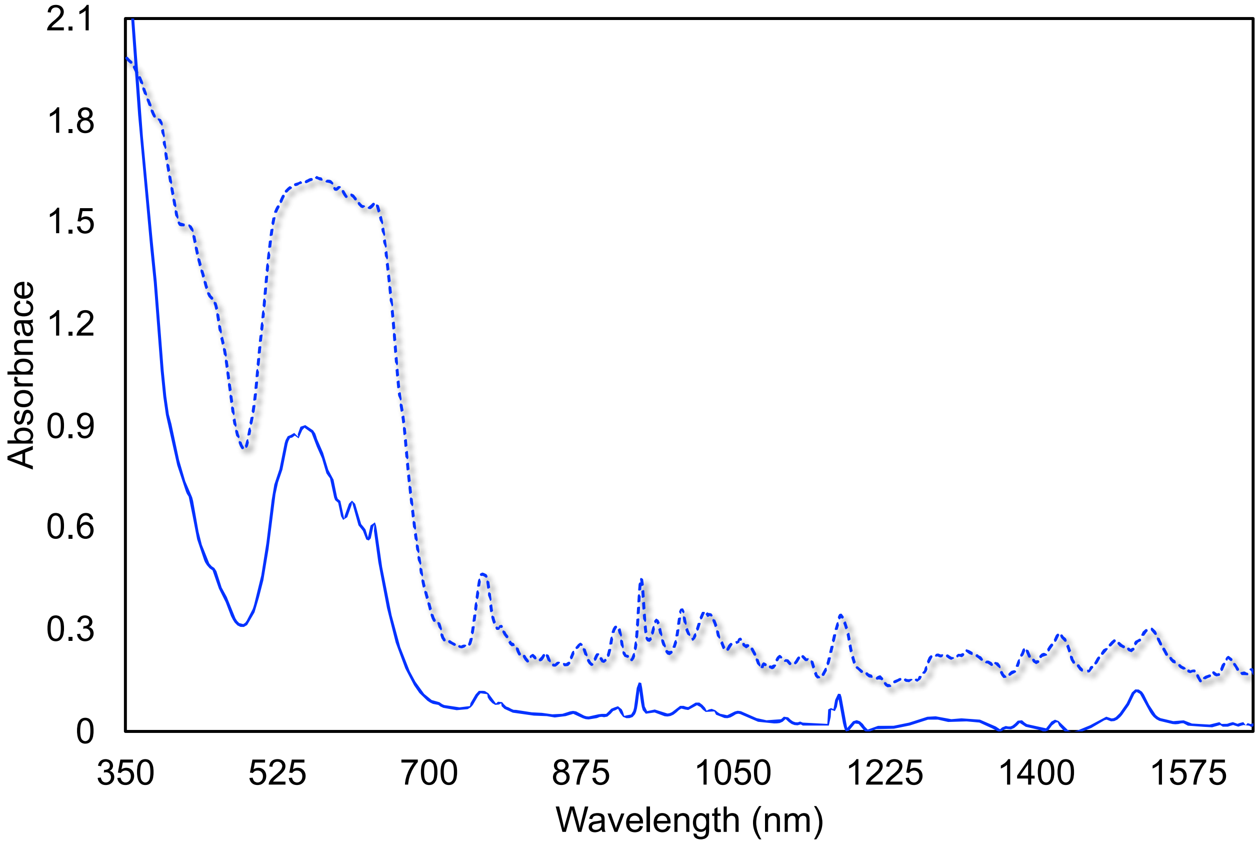

Supplementary Figure 28. Overlayed spectra for complex **2-Pu**. The (solid line) solution- and (dashed line) solid-state UV-Vis-NIR spectra for complex **2-Pu** between 350 to 1700 nm recorded as a 1.0 mM solution in toluene and single crystals, respectively, at room temperature.

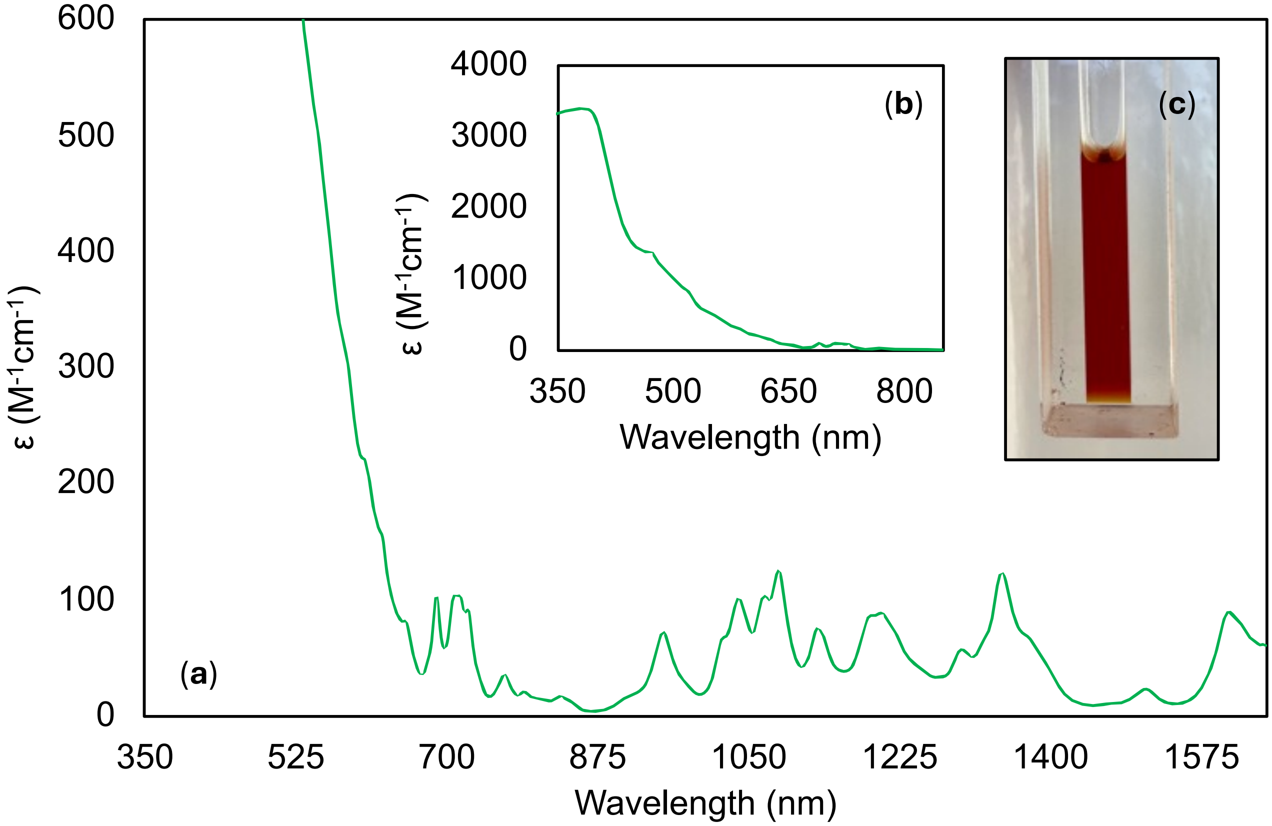

Supplementary Figure 29. Solution-state UV-Vis-NIR spectrum of complex **3-U.** (**a**) The spectrum was recorded between 350 to 1700 nm as a 1.0 mM solution in toluene at room temperature. (**b**) Zoomed-in inset between 350 to 850 nm. (**c**) UV-Vis-NIR sample of complex **3-U**.

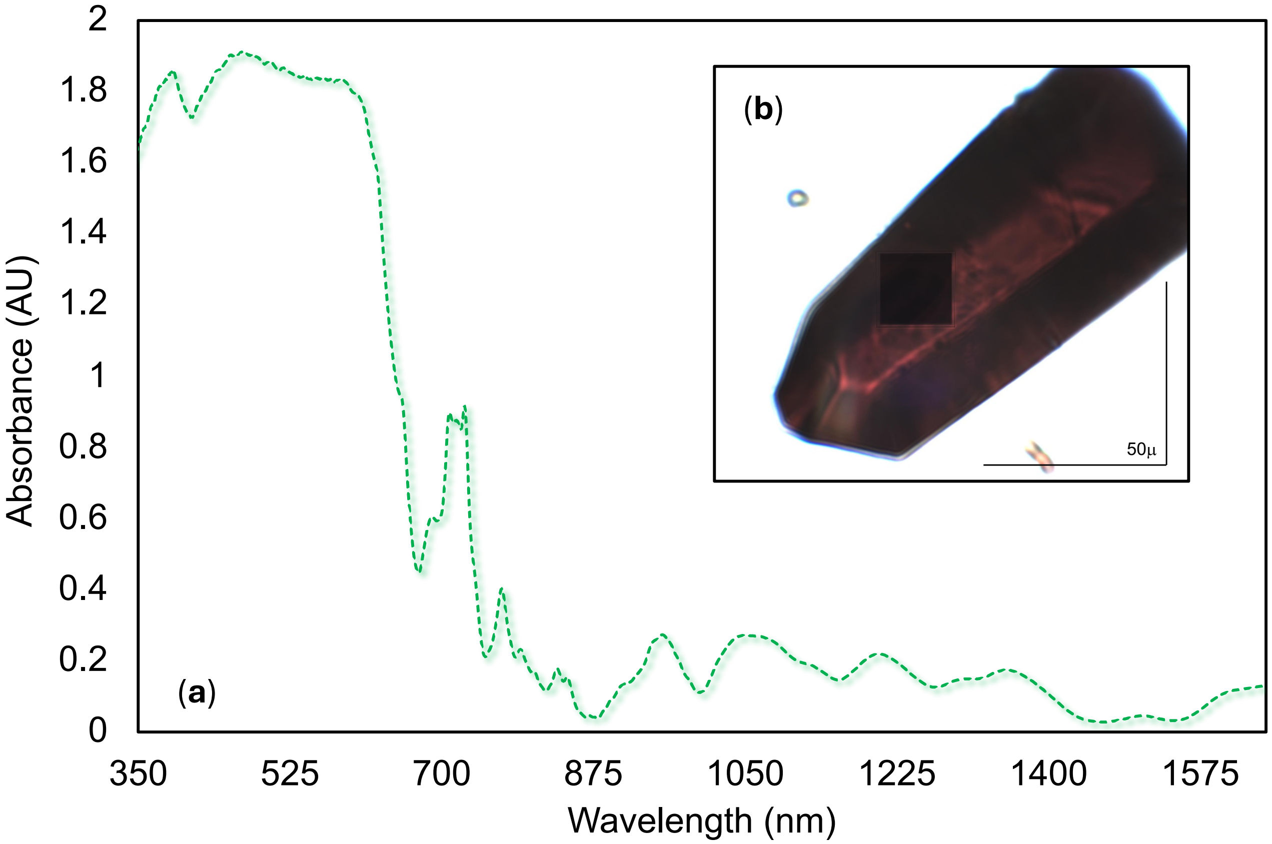

Supplementary Figure 30. Solid-state UV-Vis-NIR spectrum of complex **3-U.** (**a**) The spectrum was recorded between 350 to 1700 nm as (**b**) single crystals (Objective = 50X and Aperture = 1).

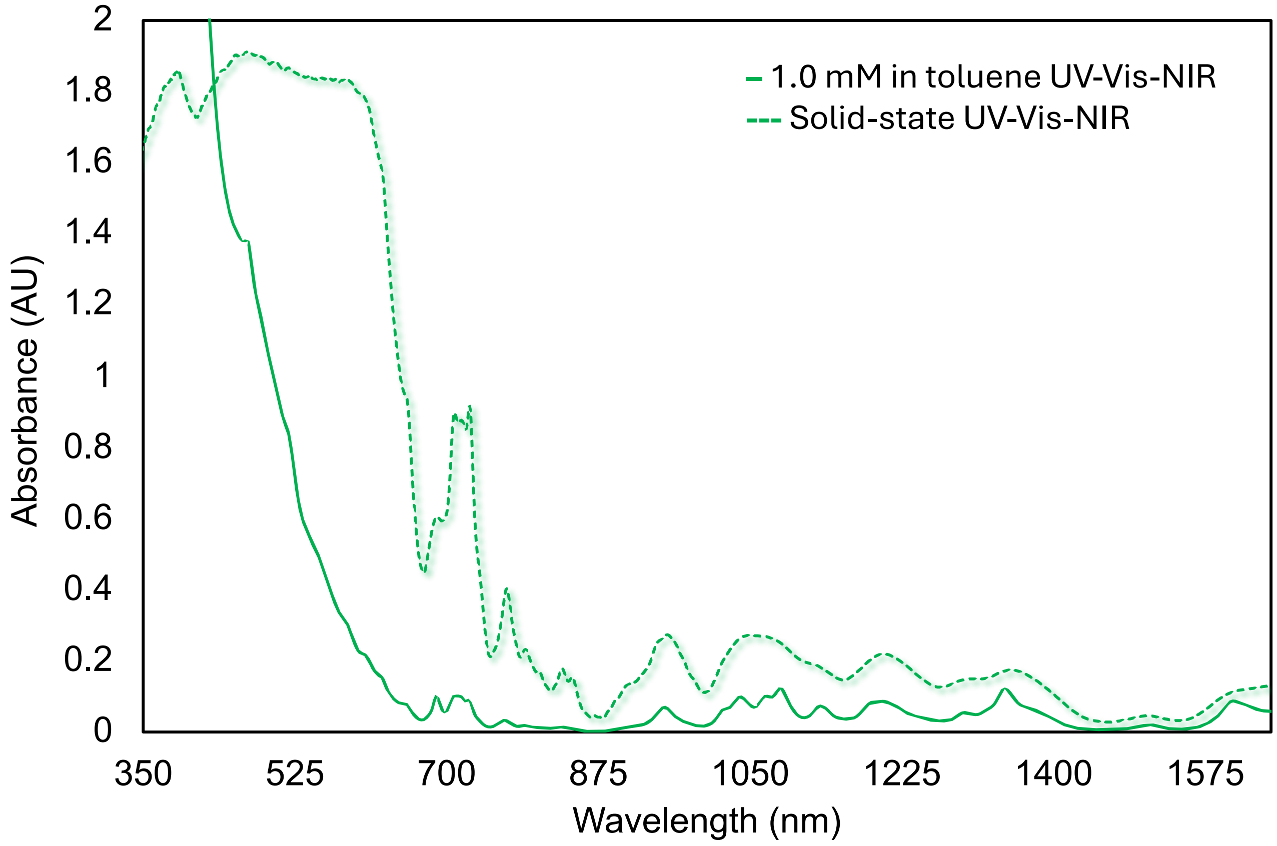

Supplementary Figure 31. Overlayed spectra for complex **3-U**. The (solid line) solution- and (dashed line) solid-state UV-Vis-NIR spectra for complex **3-U** between 350 to 1700 nm recorded as a 1.0 mM solution in toluene and single crystals, respectively, at room temperature.

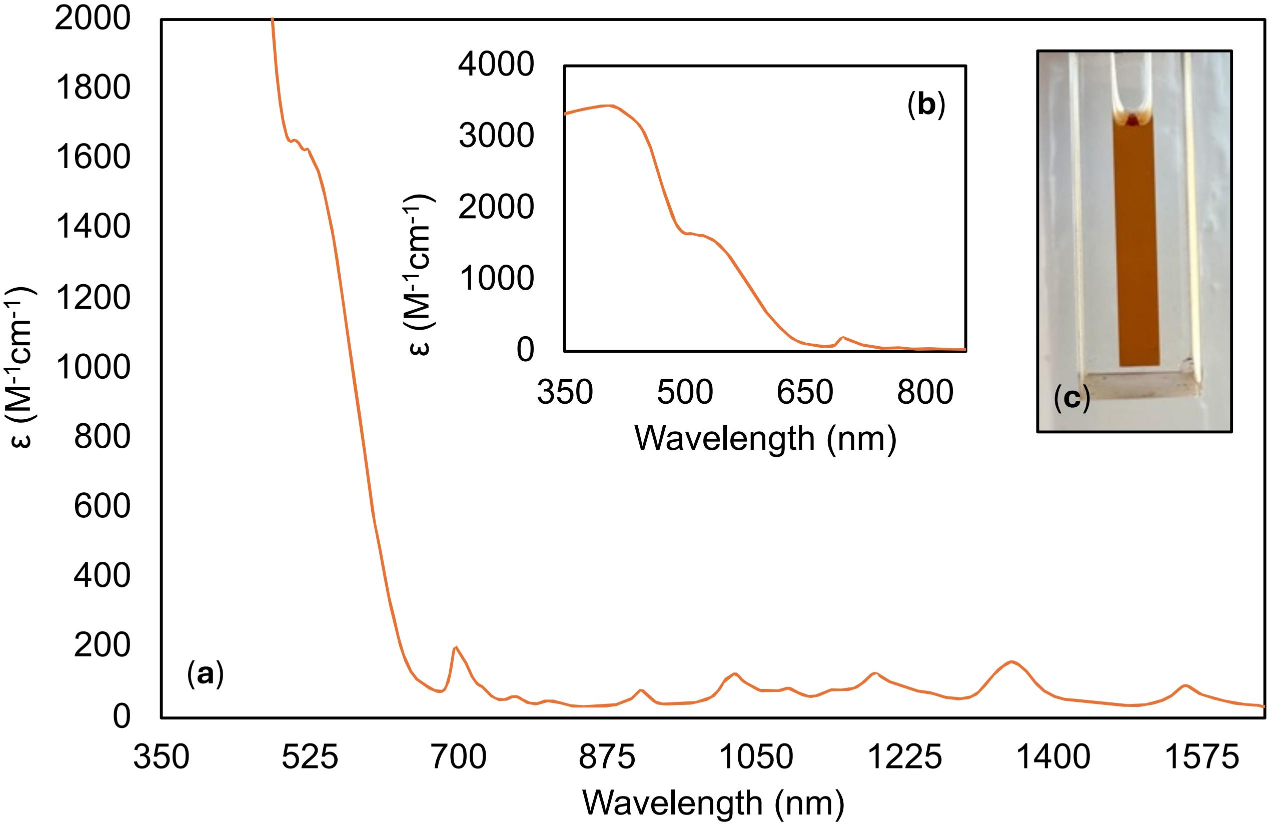

Supplementary Figure 32. Solution-state UV-Vis-NIR spectrum of complex **4-U**. (**a**) The spectrum was recorded between 350 to 1700 nm as a 1.0 mM solution in toluene at room temperature. (**b**) Zoomed-in inset between 350 to 850 nm. (**c**) UV-Vis-NIR sample of complex **4-U**.

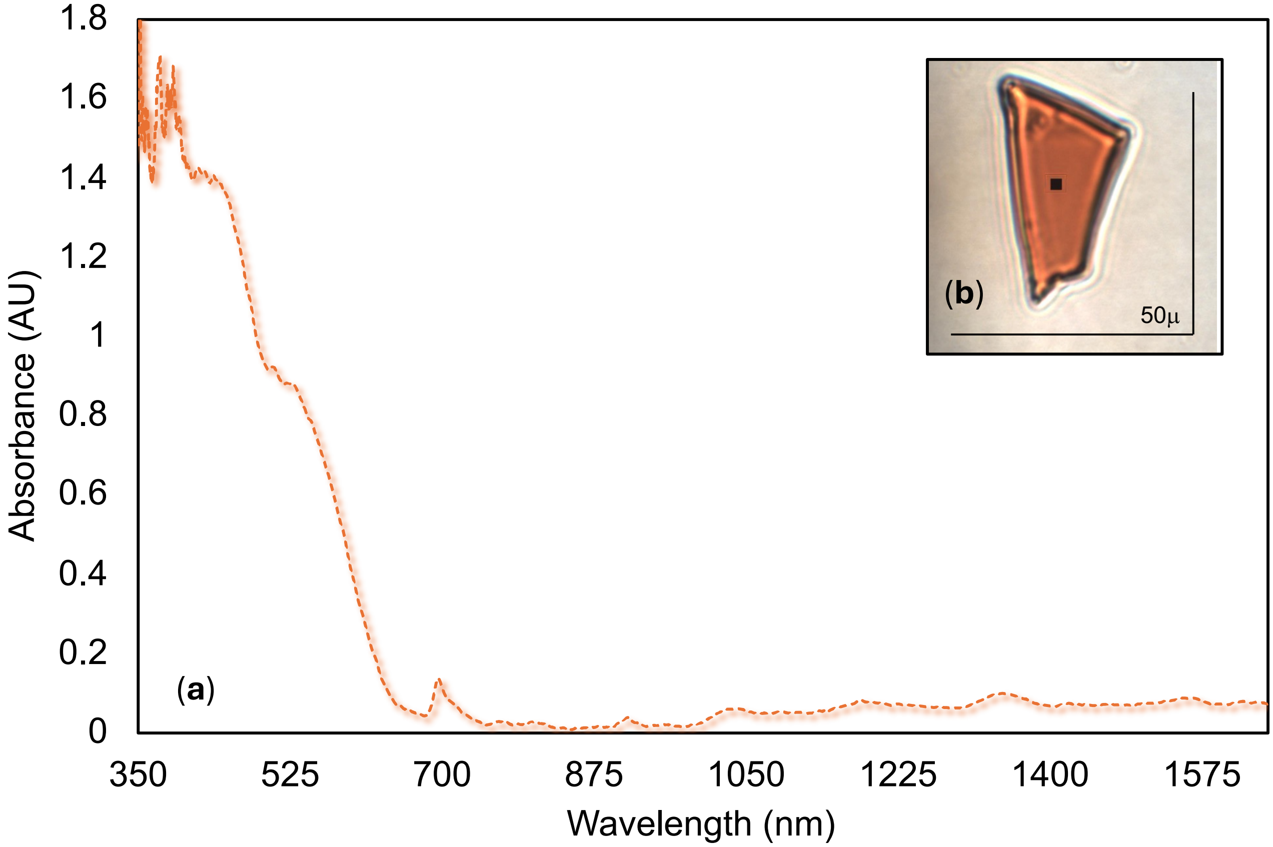

Supplementary Figure 33. Solid-state UV-Vis-NIR spectrum of complex **4-U**. (**a**) The spectrum was recorded between 350 to 1700 nm as (**b**) single crystals (Objective = 50X and Aperture = 5).

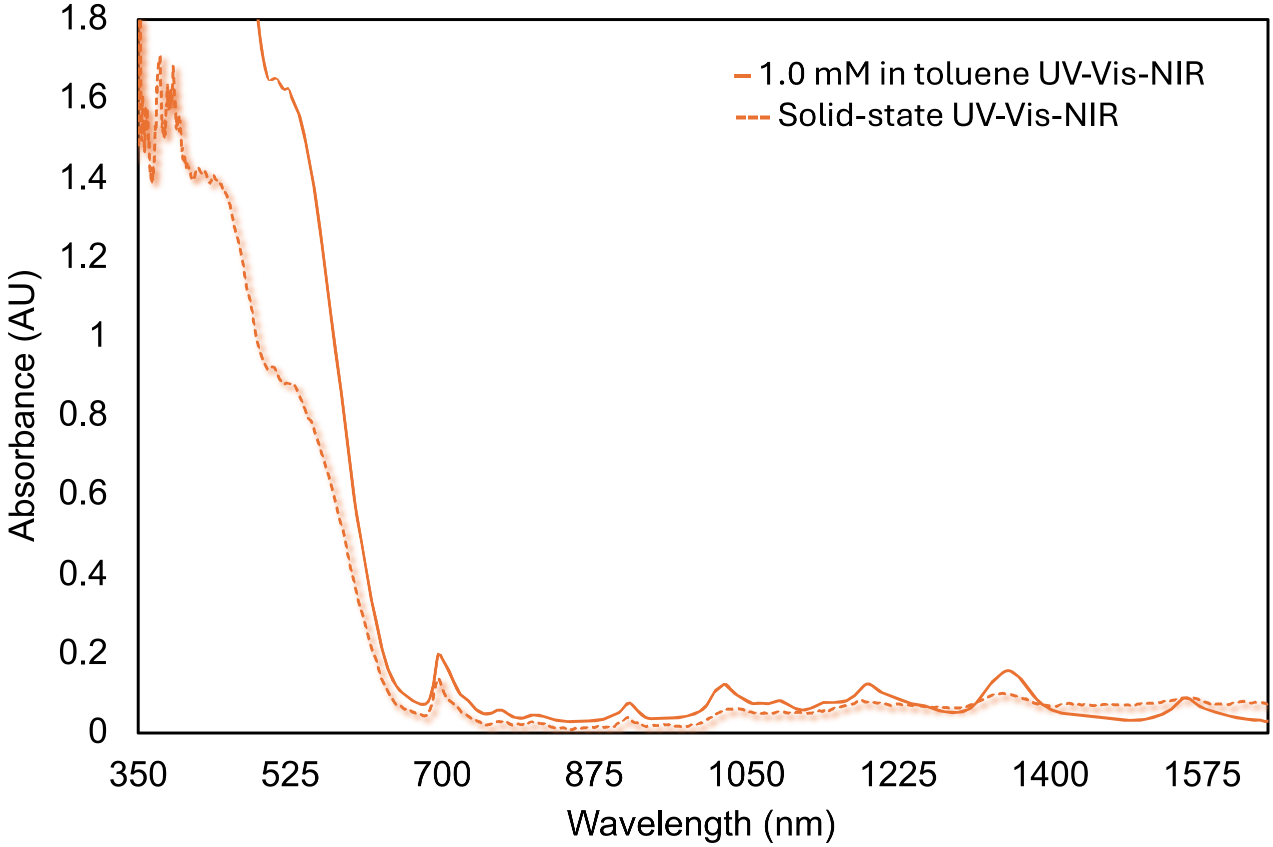

Supplementary Figure 34. Overlayed spectra for complex **4-U**. The (solid line) solution- and (dashed line) solid-state UV-Vis-NIR spectra for complex **4-U** between 350 to 1700 nm recorded as a 1.0 mM solution in toluene and single crystals, respectively, at room temperature.

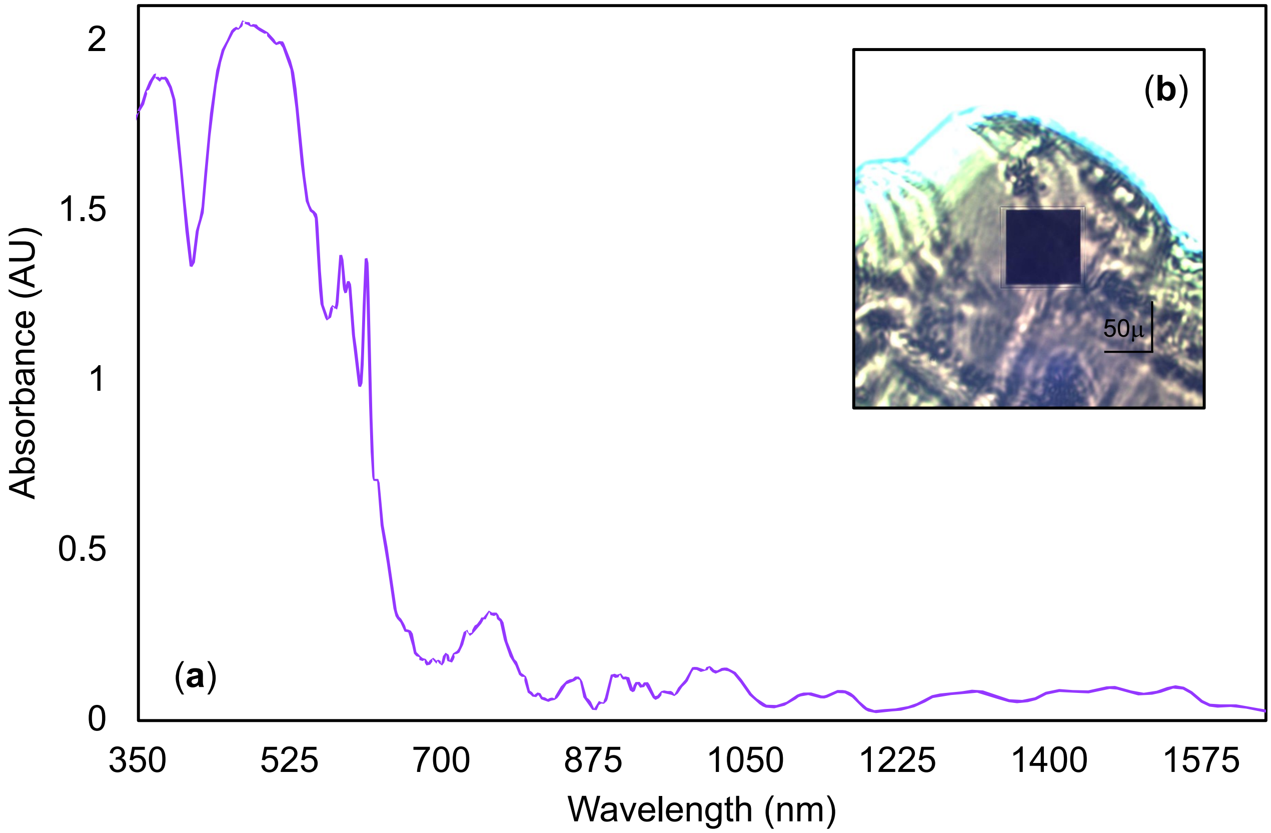

Supplementary Figure 35. Solid-state UV-Vis-NIR spectrum of complex **5-Pu**. (**a**) The spectrum was recorded between 350 to 1700 nm as (**b**) single crystals (Objective = 50X and Aperture = 2). ***Note**: We were unable to obtain the solution-state absorption spectra for **5-Pu** due to the limited amount of material from this one-time isolation.*

S5. Computational details

The DFT calculations were carried out by employing hybrid functional (B3PW91)[3] along with small core pseudopotential Stuttgart basis set[4,5] for uranium, plutonium, and sulfur atoms (polarization functions[6] were added for sulfur atoms), and Pople basis set[7-9] (6-31G**) for the rest of the atoms. For the TDDFT, 2000 states were computed. Frequency calculations were performed to locate minima for the optimized structures and for obtaining thermal corrections over the energies. All the calculations were performed using Gaussian 09 suite of programs.[10] The default settings, the same as in G16, were used. The gradient convergence is 10-5, the SCF convergence is 10-6 and an ultrafine grid was used for the integration.

S5.1. Computational TDDFT details for complex, $[Pu^{III}(Cp^{Me4})_3]$ 1-Pu.

1-Pu (s = 5/2). 120 (AMO-HOMO), 121 (AMO-LUMO)

Supplementary Table 2. Description of the main electron excitations involved in the main transitions observed in the UV-Vis-NIR spectrum for complex **1-Pu**.

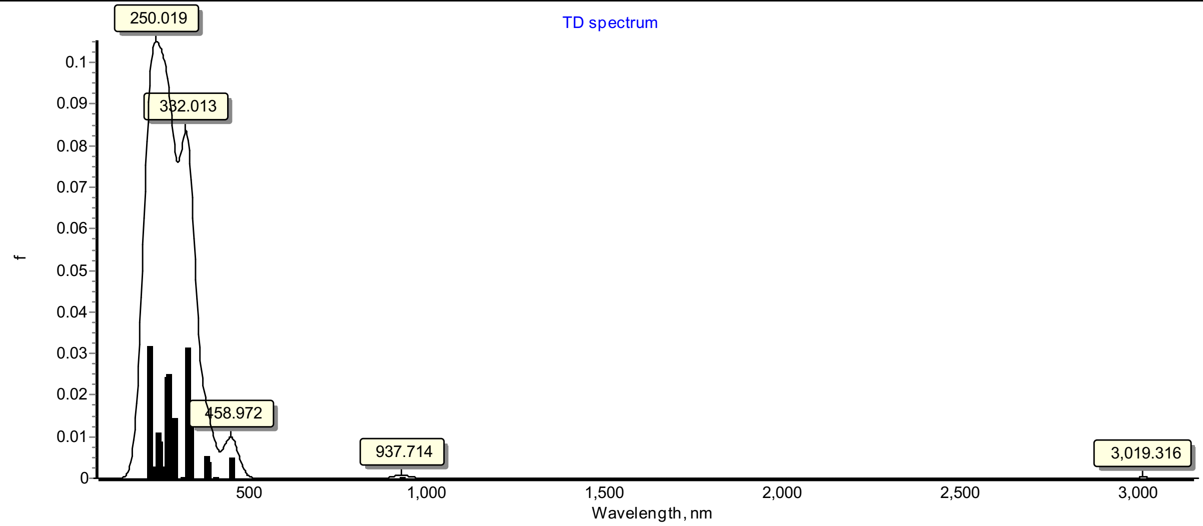

Excited State 3: 6.016-?Sym 0.4101 eV<br>3023.39 nm f=0.0001 <S**2>=8.799<br>112A ->121A 0.34080<br>116A ->122A -0.15094<br>116A ->123A 0.13036<br>119A ->121A -0.62362<br>**120A ->121A 0.66609**	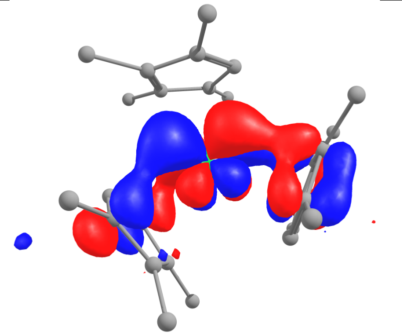	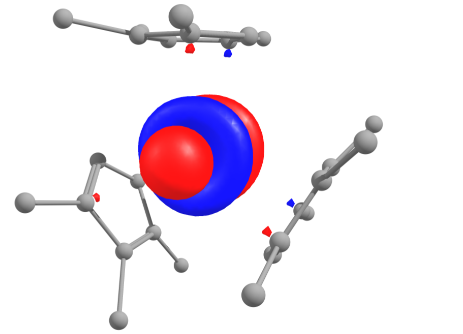
Excited State 10: 6.008-?Sym 1.3222 eV<br>937.73 nm f=0.0004 <S**2>=8.775<br>111A ->122A -0.25407<br>111A ->123A 0.23192<br>**115A ->122A 0.61675**<br>115A ->123A -0.56288<br>116A ->122A -0.25853<br>116A ->123A 0.23660<br>119A ->121A 0.15653<br>120A ->121A 0.17123	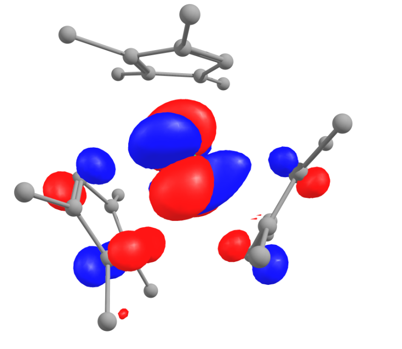	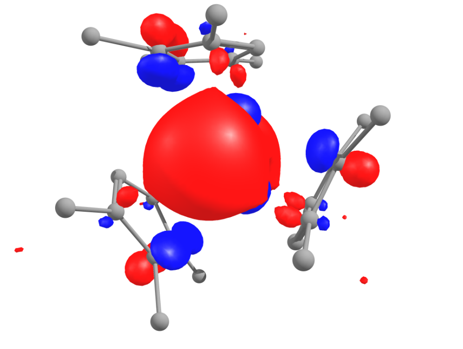

<table>
<tr>
<td rowspan="2">Excited State 11: 6.037-?Sym 2.1529 eV
575.90 nm f=0.0080 <S**2>=8.862
112A ->122A 0.12520
112A ->123A 0.14310
119A ->122A -0.17082
119A ->123A -0.19787
120A ->122A 0.63164
120A ->123A 0.68392</td>
<td rowspan="2">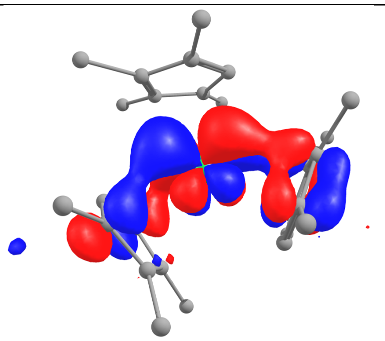</td>
<td>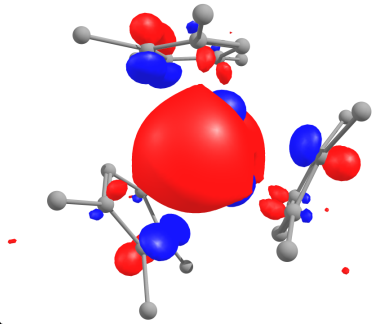
122A</td>
</tr>
<tr>
<td>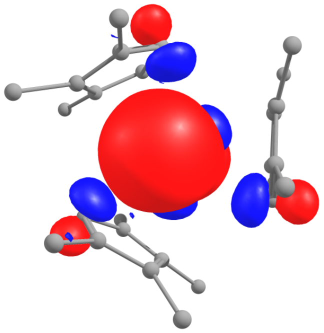
123A</td>
</tr>
</table>

S5.2. Computational TDDFT details for complex, [$Pu^{III}(Cp^{Me4})_2(SPh)_2$] 2-Pu.

2-Pu (s = 5). 220-HOMO(AMO), 221-LUMO(AMO)

Supplementary Table 3. Description of the main electron excitations involved in the main transitions observed in the UV-Vis-NIR spectrum for complex **2-Pu**.

<table>
<tr><td colspan="3">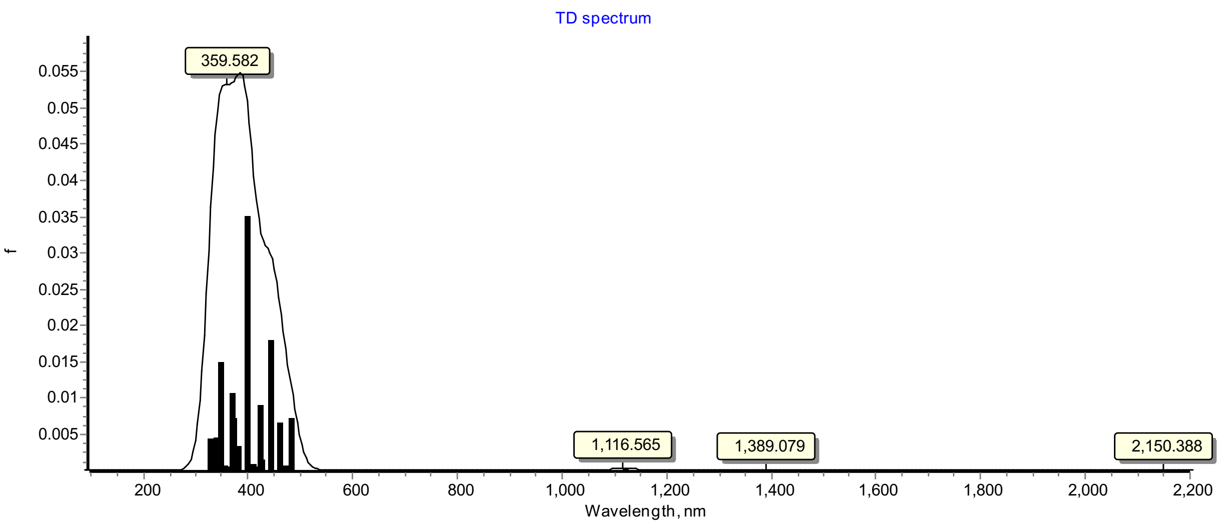
</td></tr>
<tr><td>Excited State 14: 1.011-?Sym 0.5774 eV 2147.40 nm f=0.0000 <S**2>=30.061
201A -> 221A -0.56289
202A -> 224A 0.17795
203A -> 224A 0.61132
206A -> 221A -0.21966
207A -> 224A -0.17893
208A -> 221A -0.15507
209A -> 221A -0.34086
210A -> 224A 0.19558
201A <- 221A -0.12813
203A <- 224A 0.13801</td><td>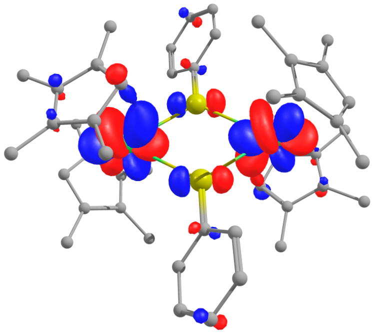</td><td>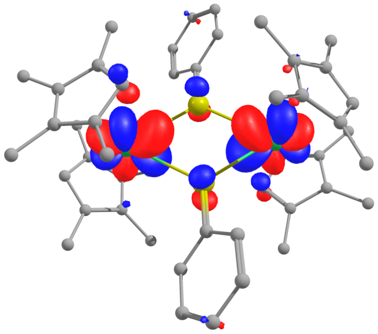</td></tr>
<tr><td>Excited State 15: 11.012-?Sym 0.8922 eV 1389.59 nm f=0.0001 <S**2>=30.066
201A -> 224A -0.15430
202A -> 221A 0.14600
209A -> 224A 0.32062
210A -> 221A -0.10664
211A -> 224A -0.48934
213A -> 221A 0.65212
213A -> 222A 0.10613
214A -> 224A 0.12155
217A -> 222A 0.20682
220A -> 223A 0.22308</td><td>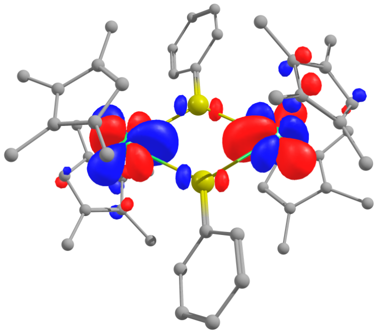</td><td>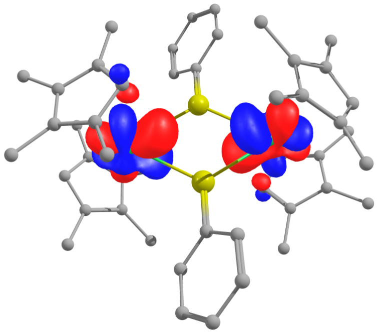</td></tr>
</table>

Excited State 19: 11.012-?Sym 1.1090 eV 1117.94 nm f=0.0003 <S**2>=30.066<br>201A -> 224A -0.13347<br>203A -> 221A 0.14177<br>206A -> 224A 0.56926<br>**207A -> 221A 0.61413**<br>207A -> 222A 0.10491<br>209A -> 224A -0.11474<br>212A -> 222A 0.12223<br>214A -> 224A -0.21514<br>215A -> 223A 0.10829<br>216A -> 221A -0.22099<br>218A -> 223A -0.19519	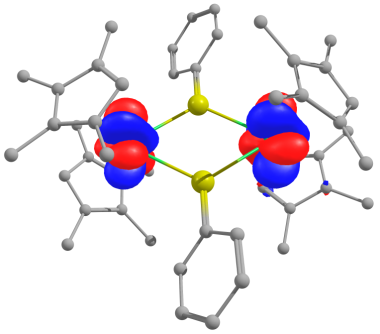	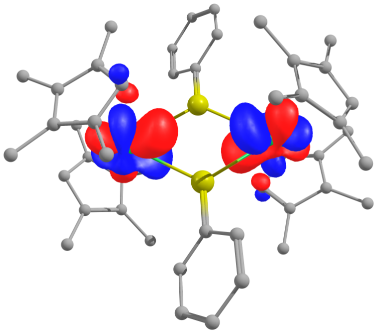
Excited State 21: 11.050-?Sym 2.2214 eV 558.13 nm f=0.0000 <S**2>=30.274<br>204A -> 225A -0.14620<br>208A -> 225A -0.12178<br>217A -> 226A -0.26148<br>219A -> 226A -0.17844<br>220A -> 221A -0.17984<br>**220A -> 225A 0.89185**	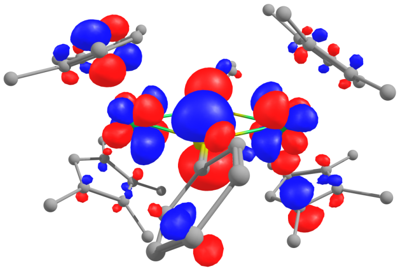	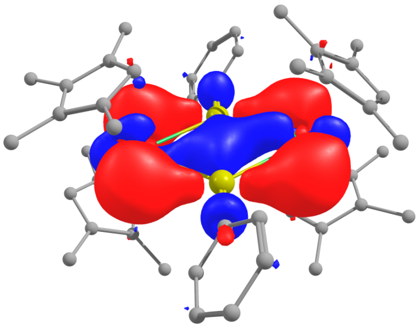
Excited State 22: 11.033-?Sym 2.4154 eV 513.30 nm f=0.0001 <S**2>=30.183<br>205A -> 225A 0.16120<br>208A -> 226A 0.13444<br>212A -> 225A 0.19216<br>217A -> 225A 0.41118<br>218A -> 226A 0.15508<br>219A -> 221A -0.13932<br>**219A -> 225A 0.69021**<br>220A -> 226A -0.41400	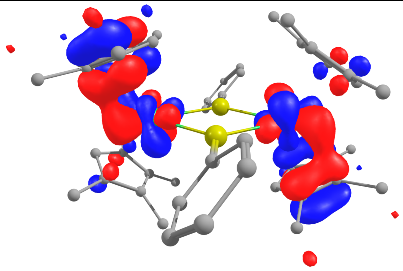	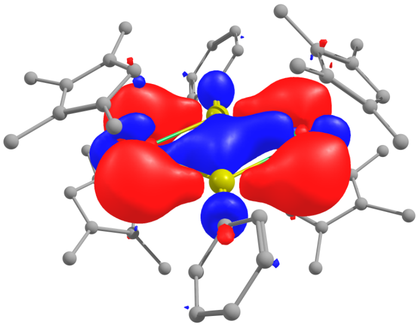
Excited State 25: 11.199-?Sym 2.5859 eV 479.46 nm f=0.0071 <S**2>=31.103<br>208A -> 223A -0.10492<br>212A -> 222A 0.46784<br>213A -> 225A 0.10367<br>214A -> 224A -0.20742<br>**215A -> 223A 0.51462**<br>216A -> 221A -0.31407<br>217A -> 222A 0.25449<br>218A -> 223A 0.28431<br>219A -> 222A -0.25097<br>220A -> 223A -0.28313	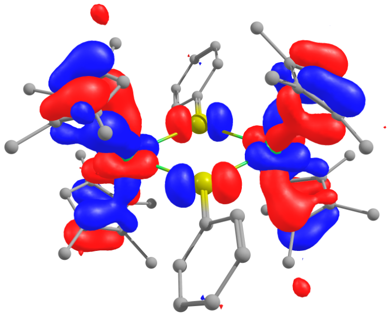	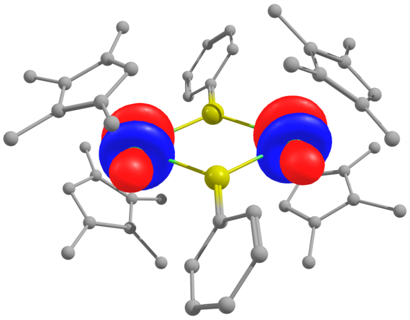

Excited State 35: 11.064-?Sym<br>2.8014 eV 442.58 nm f=0.0179<br><S**2>=30.354<br>209A -> 226A -0.17224<br>210A -> 225A -0.10576<br>211A -> 226A 0.29737<br>213A -> 221A -0.11032<br>**213A -> 225A 0.75940**<br>214A -> 224A 0.21280<br>216A -> 221A 0.36969	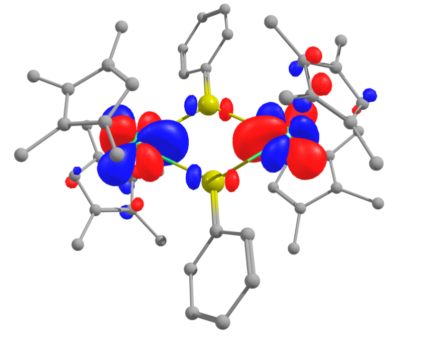	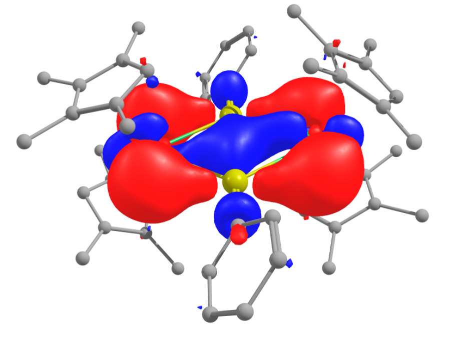
Excited State 39: 11.143-?Sym<br>2.9488 eV 420.46 nm f=0.0090<br><S**2>=30.792<br>**204A -> 223A 0.49153**<br>205A -> 222A -0.48523<br>206A -> 226A -0.19054<br>207A -> 225A 0.35139<br>210A -> 221A -0.10845<br>214A -> 226A 0.12445<br>216A -> 225A -0.31237<br>217A -> 222A 0.28123<br>218A -> 223A 0.16423<br>219A -> 222A 0.25261	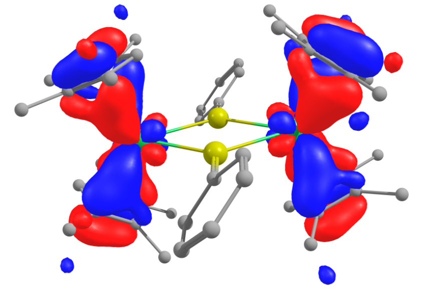	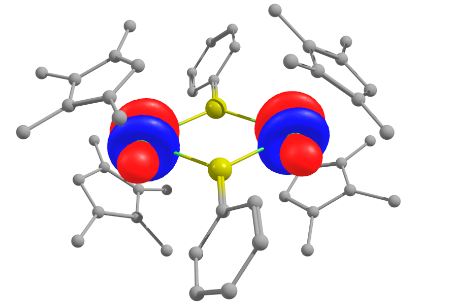
Excited State 51: 11.204-?Sym<br>3.1274 eV 396.44 nm f=0.0352<br><S**2>=31.130<br>201A -> 224A 0.27009<br>202A -> 221A -0.16653<br>203A -> 221A -0.17840<br>208A -> 224A -0.10461<br>209A -> 224A -0.32202<br>**210A -> 221A 0.71329**<br>210A -> 222A 0.12018<br>211A -> 224A -0.37595<br>214A -> 224A 0.13672	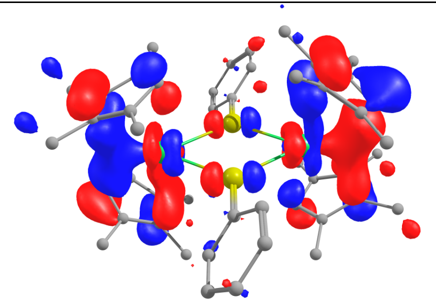	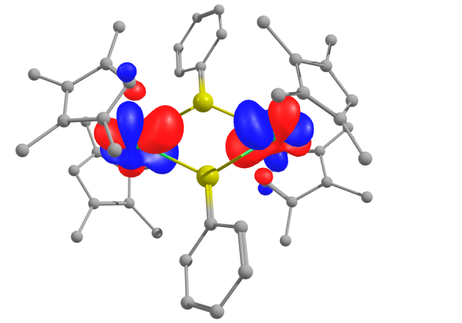

S5.3. Computational TDDFT details for complex, [$U^{III}(Cp^{Me4})_3$(SPh)] 3-U.

(s = 1).

Supplementary Table 4. Description of the main electron excitations involved in the main transitions observed in the UV-Vis-NIR spectrum for complex **3-U**.

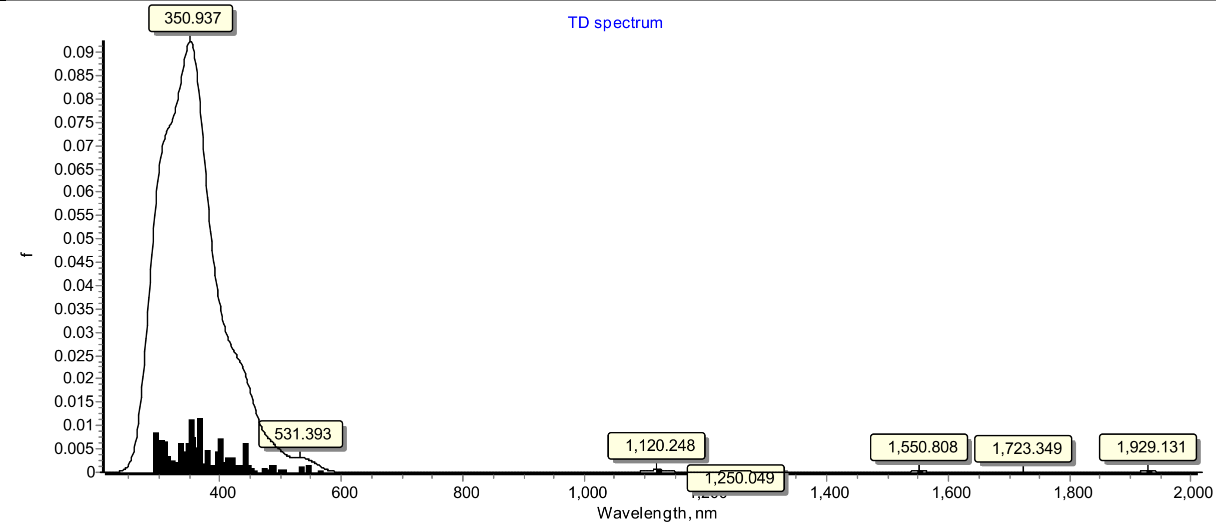

Excited State 6: 3.012-?Sym 0.6426 eV 1929.40 nm f=0.0002 <S**2>=2.019<br>137A ->143A -0.16517<br>137A ->144A 0.24146<br>**138A ->143A 0.67331**<br>138A ->144A -0.20212<br>138A ->145A -0.13182<br>138A ->146A 0.13488<br>139A ->143A 0.15752<br>140A ->144A -0.30329<br>140A ->146A -0.16512<br>141A ->143A 0.40333<br>141A ->144A -0.12194	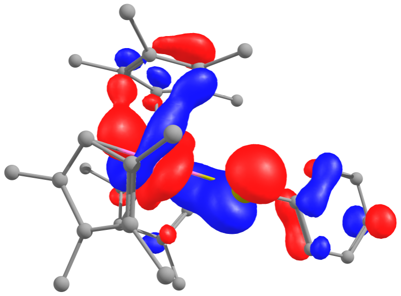	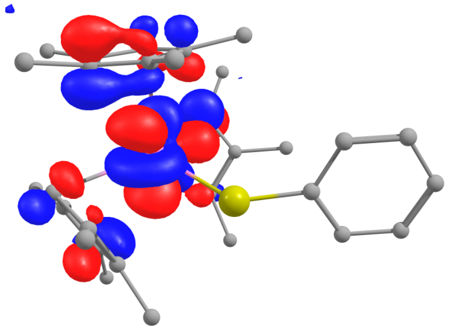
Excited State 7: 3.008-?Sym 0.7192 eV 1723.91 nm f=0.0001 <S**2>=2.013<br>134A ->143A -0.14564<br>134A ->145A -0.10337<br>137A ->143A -0.39485<br>137A ->144A -0.15055<br>137A ->145A -0.21908<br>138A ->142A 0.11156<br>138A ->144A 0.19287<br>138A ->145A -0.10904<br>138A ->146A 0.17309<br>**140A ->143A 0.60368**<br>140A ->144A 0.19014	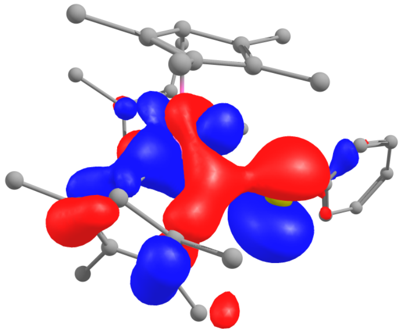	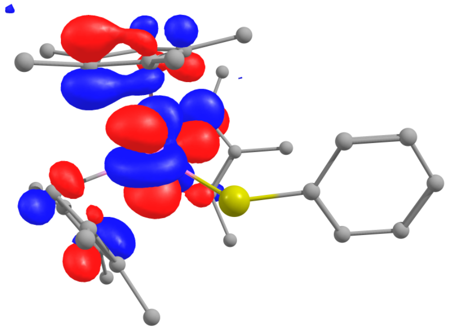

140A ->145A 0.41341<br>141A ->144A 0.10156<br>141A ->146A 0.11976		
Excited State 8: 3.016-?Sym 0.7993 eV 1551.14 nm f=0.0002 <S**2>=2.024<br>134A ->146A -0.12958<br>135A ->146A -0.11565<br>137A ->145A -0.21746<br>137A ->146A -0.22987<br>138A ->143A 0.32288<br>138A ->144A 0.13577<br>138A ->145A 0.24322<br>138A ->146A -0.37863<br>139A ->143A 0.10954<br>139A ->146A -0.17666<br>140A ->145A 0.26198<br>**140A ->146A 0.52145**<br>141A ->143A 0.20126<br>141A ->145A 0.12065<br>141A ->146A -0.24585	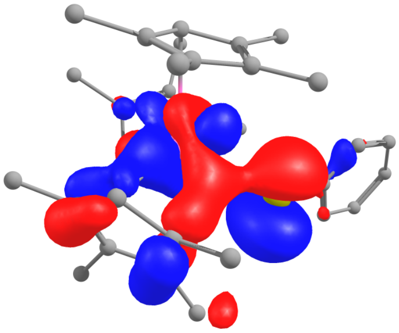	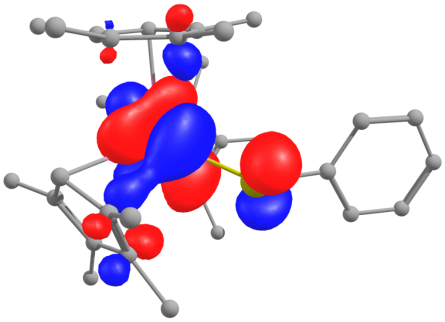
Excited State 9: 3.018-?Sym 0.9920 eV 1249.88 nm f=0.0004 <S**2>=2.027<br>137A ->143A -0.18899<br>137A ->144A 0.30749<br>138A ->143A -0.23955<br>138A ->144A -0.41272<br>138A ->145A 0.10784<br>138A ->146A -0.33883<br>139A ->143A -0.12489<br>139A ->146A -0.10762<br>**140A ->143A 0.41818**<br>140A ->144A -0.35166<br>141A ->143A -0.17049<br>141A ->144A -0.21664<br>141A ->146A -0.21280	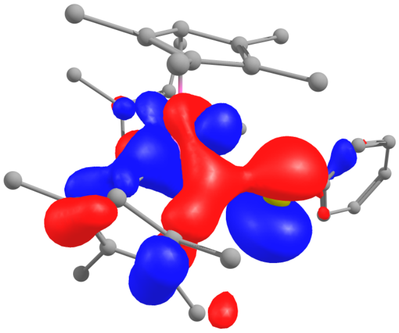	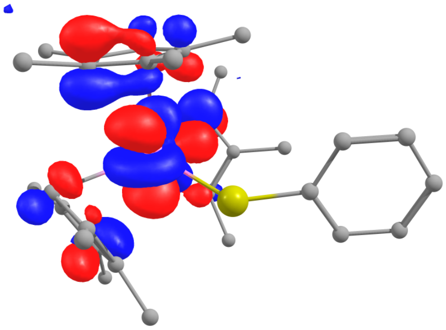

Excited State 10: 3.019-?Sym<br>1.1061 eV 1120.90 nm f=0.0005<br><S**2>=2.029<br>137A ->143A -0.14762<br>137A ->144A -0.20518<br>137A ->146A 0.27677<br>138A ->143A 0.18465<br>**138A ->145A 0.48696**<br>138A ->146A -0.21824<br>139A ->145A 0.15570<br>140A ->143A 0.16241<br>140A ->144A 0.25227<br>140A ->145A -0.28176<br>140A ->146A -0.38267<br>141A ->143A 0.11054<br>141A ->145A 0.29873	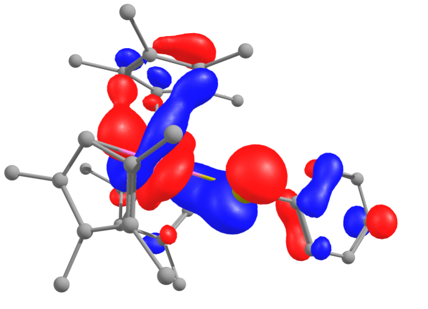	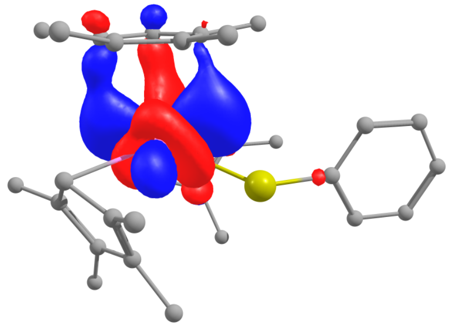
Excited State 11: 3.664-?Sym<br>1.8167 eV 682.46 nm f=0.0000<br><S**2>=3.107<br>138A ->142A -0.14012<br>**139A ->142A 0.94447**<br>141A ->142A -0.24402	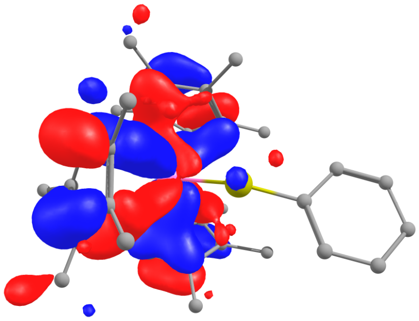	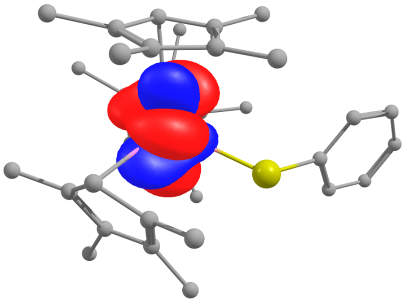
Excited State 12: 3.656-?Sym<br>2.2030 eV 562.79 nm f=0.0002<br><S**2>=3.092<br>135A ->142A 0.13292<br>137A ->142A -0.54776<br>138A ->142A -0.49569<br>140A ->142A -0.27176<br>**141A ->142A 0.58479**	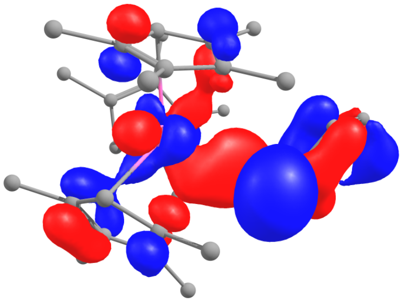	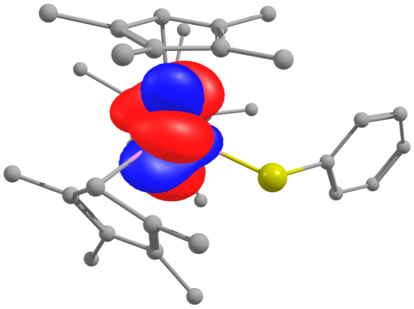
Excited State 13: 3.676-?Sym<br>2.2781 eV 544.23 nm f=0.0015<br><S**2>=3.128<br>135A ->142A 0.18921<br>137A ->142A 0.57588<br>138A ->142A -0.21326<br>139A ->142A 0.10718<br>139A ->143A 0.30794<br>140A ->142A 0.41519<br>**141A ->142A 0.51591**	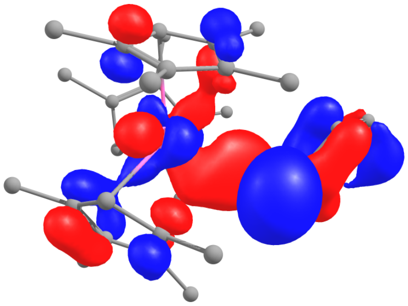	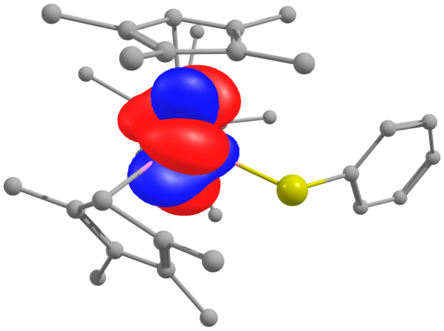

Excited State 14: 3.880-?Sym<br>2.3330 eV 531.43 nm f=0.0014<br><S**2>=3.513<br>137A ->142A -0.21885<br>**139A ->143A 0.79603**<br>140A ->142A -0.15428<br>141A ->142A -0.17477<br>141A ->143A -0.28458<br>138B ->144B -0.25663<br>139B ->144B 0.16458	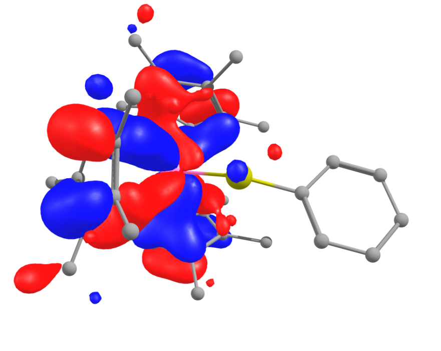	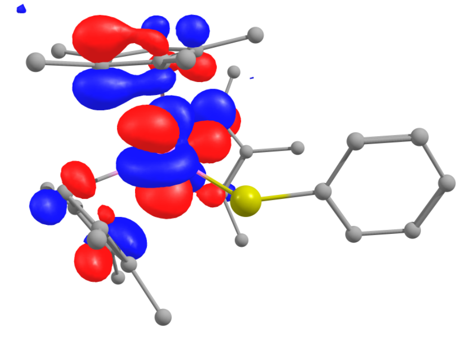
Excited State 15: 3.667-?Sym<br>2.4674 eV 502.48 nm f=0.0006<br><S**2>=3.112<br>**136A ->142A 0.95472**<br>139A ->144A -0.15869<br>139A ->146A -0.10259	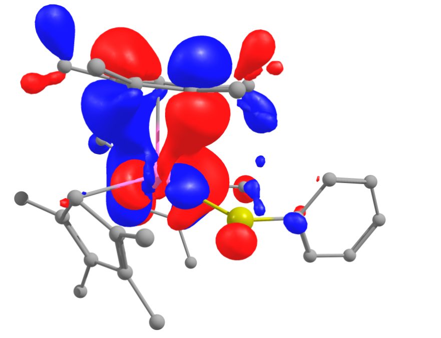	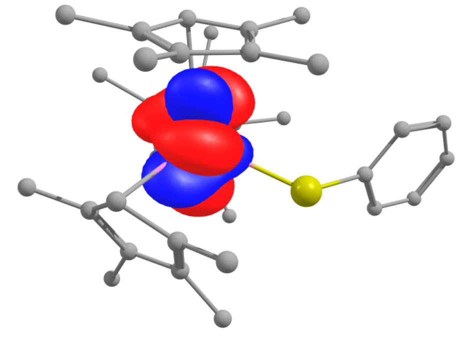
Excited State 16: 3.743-?Sym<br>2.4858 eV 498.78 nm f=0.0007<br><S**2>=3.252<br>136A ->142A 0.18437<br>**139A ->144A 0.91370**<br>141A ->144A -0.25186	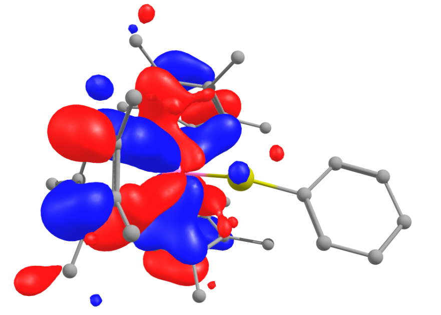	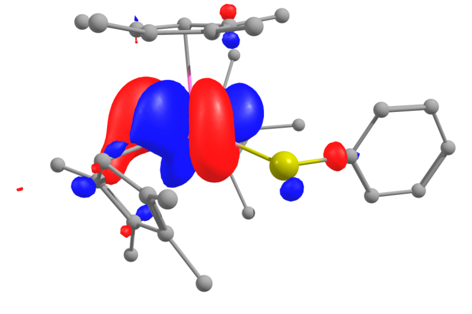
Excited State 17: 3.738-?Sym<br>2.5499 eV 486.23 nm f=0.0016<br><S**2>=3.244<br>135A ->143A -0.10773<br>136A ->142A -0.10843<br>**137A ->143A 0.48262**<br>**138A ->143A 0.43369**<br>139A ->143A -0.10322<br>139A ->145A -0.23230<br>139A ->146A -0.36623<br>140A ->143A 0.23507<br>141A ->143A -0.46325	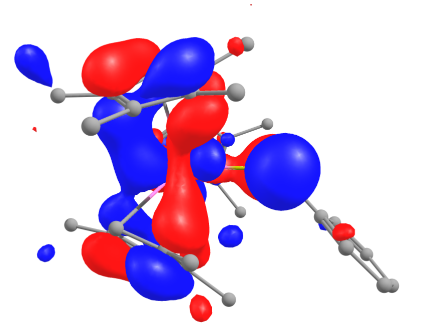<br>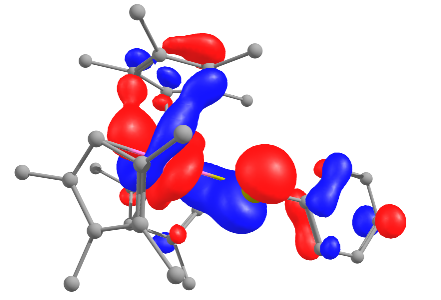	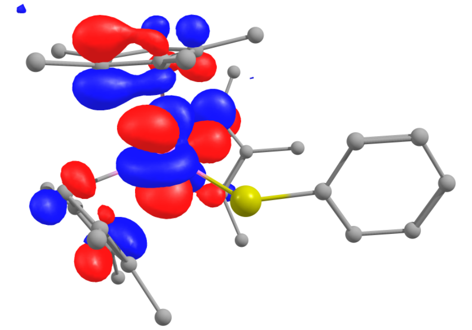

<table>
<tr>
<td>Excited State 18: 3.742-?Sym 2.5670 eV 482.99 nm f=0.0015 <S**2>=3.250
135A ->142A 0.13641
138A ->143A 0.18059
138A ->145A -0.10746
139A ->145A 0.85366
141A ->143A -0.25884
141A ->145A -0.23960</td>
<td>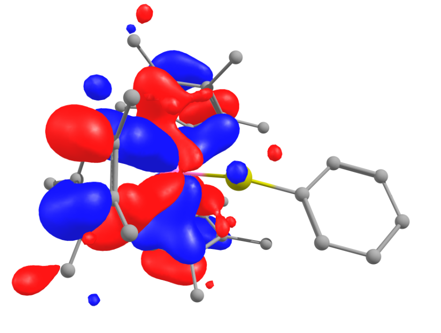</td>
<td>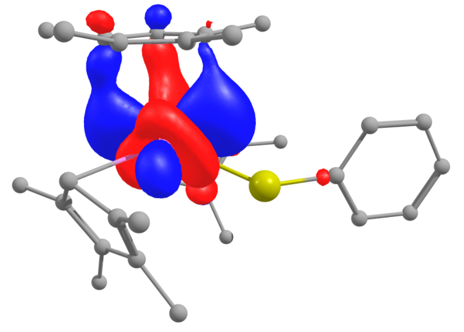</td>
</tr>
<tr>
<td>Excited State 26:3.776-?Sym 2.8143 eV 440.55 nm f=0.0062 <S**2>=3.315
134A ->142A -0.11947
135A ->143A -0.10647
136A ->143A -0.27113
137A ->145A 0.26835
137A ->146A -0.15941
138A ->145A 0.22392
138A ->146A -0.44092
139A ->146A 0.16408
140A ->145A 0.13443
141A ->145A -0.25484
141A ->146A 0.59631</td>
<td>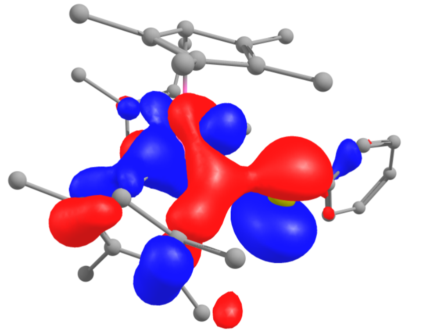</td>
<td>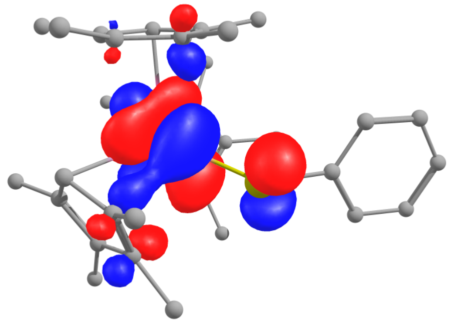</td>
</tr>
<tr>
<td>Excited State 30: 3.747-?Sym 2.8954 eV 428.21 nm f=0.0017 <S**2>=3.260
135A ->143A 0.11550
135A ->144A -0.12819
137A ->145A 0.66708
138A ->145A -0.12735
139A ->145A 0.10792
140A ->145A 0.47420
141A ->145A 0.43189</td>
<td>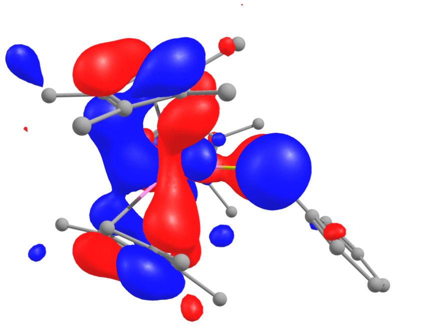</td>
<td>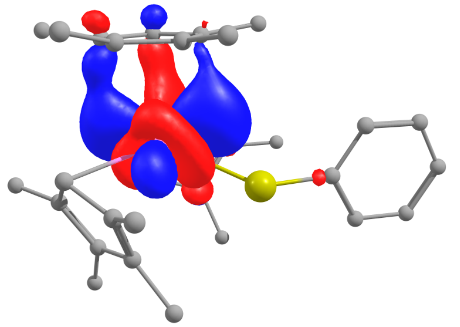</td>
</tr>
<tr>
<td>Excited State 31: 3.685-?Sym 2.9691 eV 417.59 nm f=0.0031 <S**2>=3.145
134A ->143A 0.18186
135A ->143A 0.85371
136A ->144A -0.25463
137A ->146A 0.10697
139A ->143A -0.12639
141A ->143A -0.22219
141A ->146A 0.10889</td>
<td>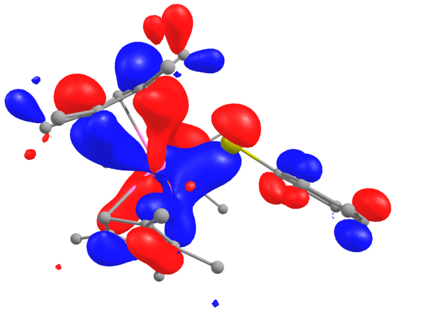</td>
<td>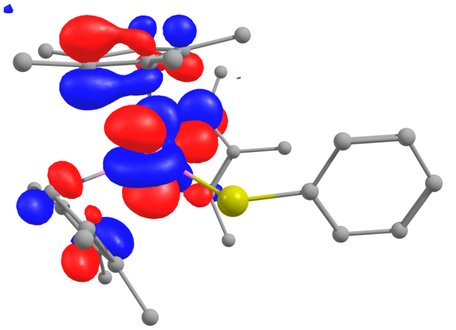</td>
</tr>
</table>

<table>
<tr>
<td>Excited State 51: 3.732-?Sym 3.3886 eV 365.89 nm f=0.0095 <S**2>=3.232
130A ->147A -0.10606
131A ->148A 0.19593
133A ->143A -0.28045
134A ->146A -0.10092
135A ->147A -0.11873
138A ->147A -0.12720
141A ->147A 0.27125
130B ->147B 0.11674
131B ->148B -0.19673
135B ->140B 0.14218
135B ->147B 0.11634
136B ->140B 0.12290
137B ->141B 0.37855
137B ->143B -0.19051
138B ->141B 0.19833
138B ->143B -0.28503
138B ->147B -0.16695
139B ->141B 0.19628
139B ->142B 0.17027
139B ->143B -0.27500
139B ->147B -0.23308</td>
<td>141A
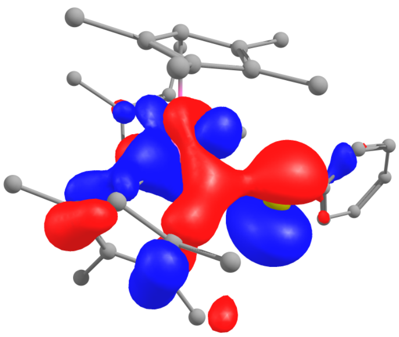
137B
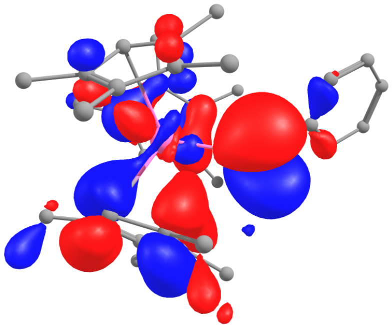</td>
<td>141B
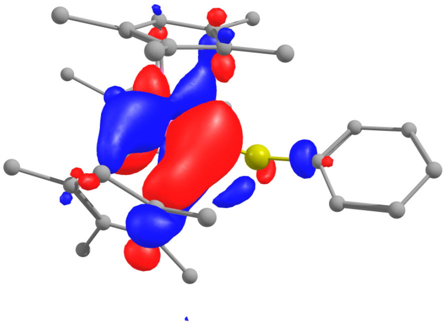</td>
</tr>
</table>

S5.4. Computational TDDFT details for complex, [U^{III}(Cp^{Me4})$_3$(NHPh)] 4-U.

(s = 1). 142 (AMO-HOMO), 143 (AMO-LUMO)

Supplementary Table 5. Description of the main electron excitations involved in the main transitions observed in the UV-Vis-NIR spectrum for complex **4-U**.

<table>
<tr><td colspan="3">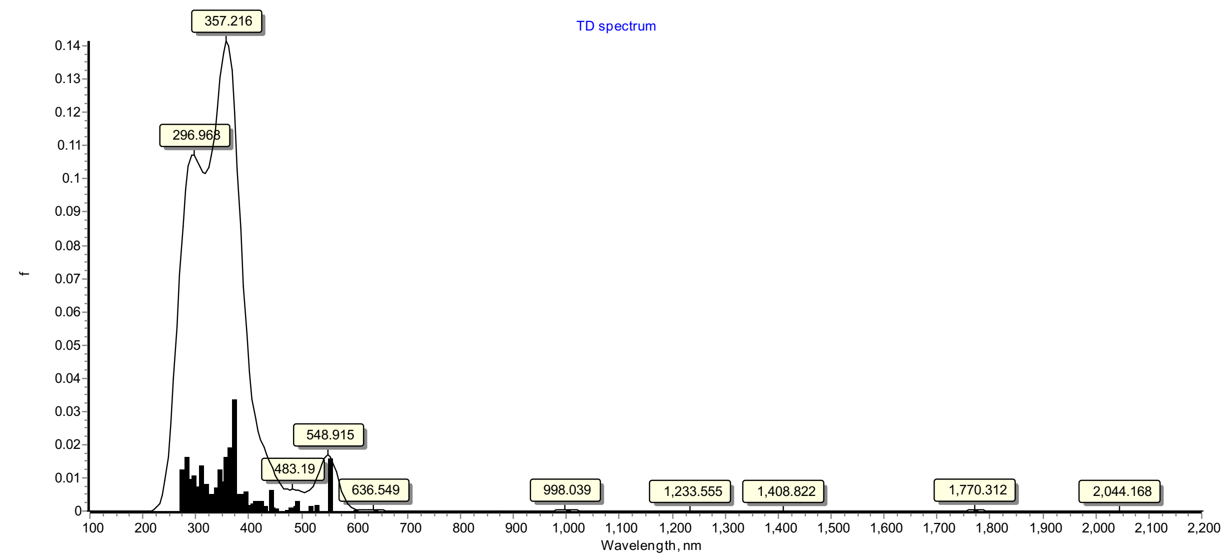
</td></tr>
<tr><td>Excited State 6: 3.012-?Sym 0.6062 eV 2045.25 nm f=0.0001 <S**2>=2.018
138A ->145A 0.11787
139A ->145A 0.47608
139A ->146A -0.16231
140A ->144A -0.19241
140A ->145A 0.12286
140A ->146A 0.30264
140A ->147A 0.24871
141A ->145A 0.63211
141A ->146A -0.18484
141A ->147A 0.10034</td><td>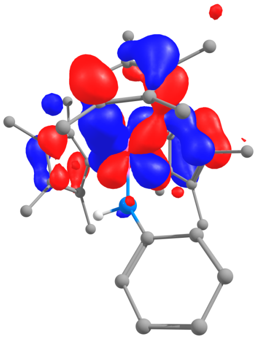</td><td>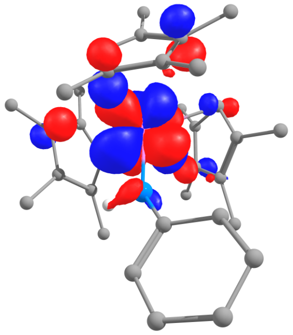</td></tr>
<tr><td>Excited State 7: 3.012-?Sym 0.6999 eV 1771.53 nm f=0.0003 <S**2>=2.019
136A ->147A -0.10892
138A ->147A 0.14771
139A ->144A -0.31130
139A ->145A -0.12358
140A ->145A -0.17592
140A ->146A 0.20602
140A ->147A 0.71906
140A ->150A -0.12676
141A ->144A -0.39502
141A ->145A -0.17127
142A ->147A 0.17605</td><td>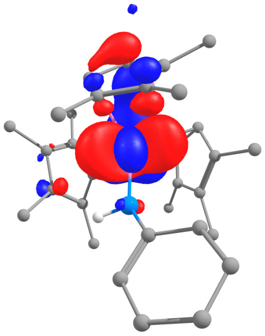</td><td>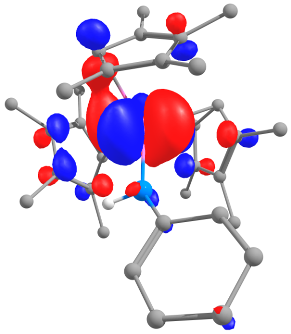</td></tr>
</table>

140A <-147A 0.13877		
Excited State 8: 3.009-?Sym 0.8815 eV 1406.56 nm f=0.0001 <S**2>=2.014<br>138A ->145A 0.15702<br>139A ->144A -0.30411<br>140A ->144A 0.14626<br>**140A ->145A 0.76373**<br>140A ->146A -0.14515<br>141A ->144A -0.37720<br>141A ->145A 0.10410<br>142A ->145A 0.17219	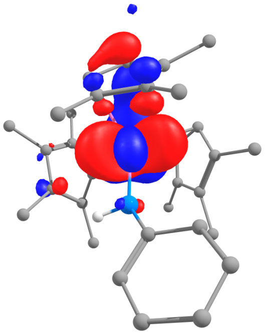	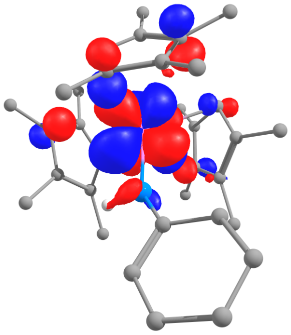
Excited State 9: 3.015-?Sym 1.0072 eV 1230.96 nm f=0.0001 <S**2>=2.022<br>136A ->146A -0.11141<br>138A ->146A 0.13793<br>139A ->144A -0.12071<br>139A ->145A -0.13477<br>139A ->147A 0.16832<br>**140A ->146A 0.73072**<br>140A ->147A -0.38505<br>140A ->149A -0.11724<br>141A ->144A -0.16640<br>141A ->145A -0.19803<br>141A ->147A 0.19906<br>142A ->146A 0.18543<br>142A ->147A -0.11638	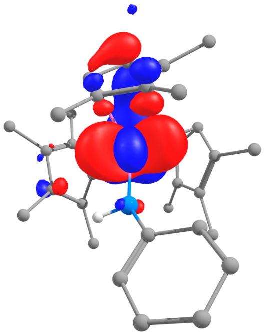	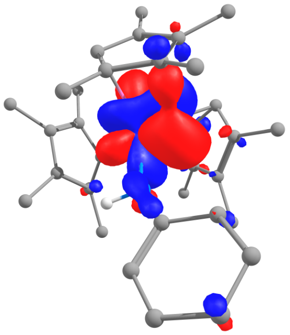

Excited State 10: 3.017-?Sym<br>1.2392 eV 1000.53 nm f=0.0004<br><S**2>=2.025<br>138A ->147A 0.12262<br>139A ->146A 0.10119<br>139A ->147A 0.52910<br>140A ->144A -0.13939<br>140A ->145A 0.11724<br>140A ->146A -0.23794<br>140A ->147A 0.12728<br>141A ->146A 0.14235<br>**141A ->147A 0.69860**<br>141A ->150A -0.10673	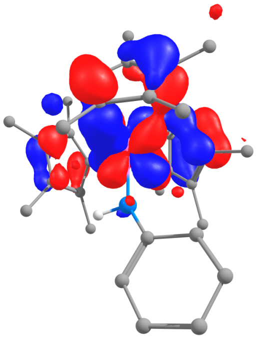	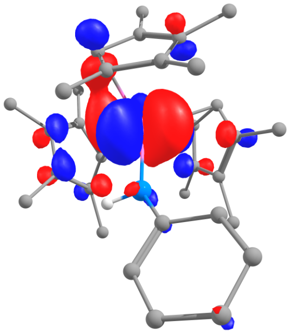
Excited State 11: 3.655-?Sym<br>1.9436 eV 637.91 nm f=0.0003<br><S**2>=3.089<br>136A ->143A -0.15835<br>139A ->143A -0.17542<br>140A ->143A -0.24678<br>141A ->143A 0.30046<br>**142A ->143A 0.88230**	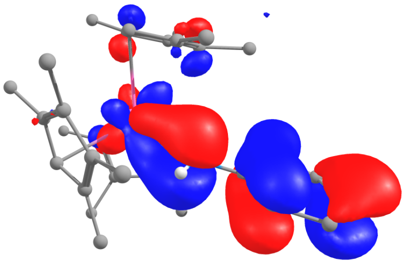	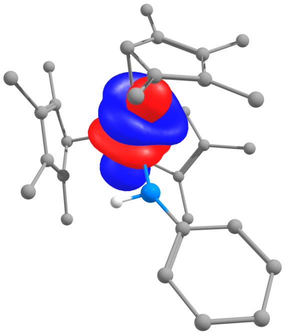
Excited State 13: 3.866-?Sym<br>2.2488 eV 551.34 nm f=0.0160<br><S**2>=3.486<br>139A ->145A 0.11412<br>140A ->144A -0.20656<br>141A ->144A 0.17241<br>**142A ->144A 0.87103**<br>142A ->145A 0.10825<br>140B ->142B 0.14721<br>140B ->143B -0.13442<br>140B ->144B 0.20592	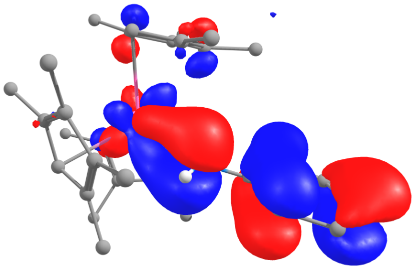	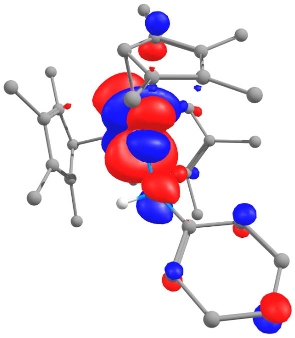
Excited State 22: 3.733-?Sym<br>2.8143 eV 440.56 nm f=0.0062<br><S**2>=3.233<br>136A ->143A -0.10786<br>137A ->145A -0.10343<br>**138A ->144A 0.80556**<br>138A ->145A -0.13793<br>139A ->144A -0.13836<br>140A ->144A -0.16480<br>140A ->147A 0.11849<br>142A ->147A -0.43998<br>138B ->142B 0.10369	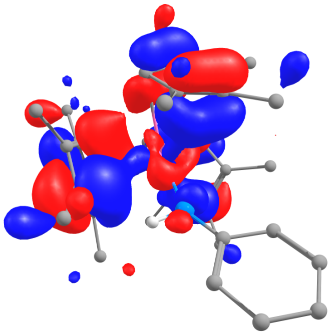	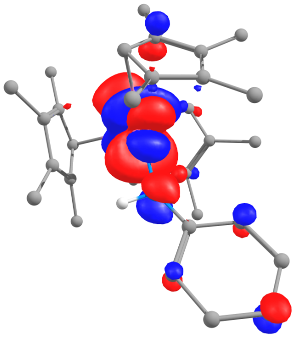

Excited State 30: 3.697-?Sym<br>3.0501 eV 406.49 nm f=0.0021<br><S**2>=3.168<br>135A ->143A -0.17720<br>**136A ->144A 0.83050**<br>137A ->145A 0.22982<br>142A ->144A 0.14126<br>142A ->147A -0.11746<br>136B ->142B 0.12717<br>140B ->142B -0.28925	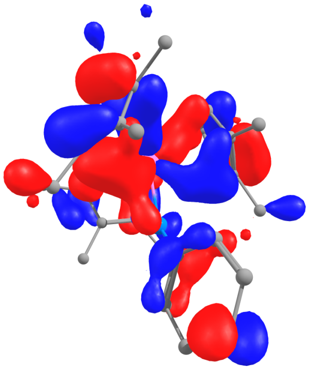	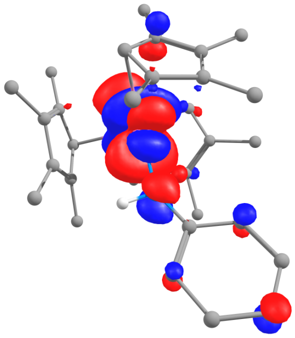
Excited State 31: 3.685-?Sym<br>3.1160 eV 397.90 nm f=0.0009<br><S**2>=3.144<br>**135A ->143A 0.92544**<br>139B ->142B -0.11787<br>140B ->142B -0.22247	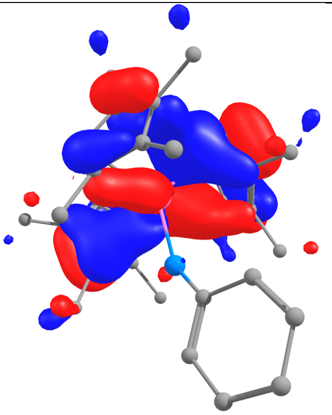	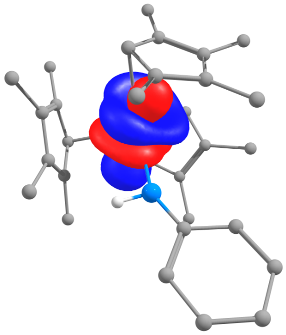
Excited State 35: 3.743-?Sym<br>3.1744 eV 390.57 nm f=0.0051<br><S**2>=3.252<br>134A ->143A 0.32568<br>136A ->145A 0.60802<br>137A ->146A -0.21296<br>**138A ->146A 0.57449**<br>140A ->146A -0.13431	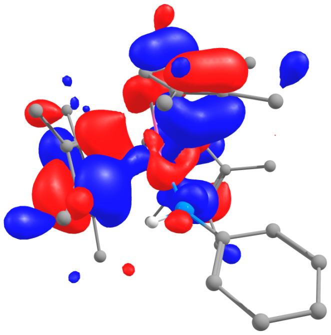	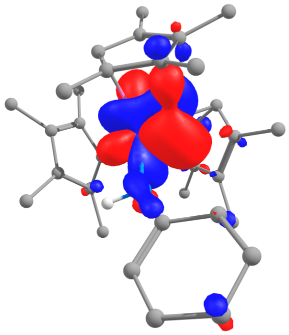
Excited State 59: 3.712-?Sym<br>3.7244 eV 332.90 nm f=0.0006<br><S**2>=3.195<br>133A ->148A 0.16657<br>135A ->146A 0.19595<br>136A ->146A -0.10948<br>**136A ->147A 0.27326**<br>142A ->148A -0.16471<br>**142A ->149A 0.27810**<br>142A ->153A -0.11411<br>133B ->148B 0.17463<br>136B ->142B -0.27971<br>136B ->144B -0.12706<br>138B ->143B 0.27067<br>138B ->144B 0.29944<br>139B ->146B -0.22291	142A<br>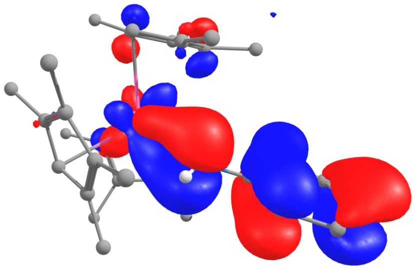<br>136A	149A<br>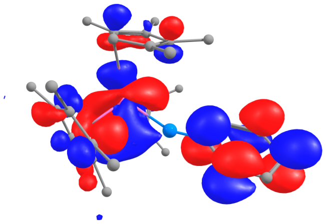<br>147A

140B ->143B -0.12237<br>140B ->146B 0.20232<br>140B ->147B -0.14897<br>140B ->148B 0.21461<br>140B ->149B 0.26169	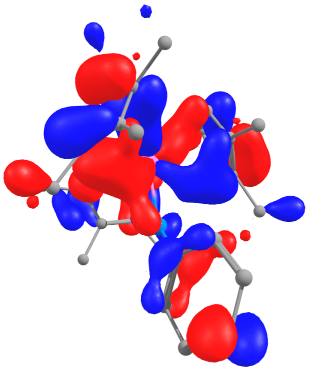	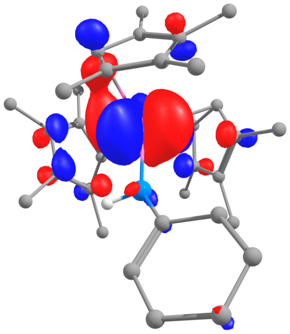

S5.5. Supplementary computed reaction profiles

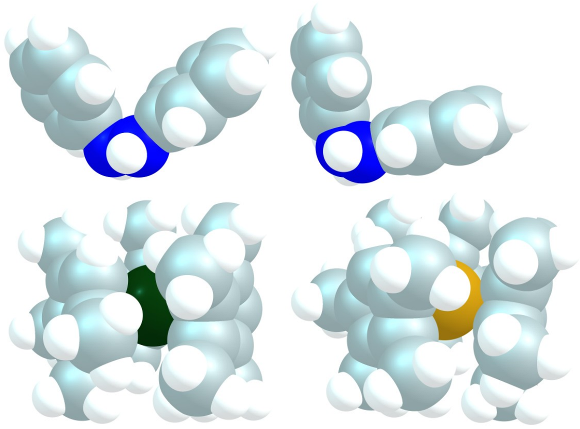

Supplementary Figure 36. 3D representation using big atoms model for the substrate coordination in (left) ^{U}**Int1** and (right) ^{Pu}**Int1**, for the computed reactivity of **1-U** and **1-Pu** with $(PhHN)_2$.

S6. References

1 Evans, W. J., Kozimor, S. A., Ziller, J. W., Fagin, A. A. & Bochkarev, M. N. Facile Syntheses of Unsolvated UI3 and Tetramethylcyclopentadienyl Uranium Halides. *Inorg. Chem.* **44**, 3993-4000 (2005). https://doi.org/10.1021/ic0482685

2 Lukens, W. W., Beshouri, S. M., Blosch, L. L. & Andersen, R. A. Oxidative Elimination of H2 from [Cp'2U(μ-OH)]2 To Form [Cp'2U(μ-O)]2, Where Cp' Is 1,3-(Me3C)2C5H3 or 1,3-(Me3Si)2C5H3. *J. Am. Chem. Soc.* **118**, 901-902 (1996). https://doi.org/10.1021/ja9531283

3 Becke, A. D. Density-functional thermochemistry. III. The role of exact exchange. *The Journal of Chemical Physics* **98**, 5648-5652 (1993). https://doi.org/10.1063/1.464913

4 Moritz, A., Cao, X. & Dolg, M. Quasirelativistic energy-consistent 5f-in-core pseudopotentials for divalent and tetravalent actinide elements. *Theoretical Chemistry Accounts* **118**, 845-854 (2007). https://doi.org/10.1007/s00214-007-0330-6

5 Bergner, A., Dolg, M., Küchle, W., Stoll, H. & Preuß, H. Ab initio energy-adjusted pseudopotentials for elements of groups 13–17. *Molec. Phys.* **80**, 1431-1441 (1993). https://doi.org/10.1080/00268979300103121

6 Höllwarth, A. *et al.* A set of d-polarization functions for pseudo-potential basis sets of the main group elements Al□Bi and f-type polarization functions for Zn, Cd, Hg. *Chem. Phys. Lett.* **208**, 237-240 (1993). https://doi.org/https://doi.org/10.1016/0009-2614(93)89068-S

7 Ditchfield, R., Hehre, W. J. & Pople, J. A. Self-Consistent Molecular-Orbital Methods. IX. An Extended Gaussian-Type Basis for Molecular-Orbital Studies of Organic Molecules. *The Journal of Chemical Physics* **54**, 724-728 (1971). https://doi.org/10.1063/1.1674902

8 Hehre, W. J., Ditchfield, R. & Pople, J. A. Self—Consistent Molecular Orbital Methods. XII. Further Extensions of Gaussian—Type Basis Sets for Use in Molecular Orbital Studies of Organic Molecules. *The Journal of Chemical Physics* **56**, 2257-2261 (1972). https://doi.org/10.1063/1.1677527

9 Hariharan, P. C. & Pople, J. A. The influence of polarization functions on molecular orbital hydrogenation energies. *Theoretica chimica acta* **28**, 213-222 (1973). https://doi.org/10.1007/BF00533485

10 Gaussian 16 Rev. C.01 (Wallingford, CT, 2016).
