# Supplementary material for: Plutonium(III) versus uranium(III) and samarium(III) in small molecule activation chemistry
Source: Nat Commun. 2026 May 2;17:5956. doi: 10.1038/s41467-026-72229-7 (PMC13342291; doi:10.1038/s41467-026-72229-7)
Supplement: Supplementary file 3 — Source Data [file 41467_2026_72229_MOESM3_ESM.docx]

**Optimized geometries for the reactivity of 1-Pu and 1-U with (PhS)_2_.**

**1-Pu**

E=-1606.19436496 Ha

Sum of electronic and thermal Enthalpies= -1605.574000 Ha

Sum of electronic and thermal Free Energies= -1605.676667 Ha

Pu 7.839688000 4.529805000 1.354809000

C 6.490413000 2.178634000 1.354756000

C 7.306468000 2.078257000 2.509618000

C 8.645552000 1.890984000 2.066814000

C 8.645476000 1.891074000 0.642437000

C 7.306347000 2.078401000 0.199799000

C 6.840466000 2.001658000 3.936940000

C 9.785045000 1.470081000 2.946355000

C 9.784862000 1.470288000 -0.237300000

C 6.840218000 2.001990000 -1.227492000

H 5.413879000 2.279570000 1.354795000

H 5.766759000 2.186783000 4.011560000

H 7.345905000 2.718060000 4.597921000

H 7.028110000 1.006145000 4.359503000

H 10.758225000 1.803646000 2.573766000

H 9.834247000 0.374202000 3.015331000

H 9.673897000 1.844875000 3.968270000

H 9.833755000 0.374428000 -0.306815000

H 10.758128000 1.803384000 0.135479000

H 9.673850000 1.845611000 -1.259038000

H 7.028083000 1.006616000 -1.650284000

H 7.345398000 2.718661000 -1.888384000

H 5.766455000 2.186855000 -1.301961000

C 10.552623000 4.539652000 1.354622000

C 10.230917000 5.295968000 2.509536000

C 6.479052000 6.872464000 1.354669000

C 5.985731000 6.215040000 2.509549000

C 9.722896000 6.549006000 2.067190000

C 10.532124000 4.929831000 3.935990000

H 10.818525000 3.879512000 4.023270000

H 9.684034000 5.099655000 4.611770000

H 11.365192000 5.527171000 4.329361000

C 9.722791000 6.549113000 0.642475000

C 5.155725000 5.147106000 2.066965000

C 6.153999000 6.654680000 3.937097000

H 6.877164000 7.469233000 4.017062000

H 6.490106000 5.847357000 4.600947000

H 5.205444000 7.020383000 4.351357000

C 5.155905000 5.146968000 0.642368000

C 10.230706000 5.296125000 0.199872000

H 11.006271000 3.558003000 1.354489000

C 9.514330000 7.743595000 -0.240117000

H 10.445060000 8.320564000 -0.334144000

H 8.756939000 8.432764000 0.145170000

H 9.217834000 7.456952000 -1.253597000

C 10.531609000 4.930146000 -1.226685000

H 11.364526000 5.527597000 -1.620204000

H 9.683343000 5.099951000 -1.902252000

H 10.818086000 3.879859000 -1.314115000

C 5.986108000 6.214748000 0.199796000

H 7.103136000 7.755620000 1.354599000

C 4.229063000 4.364957000 -0.240339000

H 3.265410000 4.883404000 -0.343000000

H 4.008159000 3.367793000 0.151876000

H 4.630281000 4.241952000 -1.250911000

C 6.154925000 6.654028000 -1.227796000

H 5.206515000 7.019557000 -1.642542000

H 6.491370000 5.846565000 -1.891307000

H 6.878063000 7.468619000 -1.307660000

C 4.228710000 4.365203000 2.949580000

H 4.007890000 3.367980000 2.557457000

H 3.265033000 4.883656000 3.051976000

H 4.629716000 4.242342000 3.960255000

C 9.514499000 7.743353000 2.949977000

H 8.756595000 8.432244000 2.565202000

H 10.445055000 8.320681000 3.043506000

H 9.218708000 7.456501000 3.963602000

**^Pu^Int3**

E=-2089.76655723 Ha

Sum of electronic and thermal Enthalpies= -2088.946486 Ha

Sum of electronic and thermal Free Energies= -2089.094983 Ha

Pu -1.520784000 -0.853620000 1.685160000

C 0.642808000 0.730319000 2.347424000

C -1.684883000 -3.347146000 0.637681000

C -0.645496000 0.063826000 4.145116000

S 2.391851000 1.548533000 -3.334719000

C -0.365438000 -3.327629000 2.506499000

C 0.428823000 -2.914164000 1.398276000

C 0.507968000 -0.234659000 3.376754000

C -4.154809000 -0.045631000 1.597096000

C 1.922716000 -2.781625000 1.388145000

H 2.271563000 -2.140647000 0.573089000

H 2.396536000 -3.762992000 1.244833000

H 2.320070000 -2.375503000 2.323409000

C -4.158597000 -1.097121000 0.638485000

C -3.308568000 -1.368292000 -1.808943000

H -4.172765000 -1.088781000 -2.427965000

H -3.321660000 -2.459530000 -1.728834000

H -2.413965000 -1.086942000 -2.372611000

C -3.378123000 1.011887000 1.059674000

H -3.219725000 1.974435000 1.526285000

C -0.394089000 -2.918298000 0.237553000

C -1.681567000 -3.589344000 2.034528000

C -1.222301000 1.244914000 3.600871000

C -2.797589000 -4.183878000 2.847339000

H -3.727545000 -4.220056000 2.275879000

H -2.998989000 -3.629825000 3.773408000

H -2.561552000 -5.213097000 3.148142000

C -4.968498000 -0.013809000 2.860835000

H -4.781816000 -0.874454000 3.516606000

H -6.043568000 -0.021647000 2.638997000

H -4.764204000 0.890066000 3.438874000

C -0.429424000 1.654646000 2.490676000

C 0.080931000 -2.686228000 -1.169838000

H -0.755818000 -2.654811000 -1.870952000

H 0.748269000 -3.494205000 -1.497571000

H 0.645846000 -1.752551000 -1.286157000

C 2.364294000 5.604915000 -2.958884000

H 1.639633000 6.411111000 -3.032103000

C 3.690493000 5.883530000 -2.623589000

H 3.999021000 6.908518000 -2.437530000

C 2.895351000 3.252033000 -3.098902000

C -3.373005000 -0.684245000 -0.475822000

C 4.225171000 3.531220000 -2.764087000

H 4.940803000 2.717753000 -2.694674000

C -5.064295000 -2.291659000 0.683292000

H -5.288301000 -2.597444000 1.709732000

H -4.650437000 -3.159444000 0.160695000

H -6.026839000 -2.065245000 0.203321000

C 4.617875000 4.847702000 -2.524137000

H 5.650252000 5.062550000 -2.262326000

C -2.880852000 0.623917000 -0.209932000

C -2.118614000 1.482547000 -1.179952000

H -2.731943000 1.724572000 -2.057718000

H -1.209271000 1.000993000 -1.561623000

H -1.822738000 2.428560000 -0.721240000

C 1.788384000 0.869693000 1.384237000

H 2.464643000 0.014420000 1.446881000

H 2.379577000 1.768431000 1.603349000

H 1.465459000 0.959975000 0.338983000

C 1.962405000 4.292164000 -3.190635000

H 0.931603000 4.064751000 -3.444774000

C -2.293741000 2.057249000 4.264935000

H -2.886908000 2.641301000 3.554691000

H -1.851861000 2.775260000 4.970105000

H -2.984045000 1.433509000 4.840870000

C -0.522184000 2.970455000 1.776943000

H -0.092163000 2.922300000 0.771993000

H 0.033655000 3.746161000 2.322373000

H -1.550997000 3.330676000 1.681613000

H 1.195927000 -1.046182000 3.569165000

C -1.082469000 -0.613767000 5.413859000

H -2.153521000 -0.853746000 5.427833000

H -0.533601000 -1.543996000 5.576870000

H -0.898700000 0.027707000 6.285846000

H -2.526943000 -3.500873000 -0.022922000

C 0.146648000 -3.711202000 3.862895000

H -0.637646000 -3.666195000 4.624434000

H 0.521216000 -4.744574000 3.855801000

H 0.974743000 -3.081072000 4.201065000

S 2.255614000 1.348213000 -5.428640000

C 5.772160000 -0.488988000 -6.343697000

H 6.178660000 -1.496210000 -6.317426000

C 6.548614000 0.567176000 -6.822937000

H 7.561816000 0.382026000 -7.168740000

C 3.946060000 1.040043000 -5.936942000

C 4.723845000 2.098958000 -6.419853000

H 4.305815000 3.100496000 -6.445960000

C 6.024051000 1.858362000 -6.862105000

H 6.626351000 2.681101000 -7.237323000

C 4.471253000 -0.257397000 -5.904163000

H 3.857814000 -1.073066000 -5.533718000

**^Pu^TS2**

E=-2089.71837766 Ha

Sum of electronic and thermal Enthalpies= -2088.898643 Ha

Sum of electronic and thermal Free Energies= -2089.033179 Ha

Pu 7.942725000 2.373366000 18.456349000

C 10.156315000 3.614219000 19.583287000

C 9.357526000 0.316946000 17.291459000

C 8.424202000 3.222652000 21.072436000

S 9.256396000 3.201560000 16.088383000

C 7.613167000 -0.477929000 18.542165000

C 8.704007000 -0.221471000 19.416018000

C 9.630714000 2.705328000 20.539086000

C 5.201132000 2.223007000 19.170082000

C 8.772669000 -0.701512000 20.835197000

H 9.535265000 -0.180039000 21.420359000

H 9.026028000 -1.770806000 20.869708000

H 7.817287000 -0.588349000 21.357000000

C 5.125634000 1.793107000 17.812866000

C 5.459503000 2.888578000 15.477660000

H 5.098234000 3.861829000 15.124813000

H 4.753632000 2.134202000 15.116710000

H 6.418365000 2.703756000 14.980648000

C 5.611444000 3.575330000 19.149004000

H 5.624897000 4.223252000 20.010669000

C 9.806670000 0.232551000 18.627716000

C 8.015368000 -0.131333000 17.213907000

C 8.213700000 4.490841000 20.462309000

C 7.308690000 -0.426891000 15.921642000

H 6.731453000 0.417019000 15.532770000

H 6.626169000 -1.276311000 16.027587000

H 8.040750000 -0.681993000 15.150778000

C 4.690007000 1.507119000 20.390791000

H 5.446904000 0.903099000 20.904483000

H 3.858668000 0.838962000 20.144437000

H 4.312388000 2.229363000 21.121818000

C 9.251964000 4.712714000 19.515588000

C 11.251183000 0.224880000 19.027582000

H 11.859543000 0.817749000 18.340962000

H 11.630252000 -0.805263000 18.988298000

H 11.437262000 0.585056000 20.043085000

C 8.515452000 6.664498000 14.059842000

H 7.687904000 7.118663000 13.521008000

C 9.742080000 7.324663000 14.143066000

H 9.872603000 8.296354000 13.674990000

C 9.396929000 4.818311000 15.359645000

C 5.549129000 2.869445000 16.976716000

C 10.638278000 5.470859000 15.409769000

H 11.470691000 4.986878000 15.910537000

C 4.343687000 0.612264000 17.318560000

H 4.078568000 -0.070362000 18.127012000

H 4.852743000 0.034641000 16.545805000

H 3.402069000 0.968102000 16.878425000

C 10.803054000 6.720832000 14.815983000

H 11.769122000 7.215648000 14.869342000

C 5.832350000 3.987612000 17.808670000

C 6.075258000 5.395842000 17.349065000

H 5.348089000 5.675124000 16.576085000

H 7.067066000 5.561909000 16.918069000

H 5.946211000 6.099602000 18.175617000

C 11.520419000 3.572848000 18.951356000

H 12.203327000 2.962687000 19.547939000

H 11.942980000 4.582494000 18.906566000

H 11.517443000 3.178353000 17.930496000

C 8.340614000 5.419822000 14.660146000

H 7.393108000 4.900341000 14.575054000

C 7.308175000 5.567115000 20.980293000

H 6.851678000 6.166270000 20.187431000

H 7.888206000 6.260641000 21.604341000

H 6.508617000 5.171760000 21.613221000

C 9.506074000 6.016479000 18.822166000

H 10.053093000 5.889865000 17.886935000

H 10.099466000 6.682879000 19.464668000

H 8.578037000 6.544377000 18.588222000

H 10.118074000 1.807781000 20.887766000

C 7.680767000 2.678022000 22.256595000

H 6.603649000 2.859126000 22.195286000

H 7.831210000 1.600552000 22.362591000

H 8.031551000 3.144630000 23.187054000

H 9.967279000 0.575508000 16.434145000

C 6.432352000 -1.315672000 18.935729000

H 5.808009000 -1.558569000 18.075322000

H 6.781320000 -2.266067000 19.360216000

H 5.791024000 -0.850781000 19.689399000

S 9.243165000 1.868228000 13.804080000

C 13.213144000 1.949771000 13.004273000

H 14.092800000 1.368767000 13.269144000

C 13.330799000 3.043402000 12.142951000

H 14.301517000 3.315567000 11.737492000

C 10.818191000 2.332148000 13.183712000

C 10.952242000 3.438001000 12.321761000

H 10.067763000 4.013361000 12.067393000

C 12.197146000 3.783544000 11.803723000

H 12.283398000 4.635706000 11.134720000

C 11.971233000 1.595087000 13.519915000

H 11.872038000 0.740484000 14.182441000

**^Pu^Int4**

E=-2089.71953880 Ha

Sum of electronic and thermal Enthalpies= -2088.898907 Ha

Sum of electronic and thermal Free Energies= -2089.036410 Ha

Pu 7.938413000 2.387221000 18.486221000

C 10.133732000 3.603172000 19.643211000

C 9.346838000 0.319436000 17.342216000

C 8.393955000 3.208340000 21.124916000

S 9.199971000 3.144505000 16.107458000

C 7.582249000 -0.466837000 18.573213000

C 8.660523000 -0.217824000 19.460210000

C 9.601192000 2.691558000 20.597756000

C 5.196770000 2.246840000 19.195939000

C 8.708268000 -0.700612000 20.879036000

H 9.475143000 -0.193899000 21.471226000

H 8.942096000 -1.774306000 20.913033000

H 7.750620000 -0.571633000 21.392761000

C 5.123026000 1.813052000 17.839610000

C 5.463991000 2.898037000 15.500663000

H 5.163225000 3.887764000 15.138907000

H 4.714485000 2.183566000 15.145855000

H 6.413799000 2.652706000 15.012998000

C 5.608879000 3.597625000 19.170982000

H 5.615759000 4.250537000 20.028741000

C 9.777477000 0.231045000 18.685888000

C 8.005759000 -0.120081000 17.247206000

C 8.190482000 4.479718000 20.516767000

C 7.315145000 -0.409190000 15.945362000

H 6.721310000 0.427792000 15.567592000

H 6.652301000 -1.276446000 16.031223000

H 8.059888000 -0.632976000 15.176894000

C 4.681851000 1.532832000 20.415601000

H 5.431932000 0.911046000 20.917718000

H 3.836222000 0.882224000 20.170675000

H 4.323488000 2.256536000 21.154584000

C 9.233766000 4.699702000 19.571532000

C 11.217352000 0.203727000 19.099838000

H 11.838280000 0.796171000 18.424419000

H 11.583779000 -0.830614000 19.051990000

H 11.397625000 0.549748000 20.121227000

C 8.544144000 6.582858000 14.008723000

H 7.735277000 7.025775000 13.432953000

C 9.767345000 7.244062000 14.118828000

H 9.916135000 8.205944000 13.636134000

C 9.375686000 4.756810000 15.376610000

C 5.549918000 2.887563000 16.999397000

C 10.614129000 5.414392000 15.455338000

H 11.429010000 4.942173000 15.995007000

C 4.335414000 0.637567000 17.343724000

H 4.063181000 -0.044186000 18.150330000

H 4.843068000 0.059284000 16.570179000

H 3.397989000 1.001183000 16.900946000

C 10.802388000 6.650220000 14.840045000

H 11.767581000 7.144178000 14.917104000

C 5.832927000 4.005633000 17.826711000

C 6.090647000 5.408844000 17.361968000

H 5.342836000 5.705448000 16.615650000

H 7.069748000 5.547670000 16.893975000

H 6.008745000 6.114405000 18.192720000

C 11.492697000 3.554723000 19.002950000

H 12.171553000 2.923316000 19.581304000

H 11.928205000 4.559379000 18.973294000

H 11.469007000 3.181303000 17.974348000

C 8.348178000 5.349303000 14.626706000

H 7.403161000 4.829456000 14.518231000

C 7.291337000 5.558577000 21.039215000

H 6.848423000 6.171833000 20.249485000

H 7.874550000 6.238435000 21.675159000

H 6.483041000 5.164379000 21.661616000

C 9.485000000 5.992717000 18.857734000

H 9.991144000 5.845631000 17.902452000

H 10.116842000 6.652486000 19.469342000

H 8.558610000 6.535885000 18.655407000

H 10.088029000 1.794722000 20.949035000

C 7.645393000 2.668589000 22.308227000

H 6.566723000 2.834696000 22.234469000

H 7.808610000 1.594730000 22.429131000

H 7.981133000 3.151374000 23.235869000

H 9.969277000 0.578437000 16.494089000

C 6.396878000 -1.306921000 18.946509000

H 5.777568000 -1.536217000 18.079140000

H 6.742033000 -2.264010000 19.359078000

H 5.752985000 -0.851888000 19.704062000

S 9.431427000 1.611433000 13.589002000

C 13.367851000 2.073497000 12.780318000

H 14.296534000 1.555557000 13.005798000

C 13.384751000 3.227243000 11.991739000

H 14.325813000 3.607777000 11.603782000

C 10.947771000 2.236648000 12.989714000

C 10.982965000 3.403896000 12.197165000

H 10.050327000 3.916094000 11.983235000

C 12.189244000 3.889192000 11.703956000

H 12.198506000 4.788340000 11.093619000

C 12.165845000 1.582852000 13.276648000

H 12.144136000 0.686231000 13.888767000

**(Cp^Me4^)_2_**

E=-701.323814944 Ha

Sum of electronic and thermal Enthalpies= -700.911561 Ha

Sum of electronic and thermal Free Energies= -700.982722 Ha

C 8.950634000 8.009472000 17.398345000

C 5.717552000 7.465746000 17.998858000

C 8.154971000 5.782777000 17.124389000

C 5.530691000 7.205896000 15.639800000

C 9.189625000 5.789291000 18.000182000

C 4.394585000 6.766050000 16.231410000

C 7.925955000 7.196674000 16.638577000

C 4.512694000 6.922849000 17.697048000

C 9.687423000 7.171748000 18.166175000

C 3.166696000 6.212903000 15.578171000

C 6.465622000 7.724520000 16.709966000

C 9.095533000 9.484838000 17.218756000

C 3.413050000 6.535926000 18.636598000

C 9.804140000 4.624161000 18.711937000

H 6.526728000 8.818574000 16.570750000

H 8.217030000 7.239462000 15.573902000

C 5.864765000 7.269912000 14.185867000

H 6.109325000 8.292969000 13.870623000

H 6.737657000 6.650793000 13.936097000

H 5.036002000 6.925356000 13.561769000

C 6.262141000 7.832452000 19.339811000

H 6.506216000 8.901250000 19.396225000

H 5.548181000 7.616589000 20.139543000

H 7.188438000 7.288297000 19.555349000

C 7.365018000 4.616966000 16.628186000

H 6.301731000 4.724894000 16.869685000

H 7.430476000 4.522008000 15.536454000

H 7.716890000 3.675938000 17.060233000

C 10.843199000 7.509627000 19.055155000

H 10.641841000 7.228907000 20.096662000

H 11.750474000 6.970280000 18.755581000

H 11.074014000 8.577676000 19.041613000

H 9.948519000 9.881279000 17.775997000

H 8.204711000 10.031372000 17.558423000

H 9.239994000 9.752220000 16.163576000

H 9.765601000 4.753122000 19.800841000

H 10.862648000 4.505175000 18.447681000

H 9.297977000 3.685470000 18.474311000

H 2.279338000 6.809689000 15.823676000

H 3.255521000 6.186719000 14.489290000

H 2.959495000 5.190099000 15.917334000

H 2.491848000 7.094505000 18.427615000

H 3.680760000 6.721229000 19.679601000

H 3.161945000 5.471769000 18.544771000

**2-Pu**

E=-2994.69115319 Ha

Sum of electronic and thermal Enthalpies= -2993.661831 Ha

Sum of electronic and thermal Free Energies= -2993.833989 Ha

Pu 5.083284000 5.159065000 11.534059000

S 3.858244000 4.501893000 8.976702000

C 2.541042000 3.315634000 8.761704000

C 1.411024000 3.637219000 7.997675000

H 1.336755000 4.622508000 7.551897000

C 0.385144000 2.709838000 7.824464000

H -0.482493000 2.979465000 7.227612000

C 0.463337000 1.449661000 8.416001000

H -0.338424000 0.729243000 8.282415000

C 2.615333000 2.046216000 9.351242000

H 3.490604000 1.779824000 9.932604000

C 1.582813000 1.125337000 9.181086000

H 1.660059000 0.146067000 9.646687000

Pu 5.045522000 6.003672000 6.797457000

S 6.270808000 6.660584000 9.354739000

C 7.587735000 7.847163000 9.569579000

C 8.718670000 7.525454000 10.332179000

H 8.793918000 6.539812000 10.777017000

C 9.744281000 8.453198000 10.505111000

H 10.612672000 8.183476000 11.100823000

C 9.664910000 9.713824000 9.914698000

H 10.466468000 10.434504000 10.048100000

C 7.512281000 9.117018000 8.981137000

H 6.636236000 9.383461000 8.400968000

C 8.544521000 10.038264000 9.150981000

H 8.466339000 11.017891000 8.686289000

C 2.619461000 6.027913000 12.286573000

C 3.255812000 5.736599000 13.525642000

C 2.744395000 4.771155000 14.554243000

H 2.262023000 3.901536000 14.097768000

H 1.993577000 5.250513000 15.197370000

H 3.537302000 4.404081000 15.212282000

C 3.291482000 7.139866000 11.717442000

H 3.053783000 7.597986000 10.763604000

C 4.332826000 7.550958000 12.587884000

C 4.315437000 6.678556000 13.712054000

C 5.209733000 8.757556000 12.413498000

H 6.252771000 8.571076000 12.692912000

H 4.858968000 9.590270000 13.037304000

H 5.209371000 9.098612000 11.375266000

C 1.391314000 5.358789000 11.740033000

H 1.267550000 5.571609000 10.675180000

H 0.489380000 5.710426000 12.258220000

H 1.415636000 4.268816000 11.851443000

C 5.107265000 6.879154000 14.970516000

H 5.295912000 5.941863000 15.502046000

H 4.569406000 7.536982000 15.666992000

H 6.074718000 7.350418000 14.772815000

C 5.495207000 2.713429000 12.674155000

C 6.323566000 2.678089000 11.518449000

C 7.385176000 3.614322000 11.705218000

C 7.215681000 4.230025000 12.976207000

C 6.047137000 3.675615000 13.558758000

H 5.672371000 3.902788000 14.549134000

C 4.322160000 1.819632000 12.966790000

H 3.974996000 1.965074000 13.992884000

H 4.598235000 0.762942000 12.863997000

H 3.461709000 1.984523000 12.305666000

C 6.234053000 1.708863000 10.374663000

H 5.550239000 0.885910000 10.605497000

H 7.214322000 1.262290000 10.168858000

H 5.887745000 2.174362000 9.444895000

C 8.162148000 5.190100000 13.641258000

H 8.302939000 6.125056000 13.084716000

H 9.156560000 4.742691000 13.762367000

H 7.804016000 5.455827000 14.638966000

C 8.577831000 3.740321000 10.801650000

H 8.323002000 4.100223000 9.799337000

H 9.073339000 2.768178000 10.679246000

H 9.322293000 4.423939000 11.219480000

C 7.509311000 5.134556000 6.045315000

C 6.873366000 5.426709000 4.806227000

C 7.385611000 6.392734000 3.778572000

H 7.864892000 7.263645000 4.235871000

H 8.139288000 5.914621000 3.137855000

H 6.593837000 6.757706000 3.118005000

C 6.836864000 4.022508000 6.613685000

H 7.074289000 3.563750000 7.567280000

C 5.795673000 3.612115000 5.742736000

C 5.813615000 4.485073000 4.618988000

C 4.918457000 2.405625000 5.916326000

H 3.875504000 2.592507000 5.636851000

H 5.269101000 1.573140000 5.292148000

H 4.918603000 2.064051000 6.954392000

C 8.737380000 5.803296000 6.592478000

H 8.863517000 5.585752000 7.656095000

H 9.638901000 5.455584000 6.070942000

H 8.711068000 6.893754000 6.486239000

C 5.022222000 4.285034000 3.360166000

H 4.834701000 5.222384000 2.828343000

H 5.559863000 3.626601000 2.664097000

H 4.054249000 3.814646000 3.557405000

C 4.633235000 8.449747000 5.658106000

C 3.804503000 8.484487000 6.813559000

C 2.743119000 7.548115000 6.626095000

C 2.913225000 6.932833000 5.355001000

C 4.081867000 7.487691000 4.773033000

H 4.457044000 7.260815000 3.782752000

C 5.806122000 9.343975000 5.366178000

H 6.154242000 9.198373000 4.340429000

H 5.529465000 10.400576000 5.468378000

H 6.666035000 9.179688000 6.028130000

C 3.893340000 9.453289000 7.957746000

H 4.576584000 10.276889000 7.727390000

H 2.912735000 9.899105000 8.163603000

H 4.239798000 8.987633000 8.887377000

C 1.967214000 5.972785000 4.689256000

H 1.827225000 5.037095000 5.244783000

H 0.972450000 6.419625000 4.568956000

H 2.325212000 5.708424000 3.691137000

C 1.550113000 7.421638000 7.529151000

H 1.804391000 7.060618000 8.531208000

H 1.054936000 8.393845000 7.652375000

H 0.805529000 6.738745000 7.110353000

**1-U**

E=-1529.16443917 Ha

Sum of electronic and thermal Enthalpies= -1528.544293 Ha

Sum of electronic and thermal Free Energies= -1528.646525 Ha

U 7.822742000 4.540980000 1.354791000

C 6.490684000 2.135348000 1.354676000

C 7.308778000 2.035827000 2.509098000

C 8.648860000 1.855894000 2.067172000

C 8.648746000 1.856071000 0.641749000

C 7.308600000 2.036111000 0.200085000

C 6.840390000 1.960583000 3.935763000

C 9.793167000 1.446743000 2.946172000

C 9.792874000 1.447197000 -0.237614000

C 6.839998000 1.960913000 -1.226509000

H 5.413293000 2.231189000 1.354718000

H 5.771581000 2.172096000 4.011469000

H 7.363968000 2.658675000 4.601813000

H 7.002650000 0.957250000 4.350531000

H 10.762138000 1.792408000 2.573463000

H 9.855872000 0.351464000 3.014300000

H 9.677973000 1.819319000 3.968525000

H 9.854596000 0.351956000 -0.307346000

H 10.762060000 1.791381000 0.135899000

H 9.678326000 1.821385000 -1.259453000

H 7.003090000 0.957853000 -1.641599000

H 7.362809000 2.659672000 -1.892474000

H 5.770992000 2.171519000 -1.301927000

C 10.565141000 4.540437000 1.354945000

C 10.239993000 5.295565000 2.510364000

C 6.436995000 6.926201000 1.354425000

C 5.961408000 6.258223000 2.508766000

C 9.728435000 6.550542000 2.066789000

C 10.554718000 4.935520000 3.935866000

H 10.805863000 3.876741000 4.032051000

H 9.726147000 5.144241000 4.624210000

H 11.416160000 5.507572000 4.305865000

C 9.728547000 6.550605000 0.643170000

C 5.156776000 5.167473000 2.068025000

C 6.121014000 6.704150000 3.935228000

H 6.850331000 7.513210000 4.017714000

H 6.443658000 5.896952000 4.605646000

H 5.171630000 7.079658000 4.339162000

C 5.156801000 5.167233000 0.641348000

C 10.240203000 5.295707000 0.199555000

H 11.021700000 3.559787000 1.354878000

C 9.513275000 7.743253000 -0.240560000

H 10.445731000 8.314125000 -0.352949000

H 8.766493000 8.436862000 0.156632000

H 9.196909000 7.455600000 -1.247776000

C 10.555166000 4.935815000 -1.225928000

H 11.415135000 5.509699000 -1.596501000

H 9.725894000 5.142264000 -1.914147000

H 10.808720000 3.877574000 -1.321648000

C 5.961463000 6.257815000 0.200284000

H 7.052397000 7.816375000 1.354215000

C 4.241033000 4.371788000 -0.241850000

H 3.273101000 4.880424000 -0.354359000

H 4.027618000 3.375722000 0.156794000

H 4.648855000 4.244445000 -1.249470000

C 6.121274000 6.703281000 -1.226300000

H 5.171868000 7.078288000 -1.630639000

H 6.444444000 5.895997000 -1.896366000

H 6.850302000 7.512607000 -1.308827000

C 4.241064000 4.372290000 2.951514000

H 4.027185000 3.376324000 2.552883000

H 3.273333000 4.881224000 3.064419000

H 4.649194000 4.244762000 3.958988000

C 9.513033000 7.743217000 2.950445000

H 8.765087000 8.435958000 2.553919000

H 10.445050000 8.315031000 3.061638000

H 9.198065000 7.455512000 3.958080000

**^U^Int3**

E=-2012.73338354 Ha

Sum of electronic and thermal Enthalpies= -2011.913441 Ha

Sum of electronic and thermal Free Energies= -2012.061297 Ha

U -1.121761000 -0.706196000 1.722939000

C 1.275096000 0.685424000 1.956374000

C -0.678030000 -2.845330000 0.027403000

C 0.412296000 -0.067958000 3.962897000

S 1.503783000 1.124537000 -3.819550000

C -1.358817000 -3.536033000 2.098820000

C 0.054981000 -3.335882000 2.139911000

C 1.311665000 -0.367413000 2.907182000

C -3.601993000 0.469345000 2.285077000

C 0.944892000 -3.794393000 3.256549000

H 1.892174000 -3.249353000 3.299726000

H 1.199355000 -4.856030000 3.129539000

H 0.463556000 -3.703105000 4.234442000

C -4.009631000 -0.490757000 1.311369000

C -3.710749000 -0.714914000 -1.288603000

H -2.840979000 -1.201107000 -1.746917000

H -4.031590000 0.072468000 -1.982463000

H -4.515590000 -1.453236000 -1.251831000

C -2.800821000 1.431642000 1.623332000

H -2.398996000 2.328966000 2.072044000

C 0.476225000 -2.905672000 0.855781000

C -1.803944000 -3.290521000 0.770635000

C -0.162496000 1.204665000 3.680597000

C -3.083136000 -3.802261000 0.180628000

H -3.916419000 -3.775093000 0.884570000

H -2.952031000 -4.852764000 -0.114187000

H -3.375653000 -3.255204000 -0.716590000

C -4.145162000 0.545729000 3.684109000

H -3.995908000 -0.378905000 4.256610000

H -5.228516000 0.728273000 3.673487000

H -3.686254000 1.361181000 4.246560000

C 0.371767000 1.669742000 2.449327000

C 1.889453000 -2.774632000 0.368547000

H 1.962510000 -2.086617000 -0.479921000

H 2.269873000 -3.746085000 0.024687000

H 2.577619000 -2.423684000 1.143700000

C 0.055077000 4.337376000 -5.857501000

H -0.753605000 4.544189000 -6.552980000

C 0.898825000 5.367041000 -5.438301000

H 0.747670000 6.377038000 -5.809064000

C 1.284057000 2.773694000 -4.485262000

C -3.426507000 -0.132193000 0.066360000

C 2.129289000 3.806609000 -4.063253000

H 2.932555000 3.588675000 -3.366263000

C -5.097650000 -1.493513000 1.562248000

H -4.862217000 -2.188795000 2.376483000

H -5.323495000 -2.088863000 0.675685000

H -6.024788000 -0.979968000 1.851106000

C 1.932912000 5.101510000 -4.541588000

H 2.589156000 5.902533000 -4.212602000

C -2.658096000 1.053684000 0.263726000

C -2.000847000 1.848370000 -0.830951000

H -2.743402000 2.195240000 -1.562193000

H -1.255458000 1.279522000 -1.402718000

H -1.504903000 2.735870000 -0.431488000

C 2.172792000 0.849071000 0.760375000

H 2.761131000 -0.053450000 0.580373000

H 2.882279000 1.673123000 0.912231000

H 1.629708000 1.075840000 -0.165921000

C 0.241856000 3.042387000 -5.380783000

H -0.411681000 2.235313000 -5.697731000

C -0.972765000 2.011239000 4.650761000

H -1.661368000 2.701783000 4.154074000

H -0.319131000 2.622387000 5.289210000

H -1.562966000 1.376719000 5.318731000

C 0.250879000 3.060275000 1.900099000

H 1.028024000 3.708903000 2.328483000

H -0.709282000 3.530320000 2.132238000

H 0.382033000 3.088601000 0.814164000

H 1.962275000 -1.229176000 2.859277000

C 0.235879000 -0.828399000 5.248070000

H -0.810276000 -1.085546000 5.461197000

H 0.808217000 -1.758252000 5.238129000

H 0.589714000 -0.239714000 6.104483000

H -0.670780000 -2.640298000 -1.039870000

C -2.179400000 -4.136546000 3.205910000

H -3.245366000 -3.921222000 3.084755000

H -2.076505000 -5.230202000 3.237079000

H -1.881096000 -3.765951000 4.193163000

S 2.623203000 0.128143000 -5.301988000

C 6.370768000 0.243670000 -3.714445000

H 6.932875000 -0.289434000 -2.952702000

C 6.979790000 1.260956000 -4.450877000

H 8.017335000 1.521469000 -4.261045000

C 4.315855000 0.589830000 -4.938027000

C 4.927253000 1.608049000 -5.678558000

H 4.357192000 2.131007000 -6.440241000

C 6.259227000 1.939838000 -5.432498000

H 6.732779000 2.729714000 -6.008995000

C 5.042852000 -0.097104000 -3.958239000

H 4.562002000 -0.890121000 -3.393707000

**^U^TS2**

E= -2012.72101897 Ha

Sum of electronic and thermal Enthalpies= -2011.901389 Ha

Sum of electronic and thermal Free Energies= -2012.036455 Ha

U 8.131841000 2.265437000 17.902784000

C 10.520820000 3.539720000 18.502031000

C 9.094826000 0.061502000 16.545815000

C 9.365018000 2.891419000 20.395586000

S 8.915754000 2.908946000 15.041911000

C 7.489115000 -0.521150000 18.064214000

C 8.745301000 -0.436629000 18.743924000

C 10.357615000 2.504631000 19.457041000

C 5.616271000 2.844697000 18.947953000

C 8.970859000 -0.929871000 20.142421000

H 9.932421000 -0.601177000 20.545390000

H 8.972553000 -2.028857000 20.167793000

H 8.191061000 -0.600603000 20.836039000

C 5.161793000 2.197341000 17.760106000

C 5.041245000 2.797709000 15.245411000

H 5.665478000 3.332954000 14.526563000

H 4.014999000 3.172980000 15.123819000

H 5.033375000 1.743216000 14.958437000

C 6.190857000 4.080491000 18.552784000

H 6.526990000 4.856641000 19.221625000

C 9.745351000 -0.120300000 17.788969000

C 7.707858000 -0.217752000 16.693920000

C 8.941321000 4.200462000 20.043598000

C 6.772318000 -0.412107000 15.537850000

H 5.723088000 -0.387854000 15.837359000

H 6.949527000 -1.385988000 15.062549000

H 6.920927000 0.353809000 14.770443000

C 5.293185000 2.417920000 20.353065000

H 5.603612000 1.389650000 20.575432000

H 4.212526000 2.466435000 20.541506000

H 5.773781000 3.071154000 21.085419000

C 9.627131000 4.591765000 18.864628000

C 11.230870000 -0.248902000 17.960601000

H 11.785790000 0.573337000 17.499291000

H 11.578097000 -1.177235000 17.489138000

H 11.530198000 -0.302996000 19.011146000

C 7.701557000 5.611704000 12.265301000

H 6.985983000 5.639677000 11.448185000

C 8.484342000 6.732264000 12.550479000

H 8.376831000 7.636776000 11.958577000

C 8.748634000 4.407072000 14.084991000

C 5.517222000 3.004752000 16.651654000

C 9.551970000 5.521064000 14.349808000

H 10.290707000 5.471376000 15.140883000

C 4.125371000 1.113938000 17.724783000

H 4.114480000 0.516166000 18.637659000

H 4.230845000 0.433781000 16.875786000

H 3.129813000 1.571492000 17.632570000

C 9.411478000 6.681715000 13.589617000

H 10.035367000 7.544137000 13.807926000

C 6.147794000 4.187951000 17.144505000

C 6.367607000 5.454214000 16.369272000

H 5.450730000 6.059958000 16.376652000

H 6.620956000 5.271076000 15.324134000

H 7.160966000 6.069868000 16.799186000

C 11.616704000 3.632185000 17.480096000

H 11.799146000 2.684995000 16.966722000

H 12.561030000 3.935007000 17.952259000

H 11.391767000 4.369749000 16.706720000

C 7.832017000 4.452682000 13.024687000

H 7.235091000 3.575455000 12.796630000

C 8.152593000 5.095130000 20.952669000

H 7.776442000 5.989193000 20.447206000

H 8.791419000 5.446537000 21.775024000

H 7.299125000 4.588913000 21.414000000

C 9.596480000 5.962725000 18.257538000

H 10.448044000 6.565949000 18.601243000

H 8.689142000 6.508355000 18.531438000

H 9.639931000 5.939462000 17.165741000

H 10.962001000 1.613321000 19.527772000

C 9.026522000 2.203008000 21.688143000

H 7.958301000 1.982379000 21.799092000

H 9.569995000 1.262191000 21.790433000

H 9.305379000 2.833143000 22.542864000

H 9.594761000 0.233126000 15.599307000

C 6.280630000 -1.165319000 18.677098000

H 5.456436000 -1.239858000 17.967615000

H 6.525353000 -2.187062000 18.997021000

H 5.910002000 -0.640118000 19.564382000

S 10.128807000 1.723685000 13.511673000

C 14.065951000 2.145472000 14.398044000

H 14.811951000 1.617401000 14.985771000

C 14.422979000 3.284197000 13.674139000

H 15.447376000 3.645279000 13.698847000

C 11.783214000 2.351174000 13.607513000

C 12.151064000 3.490720000 12.872917000

H 11.403083000 3.998377000 12.272329000

C 13.464182000 3.953330000 12.912211000

H 13.741248000 4.834554000 12.339844000

C 12.754066000 1.680684000 14.369533000

H 12.470360000 0.792639000 14.925919000

**^U^Int4**

E=-2012.73631638 Ha

Sum of electronic and thermal Enthalpies= -2011.915193 Ha

Sum of electronic and thermal Free Energies= -2012.053225 Ha

U -0.576179000 -0.296833000 1.036247000

C 1.845458000 0.813016000 1.780355000

C 0.671663000 -2.432303000 -0.227701000

C 0.340902000 0.598606000 3.530248000

S 0.291550000 0.269616000 -1.567528000

C -1.088742000 -3.058070000 1.095668000

C 0.067705000 -2.928663000 1.920098000

C 1.403488000 -0.030545000 2.836450000

C -3.215628000 -0.182247000 2.082365000

C 0.147867000 -3.383504000 3.347082000

H 0.979542000 -2.920374000 3.886186000

H 0.301770000 -4.470400000 3.400570000

H -0.770814000 -3.169083000 3.902292000

C -3.523547000 -0.611324000 0.759975000

C -3.454980000 0.425583000 -1.605848000

H -3.165895000 1.372297000 -2.065108000

H -4.537259000 0.306937000 -1.749527000

H -2.959643000 -0.376098000 -2.161921000

C -2.672128000 1.120093000 1.978548000

H -2.454486000 1.755063000 2.821955000

C 1.170578000 -2.591730000 1.083549000

C -0.715011000 -2.731949000 -0.242899000

C 0.141147000 1.867028000 2.921193000

C -1.512250000 -2.930749000 -1.498384000

H -2.536123000 -2.560520000 -1.421109000

H -1.568827000 -3.997597000 -1.751290000

H -1.035590000 -2.414204000 -2.334792000

C -3.587140000 -0.861164000 3.371981000

H -2.821066000 -1.547922000 3.751832000

H -4.509260000 -1.441279000 3.264439000

H -3.764003000 -0.118395000 4.156264000

C 1.039893000 1.986670000 1.825752000

C 2.624858000 -2.742021000 1.415786000

H 3.255107000 -2.174135000 0.728739000

H 2.904071000 -3.798714000 1.311180000

H 2.885591000 -2.453925000 2.437873000

C -0.706910000 3.205110000 -4.202204000

H -1.491556000 3.372863000 -4.935762000

C 0.318867000 4.138324000 -4.048673000

H 0.334600000 5.041295000 -4.652640000

C 0.269760000 1.804207000 -2.471714000

C -3.121055000 0.411358000 -0.144897000

C 1.318710000 2.729157000 -2.353663000

H 2.129971000 2.524524000 -1.664026000

C -4.495036000 -1.693673000 0.393779000

H -4.676386000 -2.389255000 1.213777000

H -4.202647000 -2.276190000 -0.483050000

H -5.459188000 -1.228240000 0.145851000

C 1.335025000 3.888445000 -3.127911000

H 2.155464000 4.592907000 -3.016453000

C -2.600654000 1.497560000 0.609820000

C -2.283121000 2.862431000 0.070808000

H -3.208293000 3.427674000 -0.103402000

H -1.743461000 2.821864000 -0.878935000

H -1.679184000 3.444194000 0.769921000

C 3.060420000 0.616870000 0.917963000

H 2.830800000 0.184375000 -0.060911000

H 3.789009000 -0.025068000 1.419617000

H 3.551487000 1.578405000 0.731861000

C -0.729283000 2.046932000 -3.428821000

H -1.508474000 1.307829000 -3.577561000

C -0.612862000 3.008310000 3.537348000

H -1.181427000 3.604242000 2.817988000

H 0.095978000 3.691091000 4.025083000

H -1.304122000 2.671084000 4.315155000

C 1.252265000 3.244297000 1.036309000

H 2.310384000 3.373084000 0.785611000

H 0.949565000 4.121150000 1.617620000

H 0.700114000 3.269227000 0.093029000

H 1.867294000 -0.956646000 3.139149000

C -0.276091000 0.126445000 4.815136000

H -1.347158000 0.344384000 4.873373000

H -0.150266000 -0.951579000 4.946591000

H 0.197451000 0.613015000 5.678055000

H 1.266906000 -2.214858000 -1.107511000

C -2.321489000 -3.799545000 1.521482000

H -3.037492000 -3.883438000 0.704264000

H -2.047060000 -4.821633000 1.814660000

H -2.835321000 -3.353474000 2.377446000

S 1.651838000 -1.334375000 -3.775935000

C 5.520684000 -0.193192000 -3.495356000

H 6.417194000 -0.524304000 -2.977573000

C 5.574336000 0.913200000 -4.347935000

H 6.511722000 1.443350000 -4.493197000

C 3.149515000 -0.466092000 -3.979410000

C 3.220335000 0.656662000 -4.833206000

H 2.318105000 0.978930000 -5.342940000

C 4.420822000 1.334481000 -5.013600000

H 4.458832000 2.195201000 -5.676018000

C 4.324197000 -0.874880000 -3.309454000

H 4.274408000 -1.737964000 -2.652401000

**3-U**

E= -1770.99264185 Ha

Sum of electronic and thermal Enthalpies= -1770.271344 Ha

Sum of electronic and thermal Free Energies= -1770.387768 Ha

U 8.068152000 7.164625000 4.123255000

S 5.654667000 6.555301000 5.268276000

C 7.576221000 9.787145000 2.861397000

C 7.517385000 9.863022000 6.610480000

H 7.326998000 8.917381000 7.128663000

H 6.626312000 10.483976000 6.747880000

H 8.342702000 10.366564000 7.122344000

C 5.729696000 4.096030000 2.951852000

H 4.888809000 4.549889000 3.480114000

H 6.178258000 3.357431000 3.622085000

H 5.334181000 3.551293000 2.083202000

C 10.091304000 7.136493000 6.222428000

C 7.595172000 6.973197000 1.474396000

H 7.671833000 7.906296000 0.936890000

C 11.862026000 7.188382000 4.306284000

H 11.873085000 6.919781000 3.246072000

H 12.783213000 6.787601000 4.749626000

H 11.927582000 8.278566000 4.372574000

C 10.079804000 9.813406000 2.146128000

H 11.007971000 9.356409000 2.499504000

H 10.293433000 10.874809000 1.962292000

H 9.838183000 9.368741000 1.175601000

C 8.649173000 6.037891000 1.627330000

C 6.867860000 9.784497000 4.093971000

C 5.005636000 6.961128000 1.863928000

H 4.408039000 6.813961000 2.767799000

H 4.493345000 6.451580000 1.037051000

H 5.004123000 8.028242000 1.633773000

C 8.416030000 6.259959000 7.991107000

H 8.175136000 7.275951000 8.315946000

H 9.078093000 5.830424000 8.756450000

H 7.486880000 5.688932000 7.997941000

C 4.407840000 5.306163000 8.926277000

H 4.247265000 4.345634000 9.409745000

C 9.089013000 6.227168000 6.653186000

C 6.399896000 6.425625000 2.017471000

C 8.748675000 3.534936000 2.305865000

H 8.439073000 2.942408000 1.434258000

H 8.467770000 2.961366000 3.191933000

H 9.840353000 3.591551000 2.270249000

C 4.808437000 7.749006000 7.656827000

H 4.939381000 8.704204000 7.161637000

C 5.120949000 6.566820000 6.969816000

C 4.120239000 6.488974000 9.606984000

H 3.737568000 6.460045000 10.623258000

C 8.270117000 3.873583000 5.936918000

H 7.193500000 4.018430000 5.805040000

H 8.428944000 3.490765000 6.951880000

H 8.600181000 3.094961000 5.245842000

C 8.965993000 9.660781000 3.140317000

C 8.096905000 4.883596000 2.249764000

C 10.651862000 6.644670000 5.008428000

C 4.318302000 7.708037000 8.961304000

H 4.085015000 8.639119000 9.472177000

C 10.014219000 5.409235000 4.727534000

H 10.283453000 4.741740000 3.922058000

C 7.819302000 9.700483000 5.148436000

C 6.720778000 5.127435000 2.507544000

C 7.001297000 10.198044000 1.538862000

H 6.040562000 9.727951000 1.315241000

H 7.679198000 9.985333000 0.707501000

H 6.831674000 11.282956000 1.533931000

C 9.047946000 5.142174000 5.734016000

C 5.402125000 10.062177000 4.251138000

H 4.867429000 9.936509000 3.306258000

H 5.237381000 11.097046000 4.581437000

H 4.931687000 9.392069000 4.973689000

C 4.897289000 5.342174000 7.622597000

H 5.107478000 4.416098000 7.095629000

C 10.019762000 6.141243000 1.023693000

H 10.346961000 7.180046000 0.927938000

H 10.035087000 5.704929000 0.016048000

H 10.775740000 5.607978000 1.609057000

C 10.667604000 8.222260000 7.080628000

H 9.903687000 8.831634000 7.569301000

H 11.331549000 8.889867000 6.524838000

H 11.273391000 7.772936000 7.879081000

C 9.106218000 9.624586000 4.548892000

H 10.047670000 9.640663000 5.077816000

**Optimized geometries for the reactivity of 1-Pu and 1-U with (PhHN)_2_.**

**1-U**

E=-1529.16443917 Ha

Sum of electronic and thermal Enthalpies= -1528.544293 Ha

Sum of electronic and thermal Free Energies= -1528.646525 Ha

U 7.822742000 4.540980000 1.354791000

C 6.490684000 2.135348000 1.354676000

C 7.308778000 2.035827000 2.509098000

C 8.648860000 1.855894000 2.067172000

C 8.648746000 1.856071000 0.641749000

C 7.308600000 2.036111000 0.200085000

C 6.840390000 1.960583000 3.935763000

C 9.793167000 1.446743000 2.946172000

C 9.792874000 1.447197000 -0.237614000

C 6.839998000 1.960913000 -1.226509000

H 5.413293000 2.231189000 1.354718000

H 5.771581000 2.172096000 4.011469000

H 7.363968000 2.658675000 4.601813000

H 7.002650000 0.957250000 4.350531000

H 10.762138000 1.792408000 2.573463000

H 9.855872000 0.351464000 3.014300000

H 9.677973000 1.819319000 3.968525000

H 9.854596000 0.351956000 -0.307346000

H 10.762060000 1.791381000 0.135899000

H 9.678326000 1.821385000 -1.259453000

H 7.003090000 0.957853000 -1.641599000

H 7.362809000 2.659672000 -1.892474000

H 5.770992000 2.171519000 -1.301927000

C 10.565141000 4.540437000 1.354945000

C 10.239993000 5.295565000 2.510364000

C 6.436995000 6.926201000 1.354425000

C 5.961408000 6.258223000 2.508766000

C 9.728435000 6.550542000 2.066789000

C 10.554718000 4.935520000 3.935866000

H 10.805863000 3.876741000 4.032051000

H 9.726147000 5.144241000 4.624210000

H 11.416160000 5.507572000 4.305865000

C 9.728547000 6.550605000 0.643170000

C 5.156776000 5.167473000 2.068025000

C 6.121014000 6.704150000 3.935228000

H 6.850331000 7.513210000 4.017714000

H 6.443658000 5.896952000 4.605646000

H 5.171630000 7.079658000 4.339162000

C 5.156801000 5.167233000 0.641348000

C 10.240203000 5.295707000 0.199555000

H 11.021700000 3.559787000 1.354878000

C 9.513275000 7.743253000 -0.240560000

H 10.445731000 8.314125000 -0.352949000

H 8.766493000 8.436862000 0.156632000

H 9.196909000 7.455600000 -1.247776000

C 10.555166000 4.935815000 -1.225928000

H 11.415135000 5.509699000 -1.596501000

H 9.725894000 5.142264000 -1.914147000

H 10.808720000 3.877574000 -1.321648000

C 5.961463000 6.257815000 0.200284000

H 7.052397000 7.816375000 1.354215000

C 4.241033000 4.371788000 -0.241850000

H 3.273101000 4.880424000 -0.354359000

H 4.027618000 3.375722000 0.156794000

H 4.648855000 4.244445000 -1.249470000

C 6.121274000 6.703281000 -1.226300000

H 5.171868000 7.078288000 -1.630639000

H 6.444444000 5.895997000 -1.896366000

H 6.850302000 7.512607000 -1.308827000

C 4.241064000 4.372290000 2.951514000

H 4.027185000 3.376324000 2.552883000

H 3.273333000 4.881224000 3.064419000

H 4.649194000 4.244762000 3.958988000

C 9.513033000 7.743217000 2.950445000

H 8.765087000 8.435958000 2.553919000

H 10.445050000 8.315031000 3.061638000

H 9.198065000 7.455512000 3.958080000

**^U^Int1**

E=-2102.92684985 Ha

Sum of electronic and thermal Enthalpies= -2102.076594 Ha

Sum of electronic and thermal Free Energies= -2102.217978 Ha

U -0.862578000 -0.784711000 1.509569000

C 1.858623000 -0.285489000 1.782668000

C -1.346435000 -1.848265000 -0.999800000

C 0.992049000 -1.731923000 3.360657000

N 1.073481000 1.839910000 -3.236185000

C -1.932285000 -3.302864000 0.664483000

C -0.537554000 -3.465127000 0.402999000

C 1.646203000 -1.651717000 2.104498000

C -2.763875000 0.574175000 3.052695000

C 0.297619000 -4.601383000 0.913659000

H 1.366819000 -4.371415000 0.936891000

H 0.181185000 -5.479454000 0.263121000

H 0.002660000 -4.916175000 1.918661000

C -3.543105000 0.337114000 1.881565000

C -3.531582000 1.295816000 -0.562743000

H -2.921907000 0.857406000 -1.362175000

H -3.605045000 2.369830000 -0.775454000

H -4.539516000 0.884237000 -0.658809000

C -1.738075000 1.486451000 2.703774000

H -1.026371000 1.924393000 3.389261000

C -0.173601000 -2.559618000 -0.626191000

C -2.446951000 -2.342105000 -0.249583000

C 0.827119000 -0.399722000 3.839015000

C -3.889639000 -2.202443000 -0.630779000

H -4.567647000 -2.369840000 0.207950000

H -4.138632000 -2.954032000 -1.392895000

H -4.115052000 -1.225676000 -1.061207000

C -3.145630000 0.120215000 4.433405000

H -3.328696000 -0.960195000 4.493892000

H -4.072777000 0.608376000 4.764043000

H -2.373012000 0.367017000 5.164092000

C 1.370795000 0.488478000 2.873783000

C 1.136858000 -2.509362000 -1.355366000

H 1.175112000 -3.282083000 -2.135005000

H 1.995486000 -2.684755000 -0.699223000

H 1.285516000 -1.547955000 -1.857913000

C 0.070770000 5.334296000 -3.884792000

H -0.844395000 5.885426000 -4.084418000

C 1.289790000 6.007631000 -3.800080000

H 1.334770000 7.084233000 -3.932492000

C 1.178335000 3.221918000 -3.461187000

C -2.967718000 1.073568000 0.811527000

C 2.402862000 3.894678000 -3.376687000

H 3.314788000 3.337237000 -3.191435000

C -4.885785000 -0.332557000 1.921108000

H -4.843405000 -1.358123000 2.306234000

H -5.359863000 -0.370034000 0.938945000

H -5.562701000 0.220938000 2.586005000

C 2.448443000 5.276379000 -3.546805000

H 3.407620000 5.783406000 -3.483274000

C -1.833894000 1.776549000 1.318469000

C -1.012257000 2.771243000 0.545367000

H -0.272607000 3.251770000 1.189708000

H -1.643998000 3.568003000 0.131492000

H -0.474263000 2.337839000 -0.308920000

C 2.657617000 0.250197000 0.626790000

H 2.197210000 1.135558000 0.169683000

H 2.780936000 -0.507403000 -0.152039000

H 3.663304000 0.552791000 0.948598000

C 0.010567000 3.955252000 -3.719343000

H -0.945316000 3.439250000 -3.784303000

C 0.393401000 -0.036621000 5.227866000

H -0.059204000 0.958455000 5.279969000

H 1.251136000 -0.029370000 5.915156000

H -0.329769000 -0.751381000 5.632477000

C 1.651234000 1.948699000 3.069229000

H 2.658176000 2.091174000 3.485731000

H 0.956387000 2.426682000 3.765666000

H 1.617815000 2.508214000 2.129655000

H 0.227552000 1.407883000 -3.582170000

H 1.979725000 -2.493019000 1.513400000

C 0.702569000 -2.975179000 4.155322000

H -0.348854000 -3.064719000 4.459333000

H 0.959666000 -3.872261000 3.589033000

H 1.293742000 -2.995286000 5.080039000

H -1.415563000 -1.151715000 -1.830771000

C -2.749128000 -4.168133000 1.583211000

H -3.670789000 -3.669954000 1.900547000

H -3.046421000 -5.107778000 1.097180000

H -2.197692000 -4.443641000 2.488930000

N 2.169197000 1.007704000 -3.299646000

C 4.929477000 -0.004064000 -5.529116000

H 5.900318000 -0.484635000 -5.441261000

C 4.447377000 0.376901000 -6.781917000

H 5.035801000 0.197923000 -7.676527000

C 2.923832000 0.845221000 -4.468255000

C 2.437819000 1.228782000 -5.724166000

H 1.473792000 1.719322000 -5.805891000

C 3.200688000 0.993030000 -6.865513000

H 2.810667000 1.301306000 -7.831955000

C 4.180522000 0.226445000 -4.381224000

H 4.563362000 -0.078948000 -3.409577000

H 2.672267000 0.934777000 -2.427010000

**^U^TS1**

E=-2102.88056879 Ha

Sum of electronic and thermal Enthalpies= -2102.031984 Ha

Sum of electronic and thermal Free Energies= -2102.160861 Ha

U 6.987100000 3.866742000 19.675978000

C 9.850270000 4.302544000 20.216391000

C 7.249755000 1.860378000 17.757690000

C 8.650880000 3.083559000 21.766221000

N 7.649829000 4.910669000 17.380526000

C 5.247779000 1.669048000 18.850605000

C 6.244714000 0.995080000 19.618860000

C 9.427355000 3.002226000 20.576916000

C 4.573437000 4.449396000 21.063467000

C 5.934511000 0.046866000 20.742074000

H 6.840593000 -0.402223000 21.155863000

H 5.302277000 -0.778744000 20.385759000

H 5.394824000 0.515129000 21.572985000

C 4.197792000 4.876016000 19.760565000

C 4.693290000 6.926748000 18.273540000

H 5.505341000 7.632039000 18.086819000

H 3.789584000 7.517051000 18.485828000

H 4.504445000 6.389257000 17.338662000

C 5.581761000 5.336080000 21.518326000

H 5.995957000 5.330297000 22.515714000

C 7.474358000 1.075976000 18.909705000

C 5.874555000 2.220393000 17.699811000

C 8.614956000 4.455041000 22.149171000

C 5.178790000 2.833107000 16.512321000

H 4.345012000 2.204522000 16.176207000

H 5.862008000 2.913442000 15.660237000

H 4.760633000 3.831150000 16.696067000

C 3.933276000 3.394685000 21.922302000

H 4.377018000 2.399605000 21.803331000

H 2.864243000 3.295705000 21.706342000

H 4.024939000 3.663115000 22.979834000

C 9.340469000 5.208754000 21.189762000

C 8.682806000 0.214037000 19.131052000

H 8.489287000 -0.798884000 18.753329000

H 8.966388000 0.107569000 20.182789000

H 9.549986000 0.594501000 18.582585000

C 7.092160000 8.043389000 15.443772000

H 6.568502000 8.371889000 14.550352000

C 7.839517000 8.952916000 16.190578000

H 7.897592000 9.994319000 15.888110000

C 7.674687000 6.259729000 16.987929000

C 4.992864000 6.009564000 19.420485000

C 8.441219000 7.179963000 17.721817000

H 8.958606000 6.832844000 18.610615000

C 2.952086000 4.506325000 19.010223000

H 2.390371000 3.719160000 19.515474000

H 3.135015000 4.172216000 17.984623000

H 2.292982000 5.382133000 18.940190000

C 8.520880000 8.510822000 17.327143000

H 9.112432000 9.209172000 17.913153000

C 5.838939000 6.310775000 20.524034000

C 6.568304000 7.604167000 20.737363000

H 7.247365000 7.541700000 21.589561000

H 5.852374000 8.409234000 20.953358000

H 7.146945000 7.922299000 19.866341000

C 10.945539000 4.616329000 19.239610000

H 11.086559000 3.804096000 18.521554000

H 11.898969000 4.744030000 19.771358000

H 10.785197000 5.549180000 18.685479000

C 7.021473000 6.706706000 15.825898000

H 6.451321000 6.000184000 15.228710000

C 8.171599000 4.943673000 23.496355000

H 7.828823000 5.982727000 23.485049000

H 9.004499000 4.891259000 24.211579000

H 7.364707000 4.331647000 23.910990000

C 9.810083000 6.625697000 21.357744000

H 10.900206000 6.649823000 21.491551000

H 9.372524000 7.094259000 22.242717000

H 9.584366000 7.278533000 20.508087000

H 6.995688000 4.411919000 16.782151000

H 9.752092000 2.095731000 20.091823000

C 8.210223000 1.940373000 22.636958000

H 7.173481000 2.030356000 22.981762000

H 8.297376000 0.987290000 22.113060000

H 8.836843000 1.874009000 23.536805000

H 8.010921000 2.130514000 17.035206000

C 3.781705000 1.391480000 19.010143000

H 3.181078000 1.920933000 18.269673000

H 3.601219000 0.317158000 18.860939000

H 3.386058000 1.637234000 19.998475000

N 9.135385000 4.131066000 16.549081000

C 10.067516000 5.813976000 13.361471000

H 10.671809000 6.646420000 13.010880000

C 9.378904000 5.016651000 12.446116000

H 9.447311000 5.222447000 11.381760000

C 9.213289000 4.470903000 15.206163000

C 8.528185000 3.670545000 14.265092000

H 7.968575000 2.811047000 14.623240000

C 8.606305000 3.946329000 12.906970000

H 8.079482000 3.313059000 12.198028000

C 9.974661000 5.558165000 14.724868000

H 10.499929000 6.191139000 15.435897000

H 9.798313000 4.695043000 17.075015000

**^U^Int2**

E=-2102.93339998 Ha

Sum of electronic and thermal Enthalpies= -2102.085322

Sum of electronic and thermal Free Energies= -2102.222969

U -0.491065000 -0.255165000 0.975088000

C 2.315420000 0.127018000 1.068455000

C -0.282385000 -1.967956000 -1.202374000

C 1.409792000 -1.075453000 2.825591000

N -0.214859000 1.077472000 -0.850513000

C -2.201432000 -2.433703000 -0.040296000

C -1.125148000 -3.136335000 0.576708000

C 1.964474000 -1.167924000 1.520959000

C -2.711585000 -0.066277000 2.696774000

C -1.294573000 -4.201561000 1.621560000

H -0.332739000 -4.611720000 1.939923000

H -1.885992000 -5.041726000 1.231277000

H -1.809923000 -3.846406000 2.520542000

C -3.236722000 0.506586000 1.500619000

C -2.910434000 2.713658000 0.191284000

H -2.078103000 3.374468000 -0.060476000

H -3.746449000 3.344168000 0.525888000

H -3.227132000 2.223163000 -0.733794000

C -1.713733000 0.813939000 3.176875000

H -1.189630000 0.694474000 4.115017000

C 0.049539000 -2.893596000 -0.182766000

C -1.677106000 -1.694382000 -1.136828000

C 1.447882000 0.293352000 3.195691000

C -2.472371000 -0.984047000 -2.199987000

H -3.247492000 -1.641614000 -2.612317000

H -1.825307000 -0.705336000 -3.038021000

H -2.974348000 -0.073902000 -1.852160000

C -3.242688000 -1.241519000 3.466296000

H -3.233563000 -2.178462000 2.903156000

H -4.280051000 -1.067488000 3.779506000

H -2.655692000 -1.400103000 4.376240000

C 1.999420000 1.037595000 2.113300000

C 1.283689000 -3.747859000 -0.175782000

H 1.080048000 -4.682489000 -0.715028000

H 1.620383000 -4.031819000 0.826752000

H 2.116425000 -3.261443000 -0.692481000

C 0.187100000 3.768651000 -3.376459000

H -0.052444000 3.937768000 -4.423812000

C 0.825774000 4.764389000 -2.636147000

H 1.094466000 5.711123000 -3.094871000

C 0.118629000 2.287253000 -1.427277000

C -2.541285000 1.722077000 1.251404000

C 0.761355000 3.308481000 -0.696849000

H 0.995341000 3.126390000 0.343892000

C -4.536022000 0.162959000 0.834243000

H -4.967279000 -0.757044000 1.229953000

H -4.460997000 0.059734000 -0.252327000

H -5.261305000 0.966187000 1.021047000

C 1.105581000 4.517878000 -1.291214000

H 1.602389000 5.278189000 -0.692832000

C -1.611071000 1.929912000 2.303338000

C -0.946483000 3.238745000 2.608439000

H -0.137595000 3.126992000 3.332080000

H -1.676989000 3.929175000 3.051409000

H -0.547038000 3.729962000 1.718048000

C 3.066869000 0.432445000 -0.193465000

H 2.890018000 -0.333172000 -0.953915000

H 4.148237000 0.467054000 -0.003694000

H 2.776548000 1.394840000 -0.623743000

C -0.160919000 2.556085000 -2.789324000

H -0.671961000 1.799000000 -3.381408000

C 1.261455000 0.809343000 4.590557000

H 0.801769000 1.800942000 4.630647000

H 2.237516000 0.895039000 5.088672000

H 0.656071000 0.133031000 5.201590000

C 2.525138000 2.440647000 2.223617000

H 3.329749000 2.475584000 2.971104000

H 1.778736000 3.176288000 2.535194000

H 2.956057000 2.781157000 1.279231000

H -0.586504000 0.482105000 -1.586018000

H 2.208857000 -2.081224000 1.008330000

C 1.050977000 -2.224207000 3.724875000

H 0.064047000 -2.116280000 4.191153000

H 1.050123000 -3.166906000 3.173181000

H 1.775946000 -2.327644000 4.542932000

H 0.395885000 -1.613891000 -1.974778000

C -3.638398000 -2.824977000 0.141013000

H -4.310777000 -2.211066000 -0.459105000

H -3.767715000 -3.864842000 -0.191464000

H -3.986652000 -2.787057000 1.176137000

N 1.758620000 -0.266323000 -3.639207000

C 3.733038000 1.031866000 -6.474024000

H 4.116356000 1.966680000 -6.874073000

C 3.991408000 -0.171562000 -7.147872000

H 4.573391000 -0.164470000 -8.065015000

C 2.479588000 -0.188700000 -4.762544000

C 2.758669000 -1.399605000 -5.473538000

H 2.366179000 -2.323084000 -5.058802000

C 3.499000000 -1.383397000 -6.639407000

H 3.702485000 -2.311350000 -7.166735000

C 2.994395000 1.033431000 -5.306135000

H 2.785119000 1.962876000 -4.781511000

H 1.642926000 0.672701000 -3.244637000

**4-U**

E=-1816.09419130 Ha

Sum of electronic and thermal Enthalpies= -1815.359274 Ha

Sum of electronic and thermal Free Energies= -1815.475101 Ha

U 6.937083000 3.155769000 14.911392000

C 9.392059000 4.682741000 14.546327000

C 7.181093000 0.520325000 16.241243000

C 8.751741000 4.120011000 16.669798000

H 8.712089000 3.642619000 17.637260000

N 7.940535000 1.742040000 13.441996000

C 5.198611000 1.654746000 16.332670000

H 4.164725000 1.924373000 16.174266000

C 6.017189000 2.145976000 17.382695000

C 9.557903000 3.712093000 15.576347000

C 8.201043000 -0.543847000 15.952925000

H 9.215806000 -0.146266000 15.842946000

H 7.961374000 -1.100036000 15.041114000

H 8.234648000 -1.279613000 16.767452000

C 4.433352000 4.449614000 14.597307000

C 5.572493000 3.053272000 18.493330000

H 6.413545000 3.565644000 18.969855000

H 5.061116000 2.484049000 19.281099000

H 4.872186000 3.818732000 18.147377000

C 4.265702000 3.324221000 13.744073000

C 5.207691000 2.543741000 11.461145000

H 6.164218000 2.583420000 10.936704000

H 4.419660000 2.817772000 10.746321000

H 5.043137000 1.498638000 11.737935000

C 5.441552000 5.260980000 14.028171000

H 5.731195000 6.235036000 14.399201000

C 7.240652000 1.423131000 17.333862000

C 10.570970000 2.606257000 15.546045000

H 10.415004000 1.893023000 16.358471000

H 11.586643000 3.007702000 15.661573000

H 10.544078000 2.053234000 14.602183000

C 5.905447000 0.646622000 15.623814000

C 8.064368000 5.310163000 16.321409000

C 5.356131000 -0.270048000 14.566361000

H 4.283253000 -0.115205000 14.439842000

H 5.495408000 -1.318654000 14.855579000

H 5.830414000 -0.138434000 13.587062000

C 3.555589000 4.831468000 15.752448000

H 3.251813000 3.973427000 16.360643000

H 2.631963000 5.306595000 15.395812000

H 4.049537000 5.550935000 16.412837000

C 8.488393000 5.673576000 15.011160000

C 8.280221000 1.367855000 18.412516000

H 8.152016000 0.450749000 19.003490000

H 8.200750000 2.203425000 19.113533000

H 9.303897000 1.351642000 18.026729000

C 9.834499000 -0.079583000 10.817734000

H 10.240110000 -1.075906000 10.658478000

C 9.992189000 0.902996000 9.839756000

H 10.517209000 0.685601000 8.914483000

C 8.614958000 1.475752000 12.259320000

C 5.179253000 3.446387000 12.658622000

C 8.789094000 2.455655000 11.261352000

H 8.393017000 3.448172000 11.432806000

C 3.104208000 2.376078000 13.769203000

H 2.743068000 2.158694000 14.778603000

H 3.331792000 1.426141000 13.280236000

H 2.257617000 2.816292000 13.224377000

C 9.462092000 2.170964000 10.077528000

H 9.574685000 2.954703000 9.331918000

C 5.896646000 4.664189000 12.817350000

C 6.714450000 5.341193000 11.751811000

H 7.783949000 5.399767000 11.972110000

H 6.362178000 6.367944000 11.601299000

H 6.608035000 4.823035000 10.795051000

C 10.261992000 4.781151000 13.328848000

H 10.464865000 3.805953000 12.880357000

H 11.231179000 5.225832000 13.595470000

H 9.828411000 5.418796000 12.553109000

C 9.161496000 0.197733000 12.001789000

H 9.050901000 -0.583732000 12.751307000

C 7.239204000 6.163906000 17.240649000

H 6.339213000 6.559902000 16.757120000

H 7.814796000 7.030259000 17.592686000

H 6.920699000 5.608650000 18.126131000

C 8.270550000 7.022333000 14.393207000

H 9.119342000 7.677992000 14.631106000

H 7.377091000 7.515866000 14.785751000

H 8.186806000 6.994540000 13.305041000

H 7.950475000 0.886135000 13.991534000

**1-Pu**

E=-1606.19436496 Ha

Sum of electronic and thermal Enthalpies= -1605.574000 Ha

Sum of electronic and thermal Free Energies= -1605.676667 Ha

Pu 7.839688000 4.529805000 1.354809000

C 6.490413000 2.178634000 1.354756000

C 7.306468000 2.078257000 2.509618000

C 8.645552000 1.890984000 2.066814000

C 8.645476000 1.891074000 0.642437000

C 7.306347000 2.078401000 0.199799000

C 6.840466000 2.001658000 3.936940000

C 9.785045000 1.470081000 2.946355000

C 9.784862000 1.470288000 -0.237300000

C 6.840218000 2.001990000 -1.227492000

H 5.413879000 2.279570000 1.354795000

H 5.766759000 2.186783000 4.011560000

H 7.345905000 2.718060000 4.597921000

H 7.028110000 1.006145000 4.359503000

H 10.758225000 1.803646000 2.573766000

H 9.834247000 0.374202000 3.015331000

H 9.673897000 1.844875000 3.968270000

H 9.833755000 0.374428000 -0.306815000

H 10.758128000 1.803384000 0.135479000

H 9.673850000 1.845611000 -1.259038000

H 7.028083000 1.006616000 -1.650284000

H 7.345398000 2.718661000 -1.888384000

H 5.766455000 2.186855000 -1.301961000

C 10.552623000 4.539652000 1.354622000

C 10.230917000 5.295968000 2.509536000

C 6.479052000 6.872464000 1.354669000

C 5.985731000 6.215040000 2.509549000

C 9.722896000 6.549006000 2.067190000

C 10.532124000 4.929831000 3.935990000

H 10.818525000 3.879512000 4.023270000

H 9.684034000 5.099655000 4.611770000

H 11.365192000 5.527171000 4.329361000

C 9.722791000 6.549113000 0.642475000

C 5.155725000 5.147106000 2.066965000

C 6.153999000 6.654680000 3.937097000

H 6.877164000 7.469233000 4.017062000

H 6.490106000 5.847357000 4.600947000

H 5.205444000 7.020383000 4.351357000

C 5.155905000 5.146968000 0.642368000

C 10.230706000 5.296125000 0.199872000

H 11.006271000 3.558003000 1.354489000

C 9.514330000 7.743595000 -0.240117000

H 10.445060000 8.320564000 -0.334144000

H 8.756939000 8.432764000 0.145170000

H 9.217834000 7.456952000 -1.253597000

C 10.531609000 4.930146000 -1.226685000

H 11.364526000 5.527597000 -1.620204000

H 9.683343000 5.099951000 -1.902252000

H 10.818086000 3.879859000 -1.314115000

C 5.986108000 6.214748000 0.199796000

H 7.103136000 7.755620000 1.354599000

C 4.229063000 4.364957000 -0.240339000

H 3.265410000 4.883404000 -0.343000000

H 4.008159000 3.367793000 0.151876000

H 4.630281000 4.241952000 -1.250911000

C 6.154925000 6.654028000 -1.227796000

H 5.206515000 7.019557000 -1.642542000

H 6.491370000 5.846565000 -1.891307000

H 6.878063000 7.468619000 -1.307660000

C 4.228710000 4.365203000 2.949580000

H 4.007890000 3.367980000 2.557457000

H 3.265033000 4.883656000 3.051976000

H 4.629716000 4.242342000 3.960255000

C 9.514499000 7.743353000 2.949977000

H 8.756595000 8.432244000 2.565202000

H 10.445055000 8.320681000 3.043506000

H 9.218708000 7.456501000 3.963602000

**^Pu^Int1**

E=-2179.94464852 Ha

Sum of electronic and thermal Enthalpies= -2179.093998 Ha

Sum of electronic and thermal Free Energies= -2179.233687 Ha

Pu -0.787676000 -0.624236000 1.533931000

C 1.923636000 -0.329675000 1.135944000

C -0.629188000 -1.869716000 -0.925653000

C 1.349217000 -1.068629000 3.246148000

N 1.394834000 0.441625000 -4.082929000

C -2.411281000 -2.589194000 0.321930000

C -1.252404000 -3.315179000 0.731065000

C 1.728417000 -1.480488000 1.944245000

C -2.856666000 -0.032837000 3.307604000

C -1.257684000 -4.461728000 1.701773000

H -0.269070000 -4.633435000 2.139251000

H -1.560471000 -5.401207000 1.218028000

H -1.957041000 -4.296475000 2.528628000

C -3.529118000 0.277804000 2.092745000

C -3.336561000 2.167804000 0.301282000

H -2.513384000 2.467093000 -0.357204000

H -3.838417000 3.093703000 0.616384000

H -4.052015000 1.606560000 -0.303950000

C -1.825810000 0.926455000 3.479574000

H -1.197890000 1.012753000 4.355742000

C -0.166686000 -2.918546000 -0.090670000

C -2.022540000 -1.683112000 -0.704398000

C 1.351665000 0.353035000 3.263381000

C -2.924229000 -0.873362000 -1.590747000

H -3.832484000 -0.548086000 -1.078460000

H -3.241022000 -1.461000000 -2.463597000

H -2.421969000 0.021242000 -1.972699000

C -3.273591000 -1.061369000 4.321536000

H -3.540498000 -2.022463000 3.866860000

H -4.150193000 -0.729544000 4.893885000

H -2.474158000 -1.250094000 5.045658000

C 1.689542000 0.808418000 1.958511000

C 1.098494000 -3.704247000 -0.269579000

H 0.948635000 -4.463604000 -1.048790000

H 1.391985000 -4.244393000 0.636140000

H 1.948617000 -3.093739000 -0.582433000

C -1.081339000 3.131235000 -3.588812000

H -2.131263000 3.309158000 -3.805892000

C -0.329607000 4.101289000 -2.923665000

H -0.785042000 5.038188000 -2.618298000

C 0.855665000 1.679997000 -3.723207000

C -2.866869000 1.384270000 1.493005000

C 1.614480000 2.655066000 -3.061727000

H 2.668300000 2.481513000 -2.872426000

C -4.906005000 -0.192535000 1.731331000

H -5.146269000 -1.158710000 2.178537000

H -5.072512000 -0.270140000 0.653401000

H -5.645831000 0.526552000 2.110343000

C 1.017733000 3.851505000 -2.669160000

H 1.622918000 4.599947000 -2.164375000

C -1.825460000 1.806172000 2.365108000

C -1.118218000 3.123169000 2.235909000

H -0.337561000 3.239414000 2.989313000

H -1.828781000 3.948838000 2.380334000

H -0.658709000 3.274600000 1.253471000

C 2.524094000 -0.251341000 -0.238920000

H 2.414668000 -1.188218000 -0.788896000

H 3.599642000 -0.029830000 -0.178694000

H 2.069097000 0.542739000 -0.841891000

C -0.499971000 1.932761000 -3.988021000

H -1.095073000 1.183827000 -4.506490000

C 1.301560000 1.189297000 4.507142000

H 0.832143000 2.164991000 4.354614000

H 2.318026000 1.379731000 4.878290000

H 0.762646000 0.686254000 5.316217000

C 2.050689000 2.211227000 1.561732000

H 3.139262000 2.305459000 1.444422000

H 1.747232000 2.942758000 2.312747000

H 1.606112000 2.514545000 0.607098000

H 0.869243000 -0.105279000 -4.750368000

H 1.908214000 -2.503646000 1.652021000

C 1.139517000 -1.976323000 4.425617000

H 0.286961000 -1.676697000 5.046554000

H 0.966379000 -3.008131000 4.103788000

H 2.017759000 -1.989085000 5.084718000

H -0.049261000 -1.373915000 -1.698109000

C -3.804186000 -3.024604000 0.669215000

H -4.559141000 -2.372292000 0.230361000

H -3.984874000 -4.034610000 0.276410000

H -3.991312000 -3.071672000 1.747408000

N 2.743403000 0.193718000 -4.085039000

C 5.917834000 1.295274000 -5.603306000

H 6.982119000 1.191376000 -5.408905000

C 5.476110000 2.006039000 -6.719882000

H 6.188696000 2.460396000 -7.401335000

C 3.629097000 0.833883000 -4.956108000

C 3.182765000 1.547471000 -6.075640000

H 2.118905000 1.656663000 -6.257497000

C 4.106104000 2.125155000 -6.943864000

H 3.742864000 2.678868000 -7.805685000

C 5.008923000 0.714338000 -4.726857000

H 5.363950000 0.158246000 -3.861620000

H 3.111934000 -0.105704000 -3.193760000

**^Pu^TS1**

E=-2179.88658518 Ha

Sum of electronic and thermal Enthalpies= -2179.039278 Ha

Sum of electronic and thermal Free Energies= -2179.169989 Ha

Pu 6.942059000 3.778099000 19.742801000

C 9.827641000 4.263391000 20.124090000

C 7.162993000 1.731548000 17.865786000

C 8.689758000 3.121341000 21.773782000

N 7.479242000 4.824653000 17.422628000

C 5.176407000 1.565164000 18.992818000

C 6.182710000 0.913887000 19.763387000

C 9.418558000 2.982365000 20.561815000

C 4.568066000 4.466658000 21.106291000

C 5.897427000 0.022276000 20.937967000

H 6.812358000 -0.414978000 21.345577000

H 5.247019000 -0.813286000 20.642721000

H 5.387876000 0.535310000 21.762007000

C 4.162893000 4.837278000 19.795507000

C 4.632450000 6.818696000 18.209789000

H 5.443516000 7.512121000 17.977600000

H 3.735520000 7.423731000 18.409340000

H 4.423130000 6.239474000 17.304069000

C 5.595220000 5.365466000 21.497600000

H 6.030376000 5.405409000 22.485586000

C 7.404943000 0.983610000 19.041846000

C 5.788210000 2.083078000 17.815938000

C 8.660453000 4.508974000 22.088529000

C 5.083012000 2.638843000 16.607488000

H 4.287219000 1.964253000 16.266848000

H 5.783480000 2.732474000 15.770044000

H 4.613681000 3.618345000 16.765149000

C 3.941729000 3.455251000 22.025113000

H 4.359907000 2.446600000 21.924875000

H 2.863320000 3.369882000 21.853530000

H 4.079182000 3.757112000 23.068643000

C 9.356203000 5.214738000 21.069888000

C 8.614668000 0.125521000 19.273830000

H 8.410958000 -0.902433000 18.944703000

H 8.923530000 0.063263000 20.322400000

H 9.471574000 0.477148000 18.690770000

C 7.076095000 7.804104000 15.255059000

H 6.555959000 8.098027000 14.347720000

C 7.878041000 8.725113000 15.930181000

H 7.982331000 9.738569000 15.553914000

C 7.586227000 6.102390000 16.926017000

C 4.949969000 5.954173000 19.390504000

C 8.414789000 7.040985000 17.579146000

H 8.922368000 6.726030000 18.484044000

C 2.898966000 4.439250000 19.092286000

H 2.350348000 3.671971000 19.640823000

H 3.057463000 4.064611000 18.076576000

H 2.236397000 5.310942000 19.003364000

C 8.551274000 8.333105000 17.091038000

H 9.183401000 9.042469000 17.618352000

C 5.828162000 6.295349000 20.455696000

C 6.574543000 7.588361000 20.590075000

H 7.262522000 7.562957000 21.437217000

H 5.872713000 8.415044000 20.768307000

H 7.148546000 7.851121000 19.696956000

C 10.853135000 4.528237000 19.062410000

H 11.049349000 3.628423000 18.469990000

H 11.811305000 4.818969000 19.515913000

H 10.581112000 5.341784000 18.379492000

C 6.939713000 6.506843000 15.733705000

H 6.307716000 5.795761000 15.209240000

C 8.257528000 5.065278000 23.422055000

H 7.902659000 6.098929000 23.365590000

H 9.111857000 5.059758000 24.113753000

H 7.469541000 4.469975000 23.893953000

C 9.830762000 6.637434000 21.159610000

H 10.927723000 6.670381000 21.213265000

H 9.455737000 7.129332000 22.060943000

H 9.539570000 7.267557000 20.312765000

H 6.832761000 4.295044000 16.843280000

H 9.723854000 2.053054000 20.107672000

C 8.256206000 2.019744000 22.698946000

H 7.229439000 2.140360000 23.065195000

H 8.314416000 1.046081000 22.209521000

H 8.901947000 1.976166000 23.586549000

H 7.891057000 1.959153000 17.095732000

C 3.711178000 1.309513000 19.190341000

H 3.104416000 1.805429000 18.431280000

H 3.515218000 0.230981000 19.103944000

H 3.334171000 1.615133000 20.169807000

N 8.955863000 3.766437000 16.206358000

C 10.506166000 6.178671000 13.859447000

H 11.281626000 6.939126000 13.831497000

C 9.776530000 5.884833000 12.703540000

H 9.988603000 6.414022000 11.778806000

C 9.243758000 4.495730000 15.098040000

C 8.506673000 4.226863000 13.912404000

H 7.750182000 3.448291000 13.952381000

C 8.775807000 4.906612000 12.736325000

H 8.214020000 4.675960000 11.835359000

C 10.241540000 5.506258000 15.042803000

H 10.812177000 5.730265000 15.939727000

H 9.612555000 3.993201000 16.951346000

**^Pu^Int2**

E=-2179.91470858 Ha

Sum of electronic and thermal Enthalpies= -2179.067338 Ha

Sum of electronic and thermal Free Energies= -2179.207408 Ha

Pu -0.474454000 -0.184757000 0.927842000

C 2.303260000 0.182425000 1.031521000

C -0.245376000 -1.918729000 -1.232167000

C 1.412512000 -1.050553000 2.780447000

N -0.207113000 1.182929000 -0.882820000

C -2.148430000 -2.428365000 -0.063231000

C -1.063261000 -3.120441000 0.544369000

C 1.971597000 -1.121950000 1.476013000

C -2.676720000 -0.121643000 2.697894000

C -1.214291000 -4.206901000 1.569599000

H -0.247495000 -4.634599000 1.847633000

H -1.827706000 -5.032199000 1.181479000

H -1.694158000 -3.864939000 2.492888000

C -3.229305000 0.453070000 1.510529000

C -2.983659000 2.693031000 0.235434000

H -2.161802000 3.357501000 -0.041547000

H -3.807672000 3.320481000 0.604428000

H -3.333245000 2.208820000 -0.680953000

C -1.709160000 0.779884000 3.182343000

H -1.178734000 0.667001000 4.117009000

C 0.102611000 -2.852734000 -0.219506000

C -1.637474000 -1.665281000 -1.157134000

C 1.429545000 0.314532000 3.160008000

C -2.452646000 -0.955993000 -2.204253000

H -3.197808000 -1.631808000 -2.642334000

H -1.812959000 -0.623495000 -3.027950000

H -2.993506000 -0.079044000 -1.830992000

C -3.174795000 -1.318792000 3.453177000

H -3.159172000 -2.242532000 2.869475000

H -4.209352000 -1.169070000 3.788401000

H -2.568195000 -1.486151000 4.348414000

C 1.965661000 1.078696000 2.077668000

C 1.342812000 -3.696586000 -0.239026000

H 1.139845000 -4.624059000 -0.791260000

H 1.690987000 -3.994229000 0.755503000

H 2.166431000 -3.195895000 -0.756644000

C 0.149840000 3.838008000 -3.453103000

H -0.052812000 3.975654000 -4.512758000

C 0.698577000 4.881521000 -2.705864000

H 0.932782000 5.833733000 -3.172164000

C 0.081317000 2.390168000 -1.478301000

C -2.574044000 1.693540000 1.273144000

C 0.634811000 3.460818000 -0.741585000

H 0.835167000 3.308351000 0.311581000

C -4.533817000 0.093107000 0.865120000

H -4.935230000 -0.845894000 1.247099000

H -4.479642000 0.019435000 -0.225304000

H -5.273707000 0.874103000 1.086718000

C 0.934705000 4.676627000 -1.345110000

H 1.361739000 5.475523000 -0.743256000

C -1.644097000 1.912699000 2.320599000

C -1.027943000 3.239560000 2.645930000

H -0.213185000 3.148558000 3.366051000

H -1.782402000 3.895535000 3.101542000

H -0.651797000 3.761457000 1.762413000

C 3.047805000 0.511418000 -0.227692000

H 2.882874000 -0.250327000 -0.994594000

H 4.128859000 0.564394000 -0.039574000

H 2.738636000 1.472360000 -0.648604000

C -0.153939000 2.618417000 -2.857435000

H -0.595895000 1.822808000 -3.454439000

C 1.255484000 0.817416000 4.560497000

H 0.781221000 1.801456000 4.614939000

H 2.238229000 0.915525000 5.043168000

H 0.671196000 0.126592000 5.175465000

C 2.445441000 2.496555000 2.184620000

H 3.278426000 2.554488000 2.899246000

H 1.684511000 3.198188000 2.536105000

H 2.822590000 2.863623000 1.227402000

H -0.500323000 0.541318000 -1.617176000

H 2.237424000 -2.026240000 0.958593000

C 1.090604000 -2.210876000 3.677162000

H 0.115749000 -2.112474000 4.168456000

H 1.078822000 -3.147770000 3.116026000

H 1.839708000 -2.318213000 4.473050000

H 0.426638000 -1.553060000 -2.004654000

C -3.576830000 -2.852466000 0.101924000

H -4.263507000 -2.224777000 -0.466593000

H -3.687086000 -3.878049000 -0.279407000

H -3.919593000 -2.872779000 1.139716000

N 1.799997000 -0.173749000 -3.659269000

C 3.949089000 1.019758000 -6.412042000

H 4.380675000 1.936079000 -6.805610000

C 4.202120000 -0.201342000 -7.055468000

H 4.828327000 -0.226285000 -7.942666000

C 2.575898000 -0.135267000 -4.747517000

C 2.850301000 -1.364831000 -5.427602000

H 2.408851000 -2.269264000 -5.020228000

C 3.647204000 -1.389404000 -6.555359000

H 3.846767000 -2.331167000 -7.059135000

C 3.154582000 1.061878000 -5.282303000

H 2.949849000 2.005429000 -4.781625000

H 1.696894000 0.773765000 -3.282049000

(PhHN)2

E=-573.766047083 Ha

Sum of electronic and thermal Enthalpies= -573.537999 Ha

Sum of electronic and thermal Free Energies= -573.589175 Ha

N 0.467281000 -0.508273000 -0.724617000

N -0.467002000 0.508047000 -0.724713000

C 1.766559000 -0.218000000 -0.292437000

C -1.766205000 0.217893000 -0.292183000

C 2.814354000 -1.072649000 -0.666024000

C -2.814159000 1.072166000 -0.666178000

C 2.039344000 0.878087000 0.535709000

C -2.038744000 -0.877684000 0.536723000

C 4.106894000 -0.837101000 -0.210284000

C -4.106603000 0.836778000 -0.210077000

C 3.339076000 1.102472000 0.981217000

C -3.338382000 -1.101930000 0.982573000

C 4.382094000 0.252507000 0.615924000

C -4.381551000 -0.252316000 0.616890000

H 0.417824000 -1.136781000 -1.520461000

H -0.417769000 1.136166000 -1.520884000

H 2.611162000 -1.924537000 -1.312134000

H -2.611167000 1.923642000 -1.312896000

H 1.227107000 1.537013000 0.822847000

H -1.226392000 -1.536337000 0.824162000

H 4.905836000 -1.510252000 -0.509992000

H -4.905666000 1.509640000 -0.510111000

H 3.535308000 1.956638000 1.624253000

H -3.534417000 -1.955697000 1.626198000

H 5.393042000 0.436515000 0.966574000

H -5.392424000 -0.436206000 0.967819000
