# Supplementary material for: Plutonium(III) versus uranium(III) and samarium(III) in small molecule activation chemistry
Source: Nat Commun. 2026 May 2;17:5956. doi: 10.1038/s41467-026-72229-7 (PMC13342291; doi:10.1038/s41467-026-72229-7)
Supplement: Supplementary file 2 — Transparent Peer Review file [file 41467_2026_72229_MOESM2_ESM.pdf]

nature portfolio

Peer Review File

Plutonium(III) versus uranium(III) and samarium(III) in small molecule activation chemistry

Corresponding Author: Professor Polly Arnold

This file contains all reviewer reports in order by version, followed by all author rebuttals in order by version.

Version 0:

Reviewer comments:

Reviewer #1

(Remarks to the Author)
This manuscript reports the synthesis and structural characterization of a Pu(III) organometallic complex and its distinctive reactivity in small-molecule bond cleavage. By combining experiment with DFT, the authors systematically compare the behavior of Pu(III), U(III), and Sm(III) complexes and propose a cooperative mechanism involving metal-centered reduction and sterically induced reduction (SIR). The study is original and important for understanding small-molecule activation by transuranium elements.
This work is, in principle, suitable for publication in Nature Communications. However, the theoretical calculations are insufficient and the computational results fail to satisfactorily explain the experimental results. Therefore, I recommend that the manuscript be reconsidered after revision. My detailed comments are as follows.
1. Computational methodology is described incompletely. Please specify, for each element, the effective core potential (ECP) and corresponding basis set employed, and state whether and how solvent effects were included in the calculations.
2. Please provide full TDDFT-simulated UV-Vis-NIR spectra (with oscillator-strength) rather than listing a few individual excitations, to enable direct comparison with the experimental band shapes. In the manuscript, the main band of 1-Pu is assigned as 5f→6d (λmax ≈ 632 nm), whereas for 3-U/4-U it is written as 6d→5f (λmax ≈ 422/398 nm). How should the "6d→5f transition" be interpreted? To facilitate the assignment of transition character, annotate Tables S2–S5 with the dominant MO character for each principal excitation (e.g., U 5f/6d, Pu 5f/6d, Cp π, etc.).
3. Regarding the 5f–5f transitions, some of the results listed in Tables S2–S5 extend beyond the experimental spectral range. The limitations of TDDFT in treating strong correlation and spin–orbit coupling, as well as its unreliable prediction of intensities for Laporte-forbidden transitions, may compromise the conclusions. It is recommended that representative species be investigated using NEVPT2 or CASPT2 (including SOC) for 5f–5f transitions, and that simulated spectra be compared with the experimental data to strengthen the reliability of the assignments.
4. In Fig. 3a, the Pu spin density remains ≈5 (consistent with an f5 configuration). Please discuss whether Int.2Pu can be reasonably classified as Pu(IV), and why the Pu spin density appears essentially unchanged in TS.1Pu. The oxidation state labels and the spin density shown in the figure are contradictory. Re-examine the TS structure and computational reliability as needed. At present, spin-density evolution is shown only for Fig. 3a, please provide analogous data for Fig. 3b and Fig. 4a,b.
5. There appears to be a contradiction in the proposed mechanism for small-molecule activation by 1-Pu and 1-U. At present, the mechanisms are essentially identical except for the SIR step. However, based on the spin density analysis, the oxidation state of Pu in 1-Pu does not change during the reaction, whereas U in 1-U is oxidized to the +4 state. This raises the question of whether the two systems may actually follow different mechanistic pathways.
6. The key SIR step from Int.2Pu to 2-Pu is not explicitly shown in Fig. 3a. Please provide the corresponding transition state, barrier, and reaction energy for this step. In addition, the fate of PhS• in the final conversion from Int.2Pu to 2-Pu is not clearly indicated. Could the authors clarify whether PhS• recombines to form PhS–SPh, thereby contributing to the μ-S–S(Ph) bridge in 2-Pu, or whether one (or both) fragments are released as byproducts? It would be helpful to include this clarification in the figure to ensure conservation of species and atomic balance.
7. In Fig. 3b and Fig. 4a, the purpose of the final step from Int.2U to 3-U, and from Int.4U to 4-U, is unclear. The uranium product does not show any changes from Int.2U to 3-U (or from Int.4U to 4-U), so how can a pronounced decrease in the system's energetics of this step occur? The author should explain how the energetics (i.e., -33.4, -50.7 and -42.7, -57.7) is calculated and the fates of the PhS radical and PhN radical?
8. In Fig. 3, please use dashed bonds to indicate bonds being broken or formed in transition-state structures to improve clarity.

9. The failure to locate a stable [SmIII(CpMe4)3(PhSSPh)] adduct or transition state is only briefly discussed. Please provide a more systematic analysis from both steric and electronic perspectives and assess whether the outcome may be limited by methodological choices (functional, dispersion and solvent models, initial guesses, convergence criteria, etc.).
10. There are inconsistencies between figure/table numbers and citations (e.g., p.4 L96: Figures S17–S19; L100: Table S1; p.6 L162: Table S2; p.8 L201: Tables S3–S4). Please verify and standardize all numbering and cross-references throughout the manuscript.

Reviewer #2

(Remarks to the Author)
In the submitted manuscript, Keener, Rajeshkumar, and co-workers present a study of small molecule activation by a set of related cyclopentadienyl-ligated Pu, U, and Sm complexes. All metals begin in the formal +III oxidation state, placing this work in one typical sweet spot for organometallic systems of the f-block. The study can be distinguished, as demonstrated by a thorough literature section, as a quite uncommon study of small molecule activation at a Pu complex. Work of this type is scarcely conducted with transuranium elements, and thus it is quite notable.

In terms of technical quality, the work has been carried out to a very high standard. Spectroscopic work, XRD, and expert computational work has been utilized to answer the right questions in this work. I am particularly keen on the thorough and well-documented UV-vis-NIR spectra, which seem to be perennially overlooked or abused in literature studies of TRU compounds. I have very few suggestions or questions for the authors about their data—although a few minor points that I'd appreciate seeing addressed in minor revisions are given below in a list of all my specific points.

In terms of broad interest, I anticipate that the molecular mechanistic work presented here brings the paper over the bar for consideration/publication by Nature Communications, the current journal to which it has been submitted. I enjoyed seeing the concepts of SIR, electron transfer, and metal coordination dealt with in an even-handed way. Clearly, SIR is not yet well understood, and Pu still holds many secrets close that are yet waiting to be revealed.

To conclude, this is a solid paper on the chemistry of a novel Pu complex with comparisons to relevant U and Sm analogues. I suggest that it will be suitable for publication in Nature Comms after a round of minor revisions, with my specific points for the authors' consideration given below.

Otherwise, I thank the authors for their nice work and look forward to seeing it in print in due course.

-James Blakemore, University of Kansas

Specific points

1. How was the yield of the crystals of 1-Pu determined? Please mention this. Counting statistics or weighing mass?

2. How was the stability of 1-Pu in solution in toluene for "at least two month" determined? It could be useful to provide this data in the SI, given the observation of a hydrolysis product with bridging OH groups. One might hope for continuous spectral data over time, or periodic spectral data, but if it is manual checks by eye over time, so be it. I think it should just be mentioned how this was estimated.

3. Above Figure 1, the authors compare the out of plane distortion of the methyl groups in 1-Pu and U(Cp*)3 and suggest that metrics in this regard suggest that 1-Pu is poised for SIR. Can this be unpacked a bit more in expanded or clarifying text? Superficially, the similarity of 1-U and 1-Pu suggests to me that these compounds are structurally simply quite similar.

4. The authors are commended for their thorough work on the UV-vis-NIR spectral data collection. Having both the solid and solution data is a real treat! Nothing to change in the data or presentation, I just mention it is lovely.

5. Along the lines of the above, would the authors be willing to mention in their paper any available information regarding the following:

The 5f to 6d excitation in the Pu compounds appears quite distinctive, but I also see that the U compounds have absorption that is not dissimilar in intensity and energy. Are the U compounds showing similar signature of 5f to 6d transitions? This could be interesting to mention…very distinctive and one supposes these are largely driving the beautiful colors of these compounds which as shown in the SI photos accompanying spectra, quite intense.

I see that the 5f to 6d for the U species is mentioned on p.8. However, would the authors be willing to consider mentioning if the relative energies are indicating something about the f and d orbital energies for these compounds relative to the standard view of the 6d and 5f relative energies in standard discussions. The compounds here are analogous and I am interested in how the new spectra can be used to bolster understanding of where 6d to 5f features can be expected in a general sense.

6. The authors mention "slight" decomposition of (PhNH)2 to azobenzene and other products upon mixing with 1-Pu for 14 days. What is the estimated conversion of the starting material to azobenzene, particularly based on apparent integration of the (PhNH)2 in NMR? (I am just asking for an estimate to be included in the final paper, and realize there might be issues that make the quantification less certain than desirable.)

7. In the Conclusion, the authors refer to Pu(III) as weakly reducing. I realize this is subjective language and at the authors discretion, but I personally do not consider Pu(III) to be weakly reducing but rather reasonably strongly reducing, at least when electrolytically produced in basic aqueous solution. Albeit it is less reducing than U(III) to the best of my knowledge. A general statement somewhere in the paper (or multiple places if relevant) about these relative notions and any mention of standard reduction potentials could be helpful to readers who are not experts in Pu chemistry to parse the results shown here.

8. Would the authors consider providing machine readable XYZ files for the computed structures with their paper? I always find these nice as they can be readily loaded into visualization programs, like CIFs.

Reviewer #3

(Remarks to the Author)
Keener and co-workers reports small molecule activation by more or less sterically congested organoactinide complexes such as [An(CpMe4)3] (An = Pu, U), where the authors claims that the Pu complex exhibits a unique reactivity which has not been observed in the U analogue despite many structural similarities found between them. Syntheses and structural characterization for these compounds and reaction products have been thoroughly performed. Therefore, this work should be eligible to be publicized to a scientific community.
However, I still have several concerns in this manuscript as follows.
I do not know what is the novelty of this work, especially for the reductive cleavage of the S-S bond. This reaction is quite ordinary as exemplified by the well-known cystine-cysteine conversion in biochemistry. Needless to say, the thiol (-SH) groups of cysteines oxidatively forms the S-S (disulfide) bond to make cystine, which is reductively cleaved to return to the parent cysteines. In any systems of U and Pu of this work, the final product of (PhS)2 is the same thiophenolates, right? The difference between these actinides is observed solely in the molecular structures of the final coordination compounds, that is, 2-Pu and 3-U, in which thiophenolate(s) are commonly involved. Even if the reported small molecule activations is still novel in the organoplutonium chemistry, I wonder what role the Pu(III) center actually plays there. Considering the current reaction in the simplest manner, one of CpMe4^- ligands in the starting Pu complex (1-Pu) could be a reductant for (PhS)2 to afford thiophenolate and (CpMe4)2, where the Pu(III) center is just spectating due to its weaker reducing nature compared with U(III). According to the synthesis section, KCpMe4 is available in the authors' laboratory. What happens if (PhS)2 is mixed with KCpMe4? To reveal the difference in redox potentials of these trivalent actinide complexes, cyclic voltammetry must be done. For the 1H NMR spectra of Fig. S10 and related discussion in page 5, I wonder that how much (PhS)2 was actually consumed in this reaction. From the peak intensities in the NMR spectra, it is hard to know whether or not > 70% of (PhS)2 has been actually converted in this 7-day reaction, in which 1-Pu has fully disappeared. By the way, which is correct as a yield of 2-Pu, 70% in main text or 81% in SI? Regarding the DFT calculations and Fig. 3 at page 9, what happens if "3-Pu", [Pu(CpMe4)3SPh], is assumed as a final product in Fig. 3(a)? Its stability must be compared with that of 2-Pu, otherwise it is impossible to claim the uniqueness of observed reactivity in this Pu system. This is also the case for Fig. 3(b). Is "2-U", [(U(CpMe4)2)2(mu-SPh)2], less-stable compared with 3-U? First of all, reaction kinetics should be studied experimentally in order to make such a mechanistic discussion, especially to know a rate-determining step of a reaction of interest. Even at the end of discussion, crucial differences and uniqueness of Pu compared with U and Sm still remain unclear. Excuse me, the last 3 sentences are rather equivocal, because any terms of "new", "safe", "important", and "target" are unclear and not fully supported in this work.

I also have several minor concerns listed below.
Page 2, line 41-43. It is confusing. What is an actual ligand in this episode of Sm? CpMe5 or CpMe4? In accordance with Scheme 1, CpMe4 would be correct, right?
Page 5, line 121. I do not know why such a conclusion can be led. More detailed discussion or rationale must be described.
Page 8, line 209. There is no “Scheme 1b” in this manuscript.
Page 10, line 247. Does a single(CpMe4) occur as a byproduct?

Version 1:

Reviewer comments:

Reviewer #1

(Remarks to the Author)
The authors have adequately addressed the concerns raised in my initial review. The revised manuscript improves the presentation of computational methodology, strengthens the TDDFT spectral analysis, and enhances the consistency of the mechanistic illustrations. Overall, the computational results are well supported and show clear agreement with the experimental data. I recommend the manuscript for publication in this journal.

Reviewer #2

(Remarks to the Author)
The authors appear to have fully responded to all of my comments, and carefully responded to those of the other reviewers as well. (I am not a computational chemist and therefore cannot comment on the detailed points raised in that realm, however.) I recommend this article be moved along toward publication once the computational replies have been checked and those details confirmed.

Reviewer #3

(Remarks to the Author)
Regarding my first concern about novelty of the current work, it is a pity that little was eventually answered. I'm aware that this is the first report that TRU molecules have been used to study redox-mediated activation and reduction of small molecules. But, I had to think "so what?", because exploring such scientific frontiers is quite ordinary. While I understood how the authors are passionate to this chemistry, I cannot still feel any new and strong impacts from this work in terms of both science and technology. As I pointed in Comment 3 and the authors actually agreed, the Pu(III) center does not prominently work for the S-S bond cleavage of (PhS)2 with the reducing (CpMe4^-) ligand. Regarding Comment 6 for DFT calculations, the authors' point seems to be wrong. The energy value (30.3 kcal/mol) of the intermediate state labeled "PuInt4" consists of both the Pu(III) species and PhS• radical, right? Please, be sure that it is not "3-Pu" of Pu(IV), and cannot be compared with 3-U of U(IV). In addition, the PhS• radical should be excluded from the energy calculation. In summary, I would think this work is suitable for publication from a more specialized journal after additional revision.

Additional comments to R1:
Comment 4. I'm happy to hear the authors tested the reaction between KCpMe4 and (PhS)2. Did you finally observe the S-S bond cleavage even under absence of any metals? The NMR spectra related to the discussion at line 145-147 should be included in Supporting Information.
Comment 5. If the (PhS)2 loadings exceeded the desired one, it is impossible to write "0.5 equiv. of (PhS)2" in the figure caption. The authors should calculate the actual amount of (PhS)2 in the NMR sample.

Open Access This Peer Review File is licensed under a Creative Commons Attribution 4.0 International License, which permits use, sharing, adaptation, distribution and reproduction in any medium or format, as long as you give appropriate credit to the original author(s) and the source, provide a link to the Creative Commons license, and indicate if changes were made.
In cases where reviewers are anonymous, credit should be given to 'Anonymous Referee' and the source.
The images or other third party material in this Peer Review File are included in the article's Creative Commons license, unless indicated otherwise in a credit line to the material. If material is not included in the article's Creative Commons license and your intended use is not permitted by statutory regulation or exceeds the permitted use, you will need to obtain permission directly from the copyright holder.
To view a copy of this license, visit https://creativecommons.org/licenses/by/4.0/

NCOMMS-25-70629 REVIEWER COMMENTS

Reviewer #1 (Remarks to the Author):

This manuscript reports the synthesis and structural characterization of a Pu(III) organometallic complex and its distinctive reactivity in small-molecule bond cleavage. By combining experiment with DFT, the authors systematically compare the behavior of Pu(III), U(III), and Sm(III) complexes and propose a cooperative mechanism involving metal-centered reduction and sterically induced reduction (SIR). The study is original and important for understanding small-molecule activation by transuranium elements.
This work is, in principle, suitable for publication in Nature Communications. However, the theoretical calculations are insufficient and the computational results fail to satisfactorily explain the experimental results. Therefore, I recommend that the manuscript be *reconsidered after revision.* My detailed comments are as follows.

1. Computational methodology is described incompletely. Please specify, for each element, the effective core potential (ECP) and corresponding basis set employed, and state whether and how solvent effects were included in the calculations.
We appreciate the reviewer's comment and we have now clearly indicated in the computational details of the supporting information the different type of RECP that were used for small core (actinides and Sm) and large core (for sulfur) elements.

2. Please provide full TDDFT-simulated UV-Vis-NIR spectra (with oscillator-strength) rather than listing a few individual excitations, to enable direct comparison with the experimental band shapes. In the manuscript, the main band of 1-Pu is assigned as 5f→6d (λmax ≈ 632 nm), whereas for 3-U/4-U it is written as 6d→5f (λmax ≈ 422/398 nm). How should the "6d→5f transition" be interpreted? To facilitate the assignment of transition character, annotate Tables S2–S5 with the dominant MO character for each principal excitation (e.g., U 5f/6d, Pu 5f/6d, Cp π, etc.).
In these calculations, we computed more than 2000 excited states but did not include all these since this would add more than 100 pages to the SI. Our intention was to provide the computed transitions, oscillator strengths and excited state descriptions, for the most important experimentally observed transitions (*i.e.* mostly the d→f).
We are happy to follow the reviewer's suggestion to add the orbital description and depiction, and describe in greater detail the 6d to 5f nature of the UV-visible region for **3-U** and **4-U**. Indeed, in the Tables S4 and S5 for these transitions, the "6d" orbital on U is involved in bonding interactions with the Cp^{Me4} ligand. We have thus rephrased this.

3. Regarding the 5f–5f transitions, some of the results listed in Tables S2–S5 extend beyond the experimental spectral range. The limitations of TDDFT in treating strong correlation and spin–orbit coupling, as well as its unreliable prediction of intensities for Laporte-forbidden transitions, may compromise the conclusions. It is recommended that representative species be investigated using NEVPT2 or CASPT2 (including SOC) for 5f–5f transitions, and that simulated spectra be compared with the experimental data to strengthen the reliability of the assignments.
We appreciate the reviewers comment about performing additional CASPT2 or NEVPT2 calculations, but respectfully note that these are very complicated calculations, and probing the 5f-5f transitions in the TDDFT is not the central point of the paper. We agree that TDDFT is not accurate enough to describe the 5f-5f transitions, but it at least allows us to capture the main feature of the transitions. We hope to find sufficient compute time in the future to include additional optical spectral modelling as there is clearly much to do to

understand transuranic small molecule reactivity, and both the role of, and effect on, their electronic structures.

4. In Fig. 3a, the Pu spin density remains ≈5 (consistent with an f5 configuration). Please discuss whether Int.2Pu can be reasonably classified as Pu(IV), and why the *Pu spin density appears essentially unchanged in TS.1Pu. The oxidation state labels and the spin density shown in the figure are contradictory*. Re-examine the TS structure and computational reliability as needed. At present, spin-density evolution is shown only for Fig. 3a, please provide analogous data for Fig. 3b and Fig. 4a,b.
We would like to thank the reviewer for pointing out this contradiction in our analysis of **TS.1Pu**; our error came from the misassignment as we found a short Pu-S distance which we attributed to the formation of Pu(IV).
We completely reanalyzed the data for both U and Pu and find that the difference between the reactivity of Pu and Sm is still surprising, but due to the propensity to react, rather than the use of oxidation state change. We discuss this more carefully in the text now. The new Figures 3 and 4 in the main text clearly show the spin densities at each point, and the changes occurring in the metal-ligand bonds as the reactions progress: for the $(PhS)_2$ reductive cleavage, **Int2.Pu** (relabled as **^{Pu}Int4**) is indeed Pu(III). It has a short Pu-S distance as the Pu-S bond forms and one longer Pu-Cp bond as the Cp departs as a radical.

5. There appears to be a contradiction in the proposed mechanism for small-molecule activation by 1-Pu and 1-U. At present, the mechanisms are essentially identical except for the SIR step. However, based on the spin density analysis, the oxidation state of Pu in 1-Pu does not change during the reaction, whereas U in 1-U is oxidized to the +4 state. This raises the question of whether the two systems may actually follow different mechanistic pathways.
This is correct and as mentioned in the answer in point 4, the discussion has been changed in the main text.

6. A. The key SIR step from Int.2Pu to 2-Pu is not explicitly shown in Fig. 3a. Please provide the corresponding transition state, barrier, and reaction energy for this step.
We appreciate the comment; however, this is now a moot point as we are showing the loss of the Cp radical in the revised Figure 3a.

6. B. In addition, the fate of PhS• in the final conversion from Int.2Pu to 2-Pu is not clearly indicated. Could the authors clarify whether PhS• recombines to form PhS–SPh, thereby contributing to the μ-S–S(Ph) bridge in 2-Pu, or whether one (or both) fragments are released as byproducts? It would be helpful to include this clarification in the figure to ensure conservation of species and atomic balance.
We thank the reviewer for catching this error. We have changed Figure 3a to correct the molecular/fragment balance.

7. In Fig. 3b and Fig. 4a, the purpose of the final step from Int.2U to 3-U, and from Int.4U to 4-U, is unclear. The uranium product does not show any changes from Int.2U to 3-U (or from Int.4U to 4-U), so how can a pronounced decrease in the system's energetics of this step occur? The author should explain how the energetics (i.e., -33.4, -50.7 and -42.7, -57.7) is calculated and the fates of the PhS radical and PhN radical?
We thank the reviewer and have modified the two figures to improve clarity. In particular, we show that in the case of U, the last step is only the release of the SPh radical from **Int2.U**, while the last step for Pu is both the release of Cp-Cp dimer and PhSSPh from two **Int2.Pu**.

8. In Fig. 3, please use dashed bonds to indicate bonds being broken or formed in transition-state structures to improve clarity.
Done

9. The failure to locate a stable [SmIII(CpMe4)3(PhSSPh)] adduct or transition state is only briefly discussed. Please provide a more systematic analysis from both steric and electronic perspectives and assess whether the outcome may be limited by methodological choices (functional, dispersion and solvent models, initial guesses, convergence criteria, etc.).

We changed a variety of parameters in the computational search for coordination of the disulfide substrate to the Sm(III) center but found no evidence in any case. Therefore the reason for the brief discussion in the manuscript.

We have now revised the discussion to describe this, and suggest from comparisons with Pu that the Sm center does not show an initial coordination of the PhSSPh substrate since it is insufficiently polarizable, lacking d-orbital access, to enable the substrate to coordinate.

10. There are inconsistencies between figure/table numbers and citations (e.g., p.4 L96: Figures S17–S19; L100: Table S1; p.6 L162: Table S2; p.8 L201: Tables S3–S4). Please verify and standardize all numbering and cross-references throughout the manuscript.

Many apologies for these. Fixed.

Reviewer #2 (Remarks to the Author):

In the submitted manuscript, Keener, Rajeshkumar, and co-workers present a study of small molecule activation by a set of related cyclopentadienyl-ligated Pu, U, and Sm complexes. All metals begin in the formal +III oxidation state, placing this work in one typical sweet spot for organometallic systems of the f-block. The study can be distinguished, as demonstrated by a thorough literature section, as a quite uncommon study of small molecule activation at a Pu complex. Work of this type is scarcely conducted with transuranium elements, and thus it is quite notable.

In terms of technical quality, the work has been carried out to a very high standard. Spectroscopic work, XRD, and expert computational work has been utilized to answer the right questions in this work. I am particularly keen on the thorough and well-documented UV-vis-NIR spectra, which seem to be perennially overlooked or abused in literature studies of TRU compounds. I have very few suggestions or questions for the authors about their data—although a few minor points that I'd appreciate seeing addressed in minor revisions are given below in a list of all my specific points.

In terms of broad interest, I anticipate that the molecular mechanistic work presented here brings the paper over the bar for consideration/publication by Nature Communications, the current journal to which it has been submitted. I enjoyed seeing the concepts of SIR, electron transfer, and metal coordination dealt with in an even-handed way. Clearly, SIR is not yet well understood, and Pu still holds many secrets close that are yet waiting to be revealed.

To conclude, this is a solid paper on the chemistry of a novel Pu complex with comparisons to relevant U and Sm analogues. I suggest that it will be suitable for publication in Nature Comms after a round of minor revisions, with my specific points for the authors' consideration given below.

Otherwise, I thank the authors for their nice work and look forward to seeing it in print in due course.

-James Blakemore, University of Kansas

We thank the reviewer for the kind comment and appreciation of the work.

Specific points

1. How was the yield of the crystals of 1-Pu determined? Please mention this. Counting statistics or weighing mass?

We have modified the text in the SI to specify how the yields were determined.

2. How was the stability of 1-Pu in solution in toluene for "at least two month" determined? It could be useful to provide this data in the SI, given the observation of a hydrolysis product with bridging OH groups. One might hope for continuous spectral data over time, or periodic spectral data, but if it is manual checks by eye over time, so be it. I think it should just be mentioned how this was estimated.

We have clarified the text in the MS and SI and added the ^{1}H NMR stability studies, which can be found in Figure S7.

3. Above Figure 1, the authors compare the out of plane distortion of the methyl groups in 1-Pu and U(Cp*)3 and suggest that metrics in this regard suggest that 1-Pu is poised for SIR. Can this be unpacked a bit more in expanded or clarifying text? Superficially, the similarity of 1-U and 1-Pu suggests to me that these compounds are structurally simply quite similar.

Yes! We have modified and expanded the text in terms of the metrical parameters. We also needed to reword

as it appears the original text may have been hard to follow; in fact, we stated that based on the metrical parameters alone, in comparison to systems that exhibit SIR, **1-Pu** should be *more* poised for a metal-based reduction than SIR. We have clarified the text.

4. The authors are commended for their thorough work on the UV-vis-NIR spectral data collection. Having both the solid and solution data is a real treat! Nothing to change in the data or presentation, I just mention it is lovely.
We thank the reviewer and appreciate the comment.

5. Along the lines of the above, would the authors be willing to mention in their paper any available information regarding the following:
The 5f to 6d excitation in the Pu compounds appears quite distinctive, but I also see that the U compounds have absorption that is not dissimilar in intensity and energy. Are the U compounds showing similar signature of 5f to 6d transitions? This could be interesting to mention…very distinctive and one supposes these are largely driving the beautiful colors of these compounds which as shown in the SI photos accompanying spectra, quite intense.
I see that the 5f to 6d for the U species is mentioned on p.8. However, would the authors be willing to consider mentioning if the relative energies are indicating something about the f and d orbital energies for these compounds relative to the standard view of the 6d and 5f relative energies in standard discussions. The compounds here are analogous and I am interested in how the new spectra can be used to bolster understanding of where 6d to 5f features can be expected in a general sense.
We agree this would be interesting, but unfortunately the numbers would have no meaning. The discussion referred to derives from TDDFT computed excitations. Unfortunately, showing the energy of orbitals at the DFT level have no meaning since orbitals in DFT are only introduced by the Khon-Sham scheme in order to allow calculations to start on real systems rather than in the gas phase.

6. The authors mention "slight" decomposition of (PhNH)2 to azobenzene and other products upon mixing with 1-Pu for 14 days. What is the estimated conversion of the starting material to azobenzene, particularly based on apparent integration of the (PhNH)2 in NMR? (I am just asking for an estimate to be included in the final paper, and realize there might be issues that make the quantification less certain than desirable.)
We have modified the text in the MS and SI to include an estimated conversion of $(PhHN)_2$ to $(PhN)_2$.

7. In the Conclusion, the authors refer to Pu(III) as weakly reducing. I realize this is subjective language and at the authors discretion, but I personally do not consider Pu(III) to be weakly reducing but rather reasonably strongly reducing, at least when electrolytically produced in basic aqueous solution. Albeit it is less reducing than U(III) to the best of my knowledge. A general statement somewhere in the paper (or multiple places if relevant) about these relative notions and any mention of standard reduction potentials could be helpful to readers who are not experts in Pu chemistry to parse the results shown here.
We agree with the reviewer in that the language about reductive ability of U(III) versus Pu(III) is subjective; therefore, we have included a reference for standard reduction potentials of the actinides to give an idea of the relative capabilities of the ions.

8. Would the authors consider providing machine readable XYZ files for the computed structures with their paper? I always find these nice as they can be readily loaded into visualization programs, like CIFs.
Absolutely; these are being included in the Mendeley open data archive (29z869rwhb, in moderation) associated with the manuscript.

Reviewer #3 (Remarks to the Author):

Keener and co-workers reports small molecule activation by more or less sterically congested organoactinide complexes such as [An(CpMe4)3] (An = Pu, U), where the authors claims that the Pu complex exhibits a unique reactivity which has not been observed in the U analogue despite many structural similarities found between them. Syntheses and structural characterization for these compounds and reaction products have been thoroughly performed. Therefore, this work should be eligible to be publicized to a scientific community.

However, I still have several concerns in this manuscript as follows.
I do not know what is the novelty of this work, especially for the reductive cleavage of the S-S bond. This reaction is *quite ordinary as exemplified by the well-known cystine-cysteine conversion in biochemistry*. Needless to say, the thiol (-SH) groups of cysteines oxidatively forms the S-S (disulfide) bond to make cystine, which is reductively cleaved to return to the parent cysteines.
With respect, the reviewer's search for novelty appears to derive from their desire for us to cleave an entirely new bond, rather than understand the unexpected difference in the way that the transuranic molecule cleaves a bond when compared to its two surrogates, the earlier 5f uranium, and its 4f size analogue, samarium. This is the first time that TRU molecules have been used to study redox -mediated activation and reduction of small molecules. The technical challenges associated with handling transuranics of this nature (*i.e*. ones that are sufficiently reactive to be useful for such studies) required us to be careful and selective with our target small molecules, hence the choice of the two small molecules $(PhS)_2$ and $(PhHN)_2$, with their different redox potential and capacity to be safely handled in the facility. Furthermore, even redox potential data on these metal cations in their different oxidation states is scarce, and non-existent for isostructural sets of molecules. The relative reactivity provides important data on the pure, neutral molecules, which would not be conserved in a classical electrochemical setup, due to the high reactivity of these systems with electrochemical solvents and supporting electrolytes.

In any systems of U and Pu of this work, the final product of (PhS)2 is the same thiophenolates, right? The difference between these actinides is *observed solely in the molecular structures* of the final coordination compounds, that is, 2-Pu and 3-U, in which *thiophenolate(s) are commonly involved*.
Thiolate complexes **2-Pu** and **2-U** (not **3-U** as the reviewer mentioned, which we believe was a typo) are not analogous. We have a formal terminal U(IV)-SPh in **3-U** versus a dimeric bridging Pu(III)-$(SPh)_2$-Pu(III) in **2-Pu**, which means that how these complexes are formed are not analogous to each other, which is discussed in detail throughout the MS.
Furthermore, since this is the first example of a Pu-thiophenolate, we are unsure how to interpret the final comment of the reviewer.
We wish to emphasise that this manuscript is about unexpected relative reactivity of a rare set of three essentially isostructural f-block complexes, one of which, the Pu congener, is still barely studied in anaerobic, aprotic environments. We do our best to suggest the reasons for their surprising differences in reactivity, but note that this is the small molecule reactivity ever studied for air-sensitive plutonium chemistry, so there is really a lot to learn. This is really just the beginning, we hope.

Even if the reported small molecule activations is still novel in the organoplutonium chemistry, I wonder what role the Pu(III) center actually plays there. Considering the current reaction in the simplest manner, one of CpMe4^- ligands in the starting Pu complex (1-Pu) *could be a reductant* for (PhS)2 to afford thiophenolate and (CpMe4)2, where the Pu(III) center is just spectating due to its weaker reducing nature compared with U(III).
The reviewer is correct. The $(Cp^{Me4})^{1-}$ ligands can reductively cleave the $(PhS)_2$ molecule to yield a thiolate ligand and $(Cp^{Me4})_2$, in which the Pu(III) acts as a "spectator". This is what we discuss in detail as SIR reactivity. However, there is no information in the literature on the role of the metal–ligand interaction during a proposed

SIR mechanism*,* as this type of reactivity has not been mechanistically studied by computations in previous reports, *for either lanthanide or actinide complexes*. This was our goal in trying to better understand this type of chemistry. This is especially interesting to understand for plutonium, precisely because we do have a metal center that is redox active, versus the previous Ln(III) analogues that cannot access the Ln(III/IV) couple. We discuss this in detail in the MS.

According to the synthesis section, KCpMe4 is available in the authors' laboratory. What happens if (PhS)2 is mixed with KCpMe4? To reveal the difference in redox potentials of these trivalent actinide complexes, cyclic voltammetry must be done.
The reviewer brings up an excellent point. We have reacted KCp^{Me4} and $(PhS)_2$ and monitored the reaction by ^1H NMR spectroscopy. We found that even though there is reactivity between KCp^{Me4} and $(PhS)_2$, there is no formation of the $(Cp^{Me4})_2$ dimer, suggesting that the Pu(III) starting material is required to promote this type of reductive cleavage chemistry. We have modified the text in the MS and SI.
We agree with the reviewer's comment about performing electrochemical studies. This is something we are actively pursuing, but we believe the results stand alone and have warranted their own publication in the future. We have modified the text and mentioned that we are actively pursuing electrochemical studies.

For the 1H NMR spectra of Fig. S10 and related discussion in page 5, I wonder that how much (PhS)2 was actually consumed in this reaction. From the peak intensities in the NMR spectra, it is hard to know whether or not > 70% of (PhS)2 has been actually converted in this 7-day reaction, in which 1-Pu has fully disappeared. By the way, which is correct as a yield of 2-Pu, 70% in main text or 81% in SI?
Thank you for catching this. The SI has been modified to reflect the correct yield for **2-Pu**. Additionally, the reviewer brings up an excellent point, and we have modified the text in the SI to reflect why there is still a small amount of $(PhS)_2$ remaining in the ^1H NMR scale reaction.

Regarding the DFT calculations and Fig. 3 at page 9, what happens if "3-Pu", [Pu(CpMe4)3SPh], is assumed as a final product in Fig. 3(a)? Its stability must be compared with that of 2-Pu, otherwise it is impossible to claim the uniqueness of observed reactivity in this Pu system. This is also the case for Fig. 3(b). Is "2-U", [(U(CpMe4)2)2(mu-SPh)2], less-stable compared with 3-U? First of all, reaction kinetics should be studied experimentally in order to make such a mechanistic discussion, especially to know a rate-determining step of a reaction of interest.
As we understand this, the reviewer asking if the reaction can be modelled with the formation of "**3-Pu**" as a final product, as though the reaction proceed the same as for **U**. However, this complex is actually a relatively high energy intermediate in the reaction profile (labeled as ^{Pu}**Int4** in the new reaction profile, and 30.3 kcal/mol). It would thus not be possible for this to be a final product, and **2-Pu** is significantly more stable.
We very much appreciate the reviewer's interest in the possible formation of **2-U** as a final product, analogously to **2-Pu**. The computed structure of this was optimized and is included in the revised Figure 3b. As shown in the reaction profile, this bimetallic species is less stable than the monomeric, U(IV) complex **3-U** which is experimentally observed. The stabilization is due to the greater stability of the U^{IV} oxidation state than U^{III} in this case.
The reviewer makes an excellent suggestion to measure the reaction kinetics. We already included spectra of the reaction monitored by NMR spectroscopy at various timepoints (Figures S11), but we unfortunately cannot experimentally locate a rate determining step since we see no intermediates. Since we are not allowed, for safety reasons, to make **1-Pu** on a large-scale, it would take an unfeasible amount of time to properly interrogate the reaction kinetics and mechanism, and is beyond the scope of this project. We find that the DFT calculations, combined with our studies of the other complexes, provide a plausible mechanism.

Even at the end of discussion, crucial differences and uniqueness of Pu compared with U and Sm still remain unclear. Excuse me, the last 3 sentences are rather equivocal, because any terms of "new", "safe", "important", and "target" are unclear and not fully supported in this work.
We thank the reviewer for pointing out where a statement was too vague. We have revised the text here, and taken care to clarify our conclusions to point out where we are making a useful contribution to new knowledge.
This work represents the first small molecule activation chemistry for the transuranic element plutonium, as such marking a new area for organometallic chemistry, which has taught us so much about electronic structure and bonding across the rest of the periodic table.
As such, it provides new ways for us to compare and rank redox potentials of compounds that we would not be able to study normally by electrochemistry.
This study highlights how Sterically Induced Reduction (SIR), a reaction that has become widespread for lanthanide chemistry, but still not yet been studied computationally, does not simply translate from the 4f to 5f series, even though accepted wisdom suggests that Pu(III) should show ionic bonding and react just like Sm(III).

I also have several minor concerns listed below.
Page 2, line 41-43. It is confusing. What is an actual ligand in this episode of Sm? CpMe5 or CpMe4? In accordance with Scheme 1, CpMe4 would be correct, right?
We thank the reviewer for catching this typo, the text has been modified.

Page 5, line 121. I do not know why such a conclusion can be led. More detailed discussion or rationale must be described.
We have modified the text accordingly, as Reviewer 2 also asked us to expand on this. Please see our response to #3 of Reviewer 2's comments.

Page 8, line 209. There is no "Scheme 1b" in this manuscript.
This typo has been fixed and removed from the text.

Page 10, line 247. Does a single(CpMe4) occur as a byproduct?
This typo has been fixed; it is supposed to be $(Cp^{Me4})_2$.

Reviewer Comments

Reviewer #1 (Remarks to the Author):

The authors have adequately addressed the concerns raised in my initial review. The revised manuscript improves the presentation of computational methodology, strengthens the TDDFT spectral analysis, and enhances the consistency of the mechanistic illustrations. Overall, the computational results are well supported and show clear agreement with the experimental data. I recommend the manuscript for publication in this journal.

We appreciate the positive comments of Reviewer #1.

Reviewer #2 (Remarks to the Author):

The authors appear to have fully responded to all of my comments, and carefully responded to those of the other reviewers as well. (I am not a computational chemist and therefore cannot comment on the detailed points raised in that realm, however.) I recommend this article be moved along toward publication once the computational replies have been checked and those details confirmed.

We appreciate the positive comments of Reviewer #2.

Reviewer #3 (Remarks to the Author):

Regarding my first concern about novelty of the current work, it is a pity that little was eventually answered. I'm aware that this is the first report that TRU molecules have been used to study redox-mediated activation and reduction of small molecules. But, I had to think "so what?", because exploring such scientific frontiers is quite ordinary. While I understood how the authors are passionate to this chemistry, I cannot still feel any new and strong impacts from this work in terms of both science and technology.

We appreciate the Reviewer's opinion, but we provided what we considered straightforward reasons for why this new work is importance. Small molecule activation chemistry has revolutionized our understanding of d- and f-block chemistry elsewhere, and this is the first small molecule activation characterization for a transuranic molecule. The lack of understanding of how ligands affect metal redox properties in the 5f, the complete absence of small molecule activation by molecular plutonium compounds, the surprising contrast in reactivity between Sm and Pu, including the unexpected reactivity of Pu in spite of its less congested nature compared to the Sm congener. The work provides new and safe ways to install new ligands at Pu centers, and characterizes the first molecular Pu thiolato compounds. SIR, the type of reactivity that this Pu complex is engaging in, can enable molecules to engage in reductive chemistry they would have previously been considered incapable of, this is important for our safe handling of transuranic materials and nuclear waste legacies. We have incorporated some additional discussion into the manuscript to confer these points.

As I pointed in Comment 3 and the authors actually agreed, the Pu(III) center does not prominently work for the S-S bond cleavage of (PhS)2 with the reducing (CpMe4^-) ligand. Regarding Comment 6 for DFT calculations, the authors' point seems to be wrong. The energy value (30.3 kcal/mol) of the intermediate state labeled "PuInt4" consists of both the Pu(III) species and PhS• radical, right? Please, be sure that it is not "3-Pu" of Pu(IV), and cannot be compared with 3-U of U(IV). In addition, the PhS• radical should be excluded from the energy calculation. In summary, I would think this work is suitable for publication from a more specialized journal after additional revision.

We thank the reviewer for asking for clarification, and respectfully point out that it is essential that the Pu^{III} center is there, as the reaction *does not occur* for the Sm analogue, which would have been predicted to react. This result is underscored by the absence of reactivity in the control reaction.

We can confirm that the intermediate $^{\mathbf{Pu}}$**Int4** contains a Pu^{III} center and is not the unseen Pu^{IV} complex **3-Pu**. The unpaired spin density is given in Figure 5 (4.99). Perhaps the reviewer missed the discussion text, and the drawing of the intermediate in which we show the long distance to the departing Cp that is becoming a radical. Because of this, we also include the SPh radical in the calculation.

Additional comments to R1:

Comment 4. I'm happy to hear the authors tested the reaction between KCpMe4 and (PhS)2. Did you finally observe the S-S bond cleavage even under absence of any metals? The NMR spectra related to the discussion at line 145-147 should be included in Supporting Information.

As stated in lines 145-147, we did not observe reductive bond cleavage and **1-Pu** is needed to undergo SIR reactivity. The ^{1}H NMR spectra are in the SI.

Comment 5. If the (PhS)2 loadings exceeded the desired one, it is impossible to write "0.5 equiv. of (PhS)2" in the figure caption. The authors should calculate the actual amount of (PhS)2 in the NMR sample.

We appreciate the reviewer's comment and understand the concern. However, we clearly state in the SI that the slight excess observed in the NMR scale reaction is most likely due to a weighing error for these small amounts in the TRU glovebox. This is an NMR scale reaction that the reviewer is referencing. We know that the stoichiometry of the reaction is "0.5 equiv. of $(PhS)_2$" due to the larger scale reactions in which we observe full consumption of the starting materials.
